# Supplementary material for: Osmotic signaling releases PP2C-mediated inhibition of Arabidopsis SnRK2s via the receptor-like cytoplasmic kinase BIK1
Source: EMBO J. 2024 Oct 21;43(23):6076–103. doi: 10.1038/s44318-024-00277-0 (PMC11612456; doi:10.1038/s44318-024-00277-0)
Supplement: Supplementary file 1 — Appendix [file 44318_2024_277_MOESM1_ESM.pdf]

Appendix for

Osmotic signaling releases PP2C-mediated inhibition of *Arabidopsis* SnRK2s via the receptor-like cytoplasmic kinase BIK1

Guo-Jun Li[1,2]†, Kong Chen[1,2,3]†, Shujing Sun[1], Yang Zhao[1,2*]

Author for correspondence: Yang Zhao (yangzhao@psc.ac.cn)

Table of contents:

Appendix Figure S1. Protein expression levels of SnRK2.6 and PP2Cs in SnRK2.6-PP2C interaction assays……………………………………………………………..…………Page 1

Appendix Figure S2. Genotyping of *proSnRK2.6:SnRK2.6* transgenic plants……………………………………………………………..……………..……Page 2-3

Appendix Figure S3. The Y-to-D and Y-to-E point mutations of SnRK2.6 can hardly mimic the phosphorylation of the two conserved Tyr residues………………………………………Page 4

Appendix Figure S4. The rosette growth of the bik1 mutant was severely inhibited by osmotic stress……………………………………………………………………………………Page 5

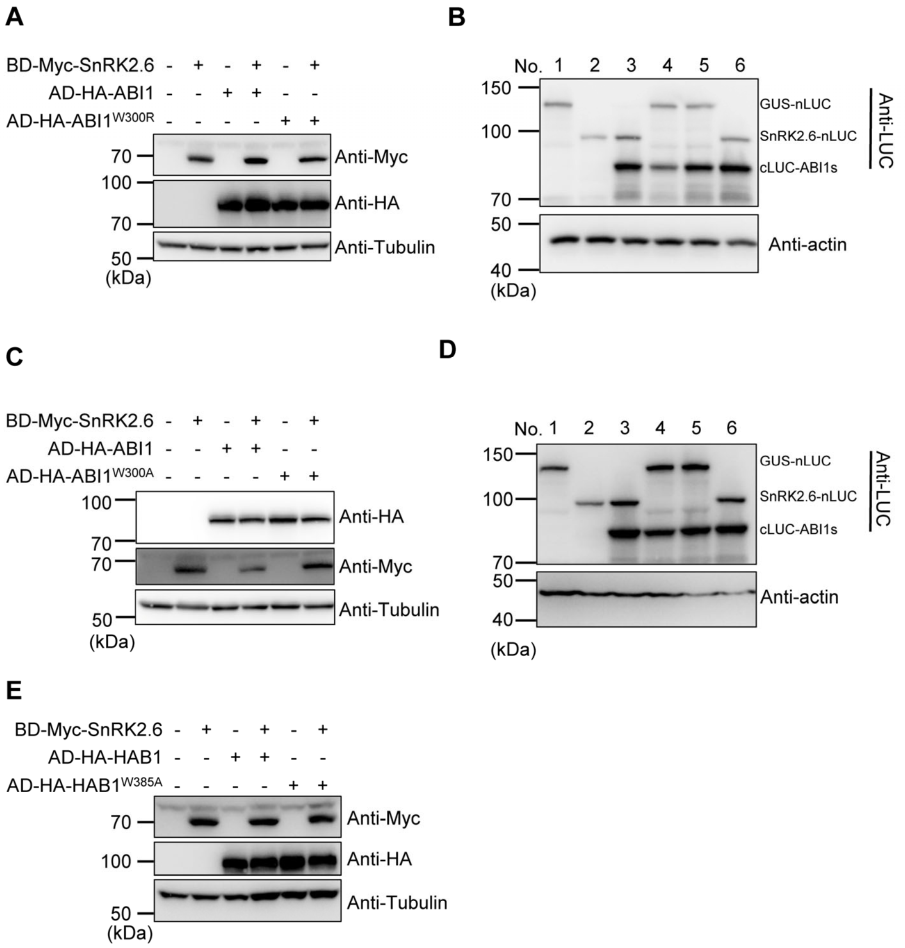

Appendix Figure S1. Protein expression levels of SnRK2.6 and PP2Cs in SnRK2.6-PP2C interaction assays.

(**A**, **C**, and **E**) Protein expression levels of BD-Myc-SnRK2.6, AD-HA-ABI1, AD-HA-ABI1^{W300R}, AD-HA-ABI1^{W300A}, AD-HA-HAB1, and AD-HA-HAB1^{W385A} in yeast cells for Y2H assays. Total proteins were extracted from yeast Gold cells and detected by western blot using anti-HA and anti-Myc antibodies. The anti-tubulin antibody was used as a loading control. The resulting yeast growth in the Y2H assays is shown in Figures 5A (corresponding to Appendix Figure S1A), 5C (corresponding to Appendix Figure S1C), and 5E (corresponding to Appendix Figure S1E).

(**B** and **D**) Total proteins were extracted from the *Nicotiana benthamiana* leaves expressing GUS-nLUC, SnRK2.6-nLUC, cLUC-FER ECD, and wild-type and mutated cLUC-ABI1, and detected by western blot using anti-LUC antibody. The anti-actin antibody was used as a loading control. The resulting LUC signals in the split LUC complementation assay are shown in Figures 5B (corresponding to Appendix Figure S1B) and 5D (corresponding to Appendix Figure S1D).

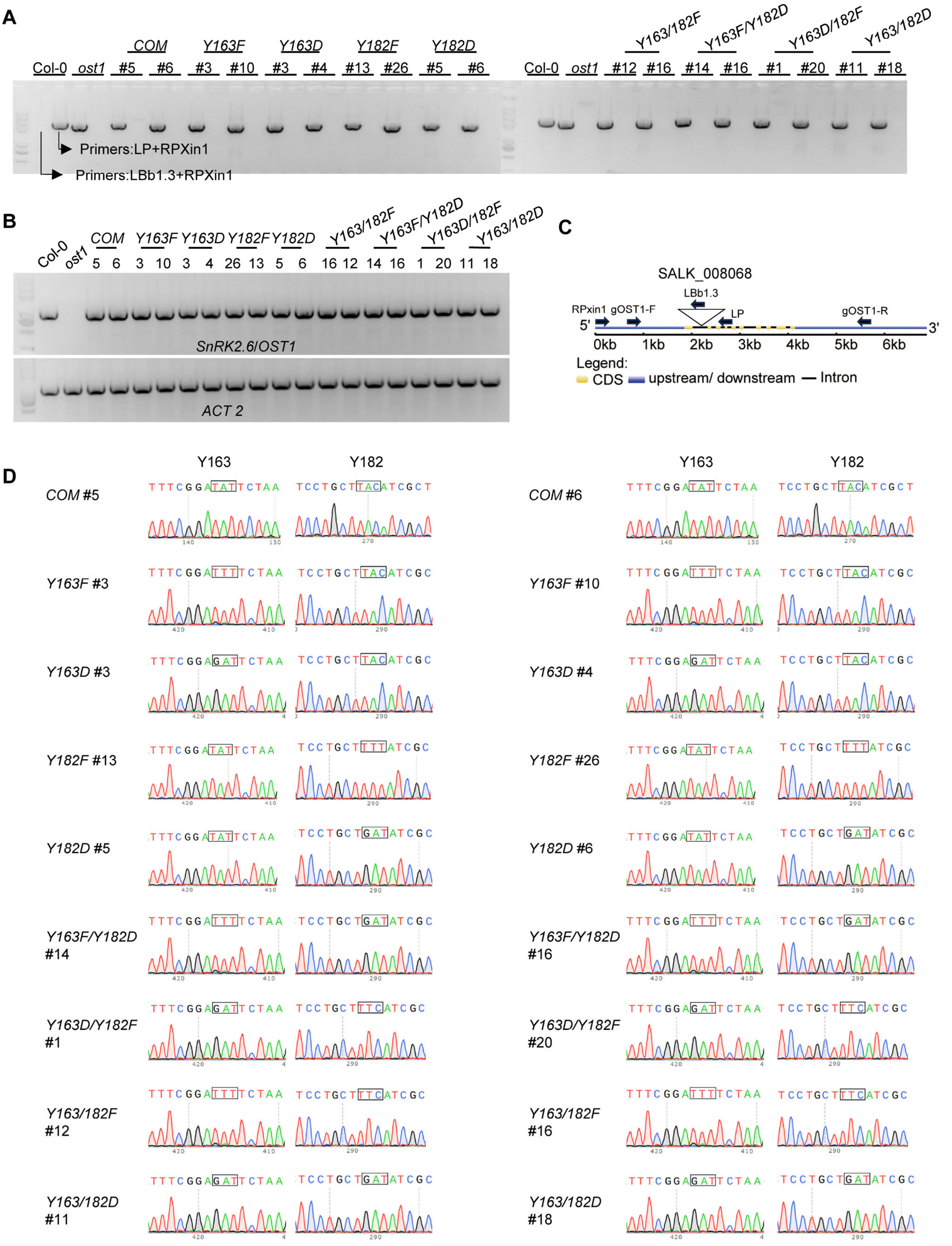

Appendix Figure S2. Genotyping of *proSnRK2.6:SnRK2.6* transgenic plants.

(**A**) The *ost1-3* mutant background and various *proSnRK2.6*:*SnRK2.6* transgenic lines were confirmed

by PCR analyses.

(**B**) The expression level of *SnRK2.6* in the *proSnRK2.6*:*SnRK2.6* transgenic lines was analyzed by RT-PCR. *ACT2* was used as an internal reference.

(**C**) Schematic representation of the SnRK2.6 loci, the T-DNA insertion site, the *proSnRK2.6:SnRK2.6* transgenic fragment, and the RPxin1 primer.

(**D**) Sanger sequencing chromatograms indicate mutations on *SnRK2.6* in *proSnRK2.6*:*SnRK2.6* transgenic lines.

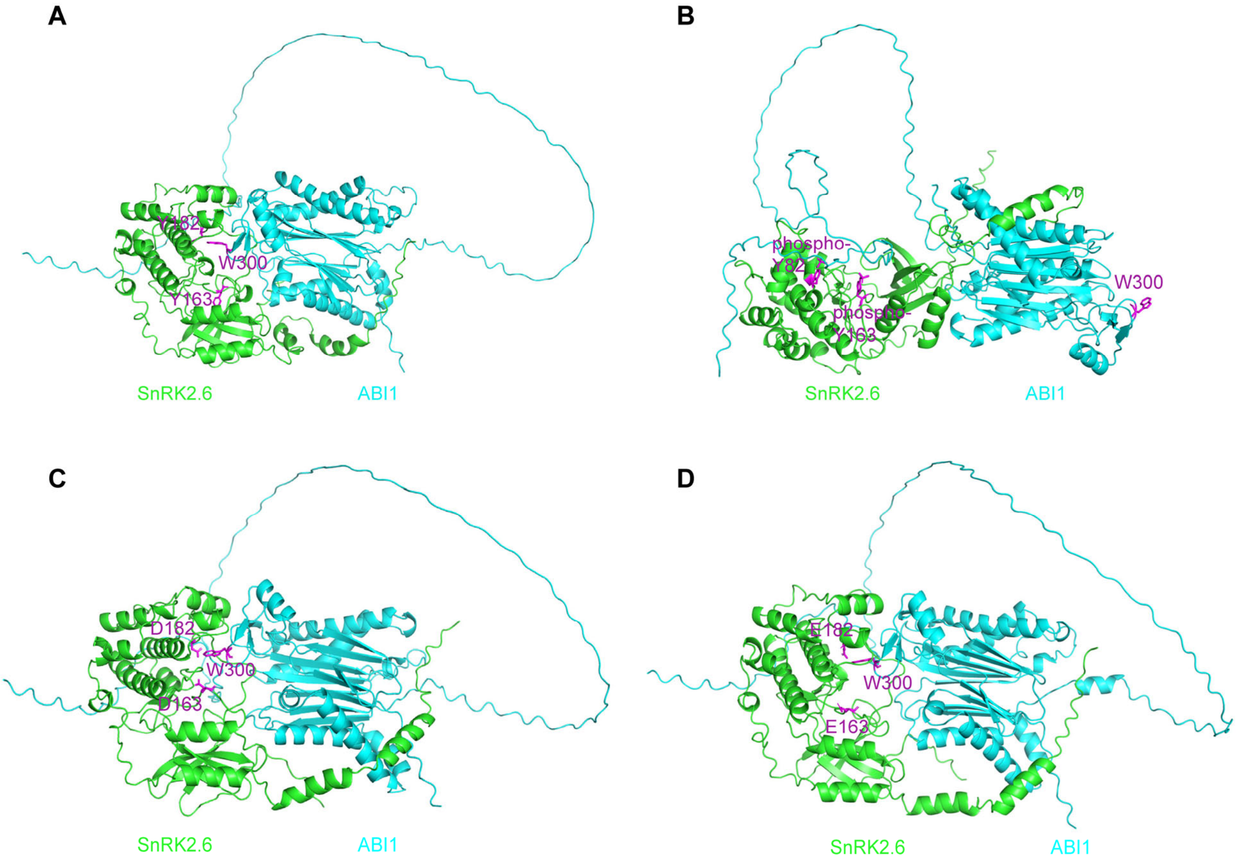

Appendix Figure S3. The Y-to-D and Y-to-E point mutations of SnRK2.6 can hardly mimic the phosphorylation of the two conserved Tyr residues.

Cartoon presentation of the SnRK2.6 and ABI1 interactions, without (**A**) or with modeling phosphorylation (**B**), or with the Y-to-D (**C**) and Y-to-E (**D**) point mutations, predicted by AlphaFold3. The interface highlights the key tyrosine residues (magenta, Y163 and Y182) in SnRK2.6 (green) and the tryptophan lock (magenta, W300) of ABI1 (cyan). Phosphorylation of the two tyrosines can increase the hydrophilicity and negative charges of the SnRK2.6 catalytic cleft and repel the docking of the nonpolar tryptophan lock of ABI1.

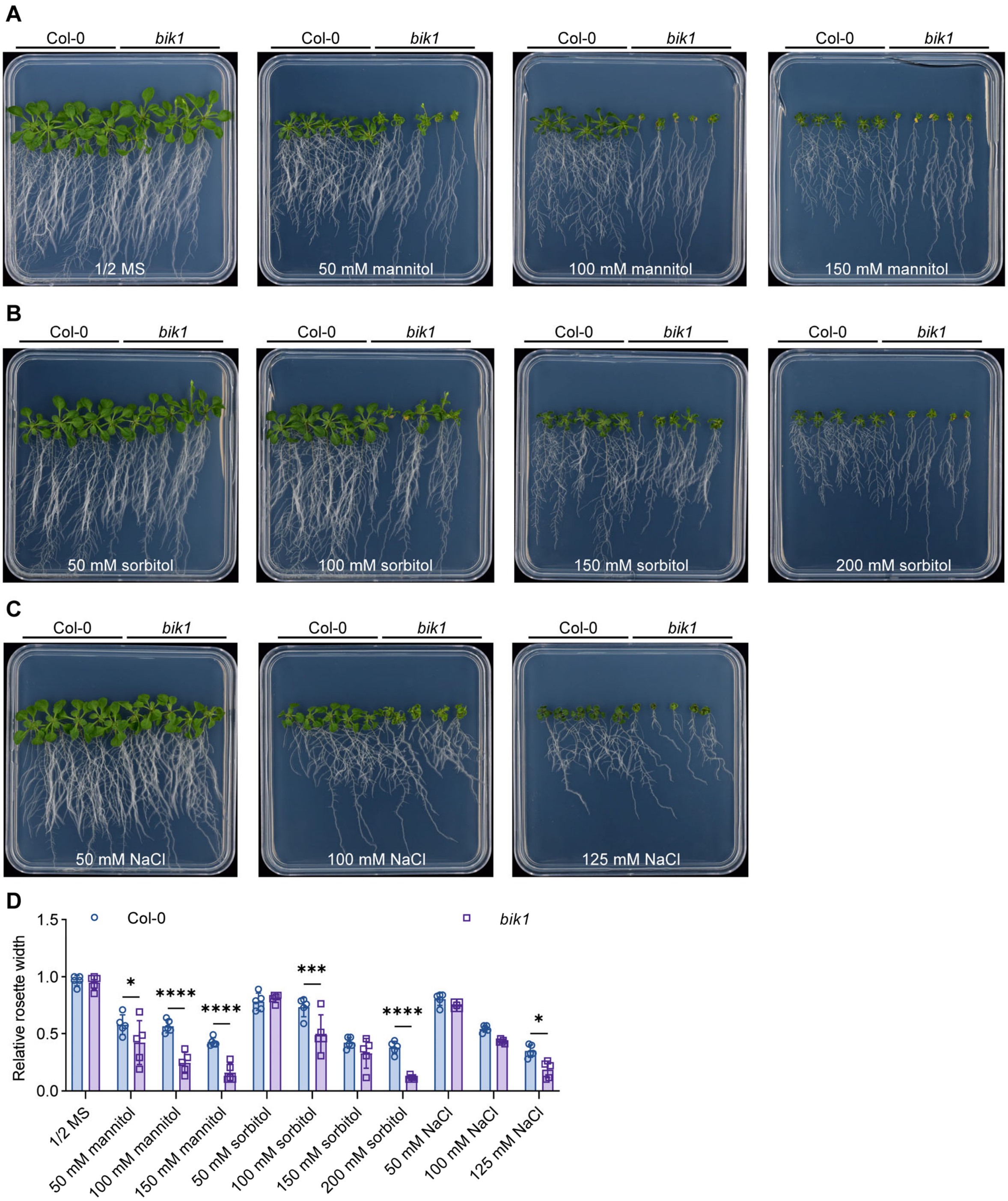

Appendix Figure S4. The rosette growth of the *bik1* mutant was severely inhibited by osmotic stress.

(**A-D**) Plant growth of WT and *bik1* mutant seedlings 15 days after the seedlings were transferred from ½ MS medium to ½ MS medium with or without different concentrations of mannitol (A), sorbitol (B), and NaCl (C). The relative rosette widths were quantified (D). Values are means ± SD ($n \geq 5$ seedlings). Statistically significant differences according to one-way ANOVA followed by Tukey's test. *$p < 0.05$, **$p < 0.01$, ***$p < 0.001$, ****$p < 0.0001$.
