# Supplementary figures and images for: Osmotic signaling releases PP2C-mediated inhibition of Arabidopsis SnRK2s via the receptor-like cytoplasmic kinase BIK1
Source: EMBO J. 2024 Oct 21;43(23):6076–103. doi: 10.1038/s44318-024-00277-0 (PMC11612456; doi:10.1038/s44318-024-00277-0)

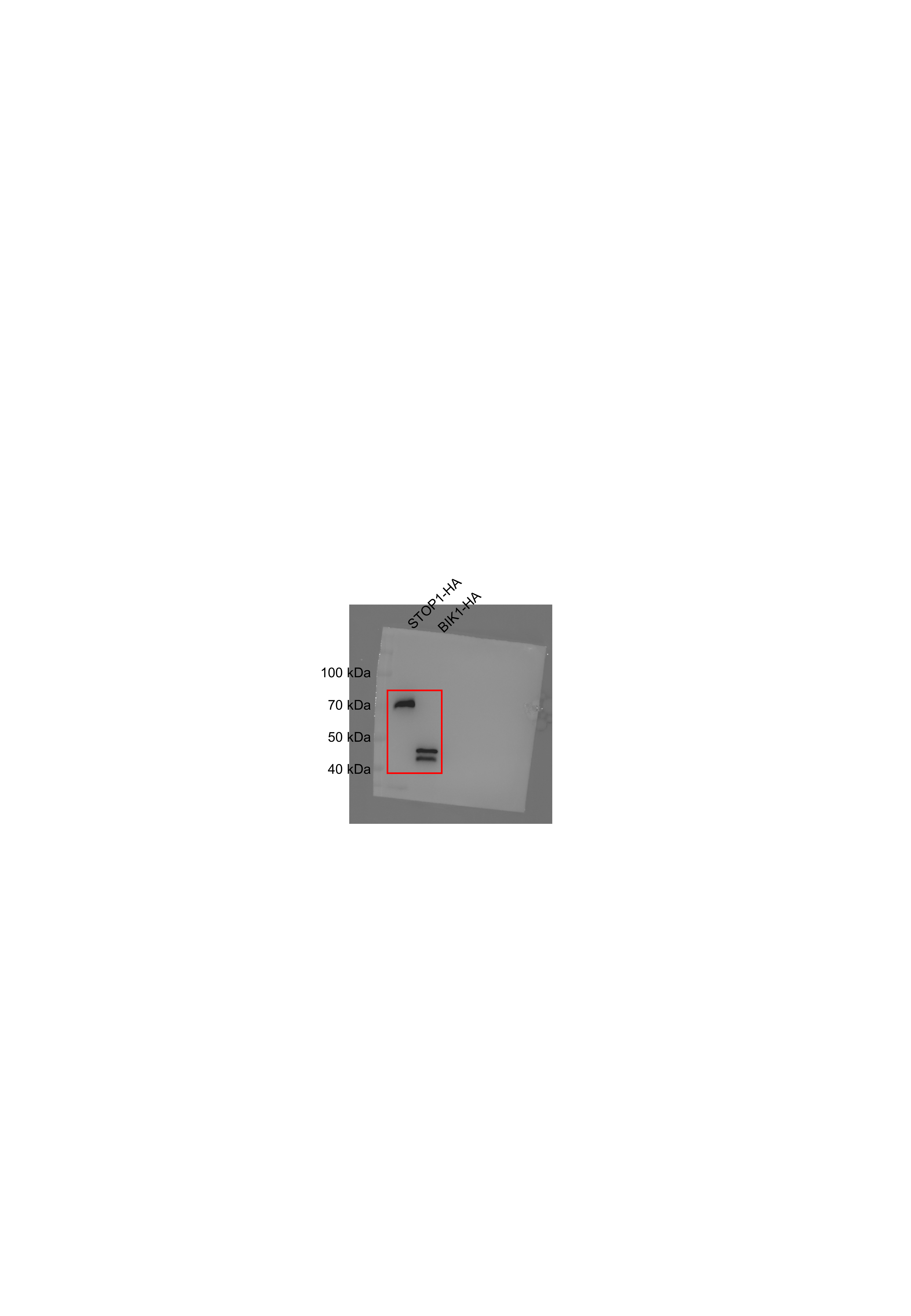

Supplement: Supplementary file 6 — Source data Fig. 1 [file 44318_2024_277_MOESM6_ESM.zip › SD figure 1/Figure 1C. IP anti-HA.tif]

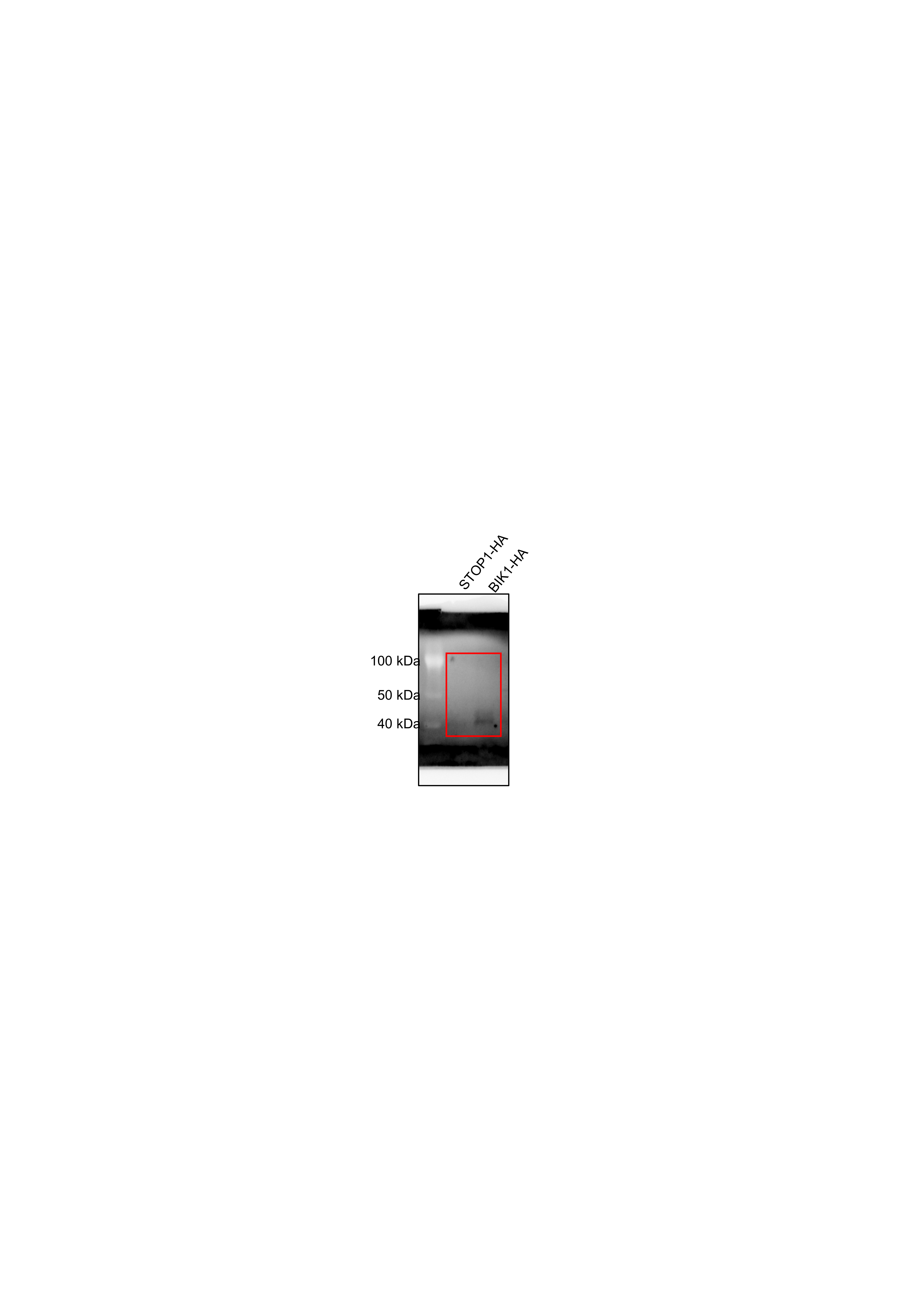

Supplement: Supplementary file 6 — Source data Fig. 1 [file 44318_2024_277_MOESM6_ESM.zip › SD figure 1/Figure 1C. IP anti-SnRK2.236.tif]

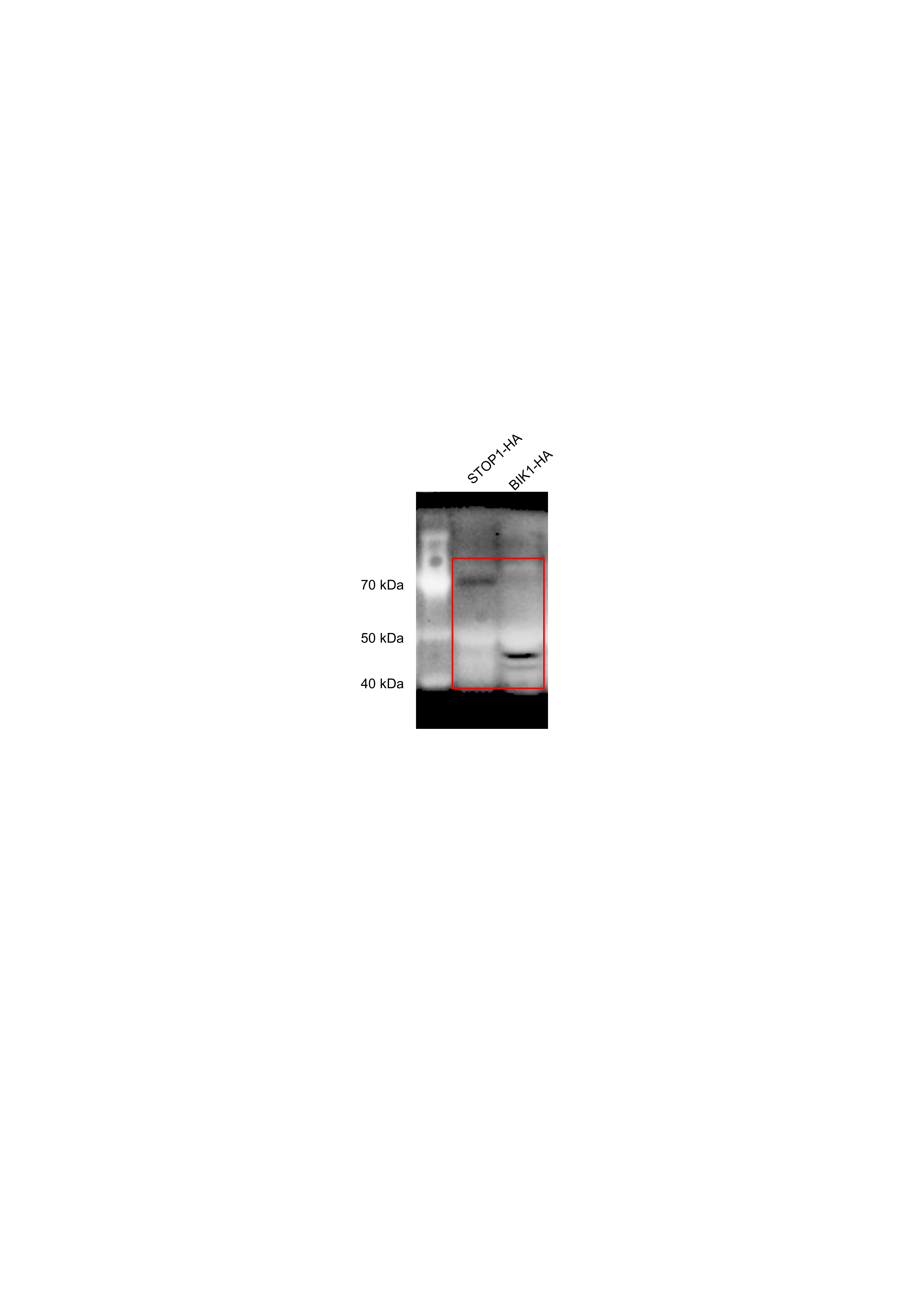

Supplement: Supplementary file 6 — Source data Fig. 1 [file 44318_2024_277_MOESM6_ESM.zip › SD figure 1/Figure 1C. input anti-HA.tif]

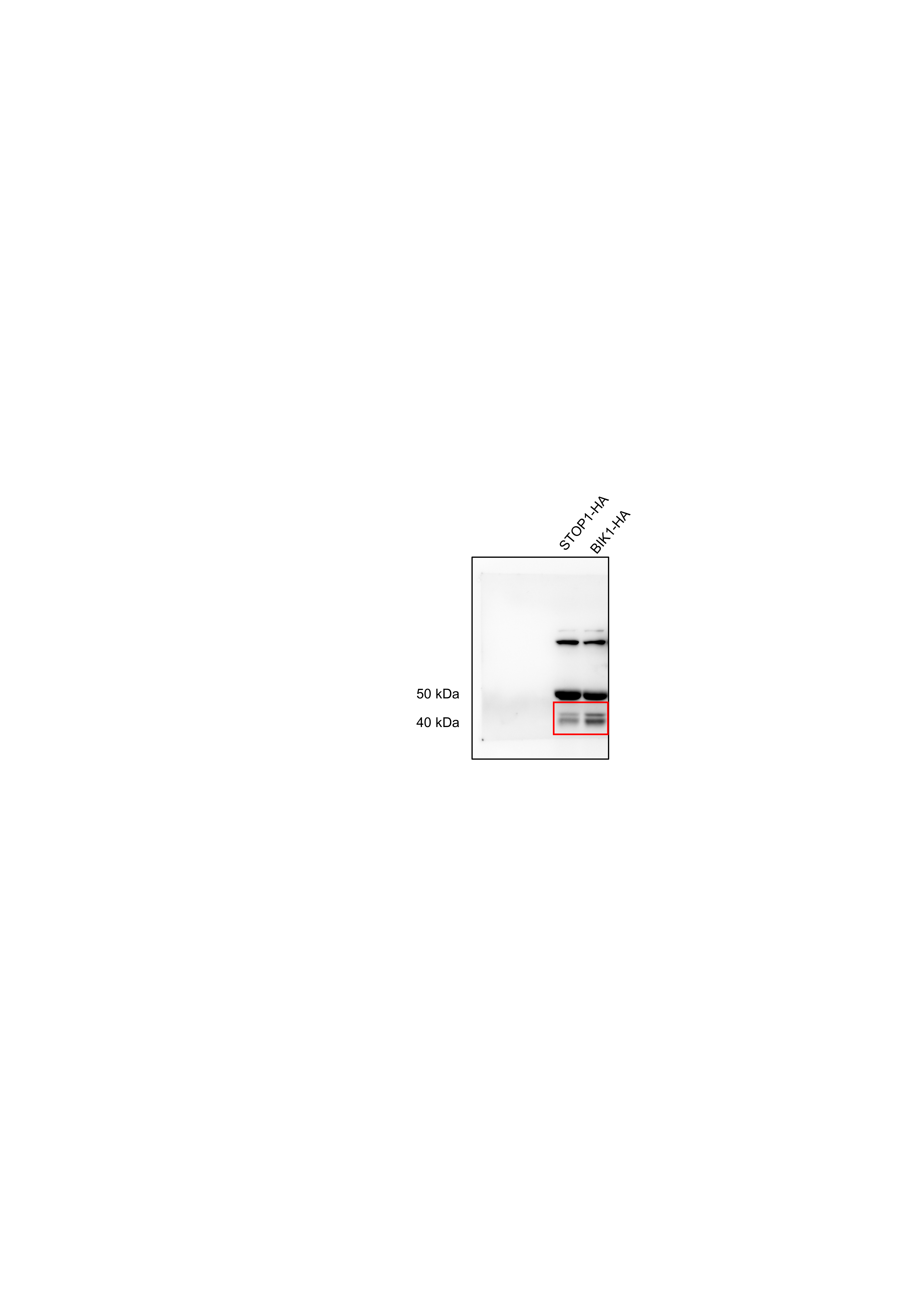

Supplement: Supplementary file 6 — Source data Fig. 1 [file 44318_2024_277_MOESM6_ESM.zip › SD figure 1/Figure 1C. input anti-SnRK2.236.tif]

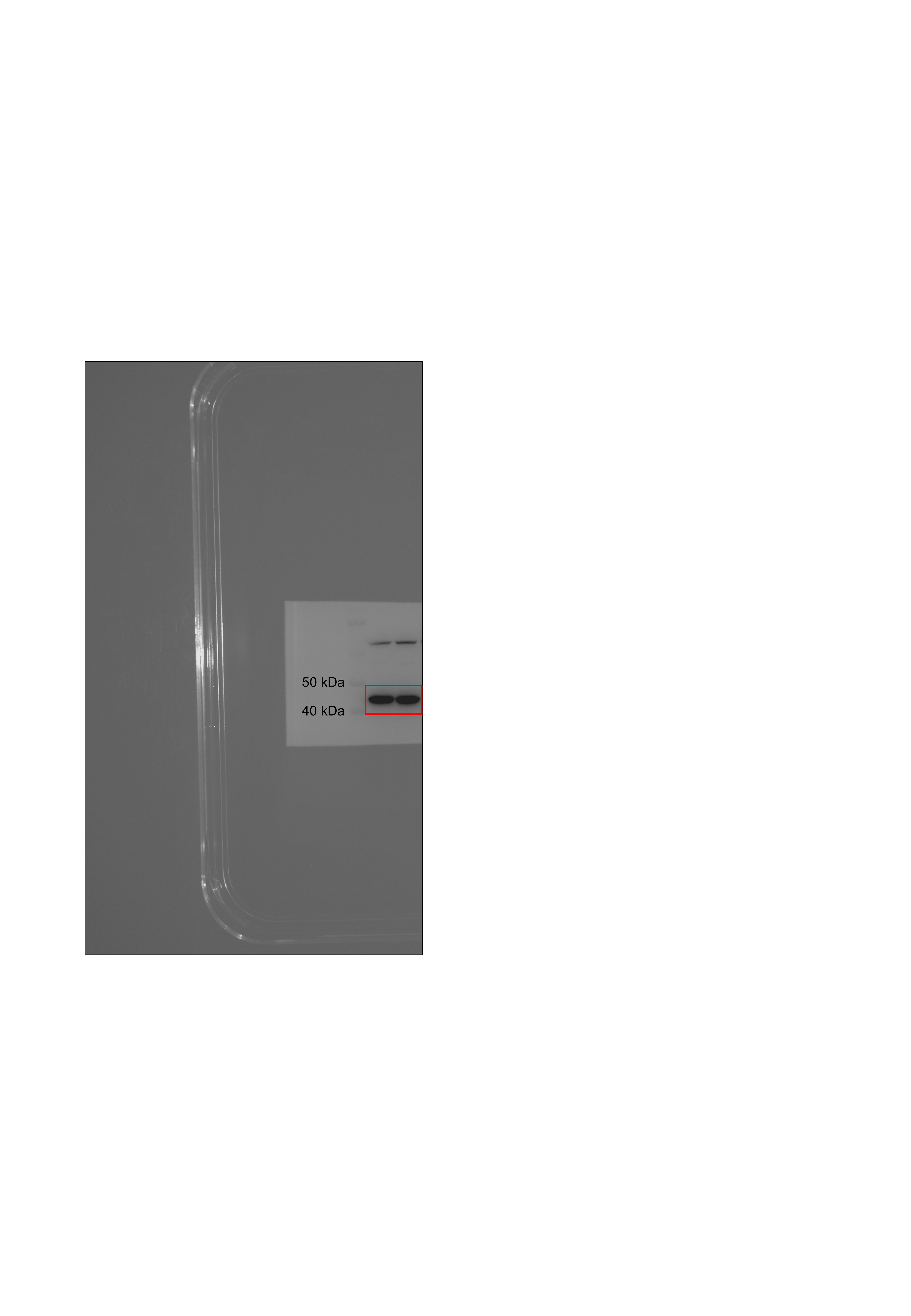

Supplement: Supplementary file 6 — Source data Fig. 1 [file 44318_2024_277_MOESM6_ESM.zip › SD figure 1/Figure 1C. input anti-actin.tif]

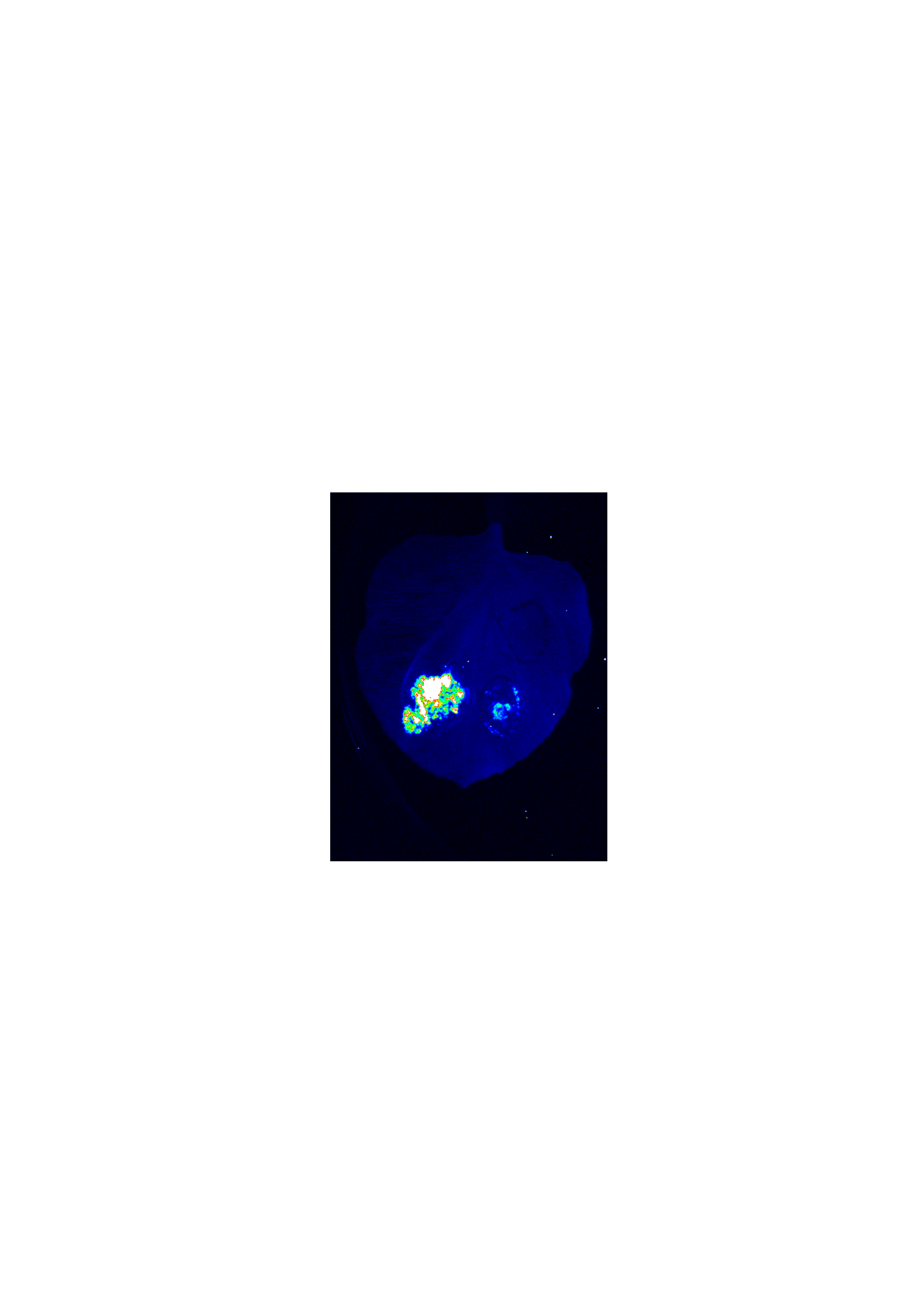

Supplement: Supplementary file 6 — Source data Fig. 1 [file 44318_2024_277_MOESM6_ESM.zip › SD figure 1/Figure 1D. split LUC.tif]

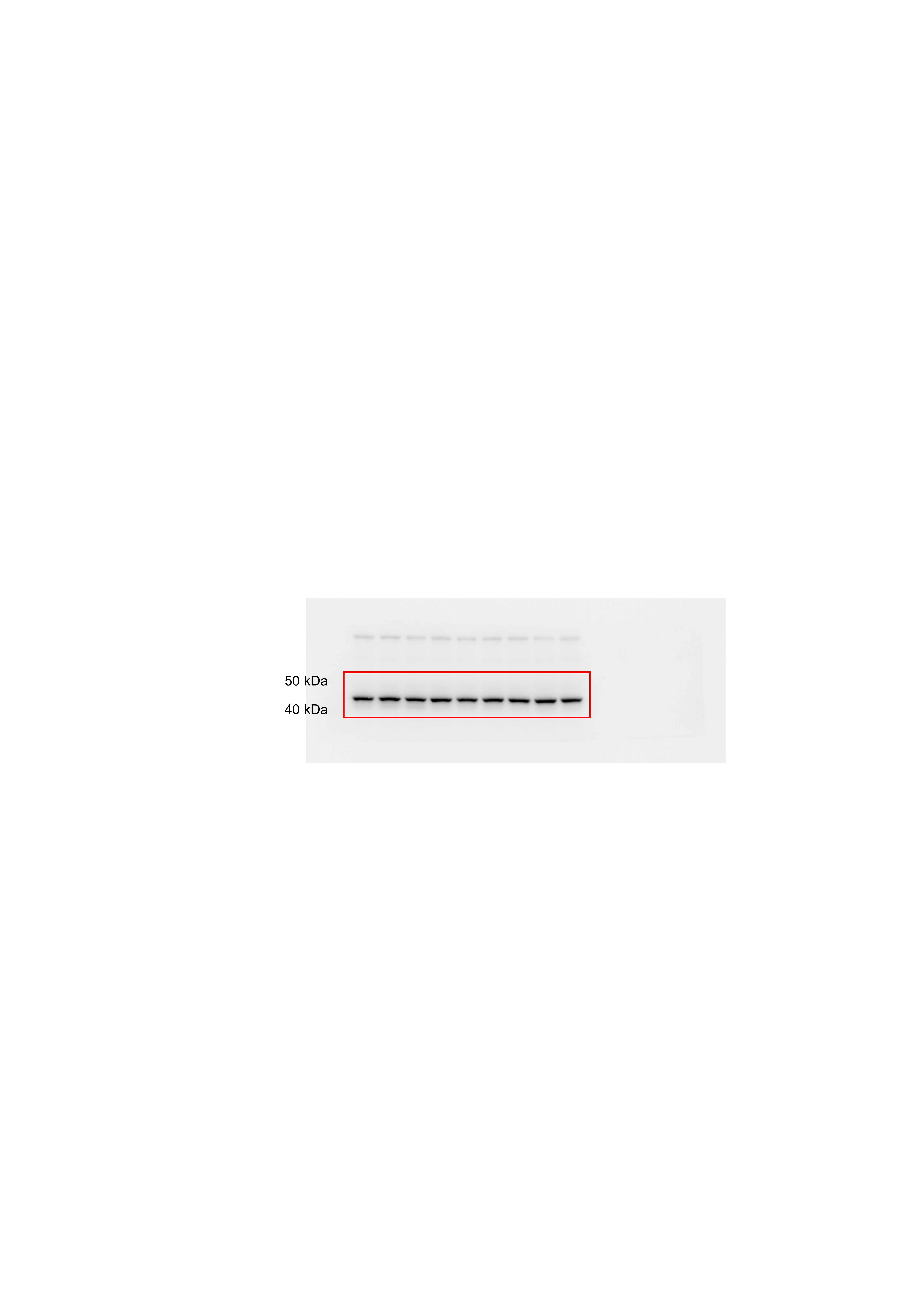

Supplement: Supplementary file 6 — Source data Fig. 1 [file 44318_2024_277_MOESM6_ESM.zip › SD figure 1/Figure 1E. anti-actin.tif]

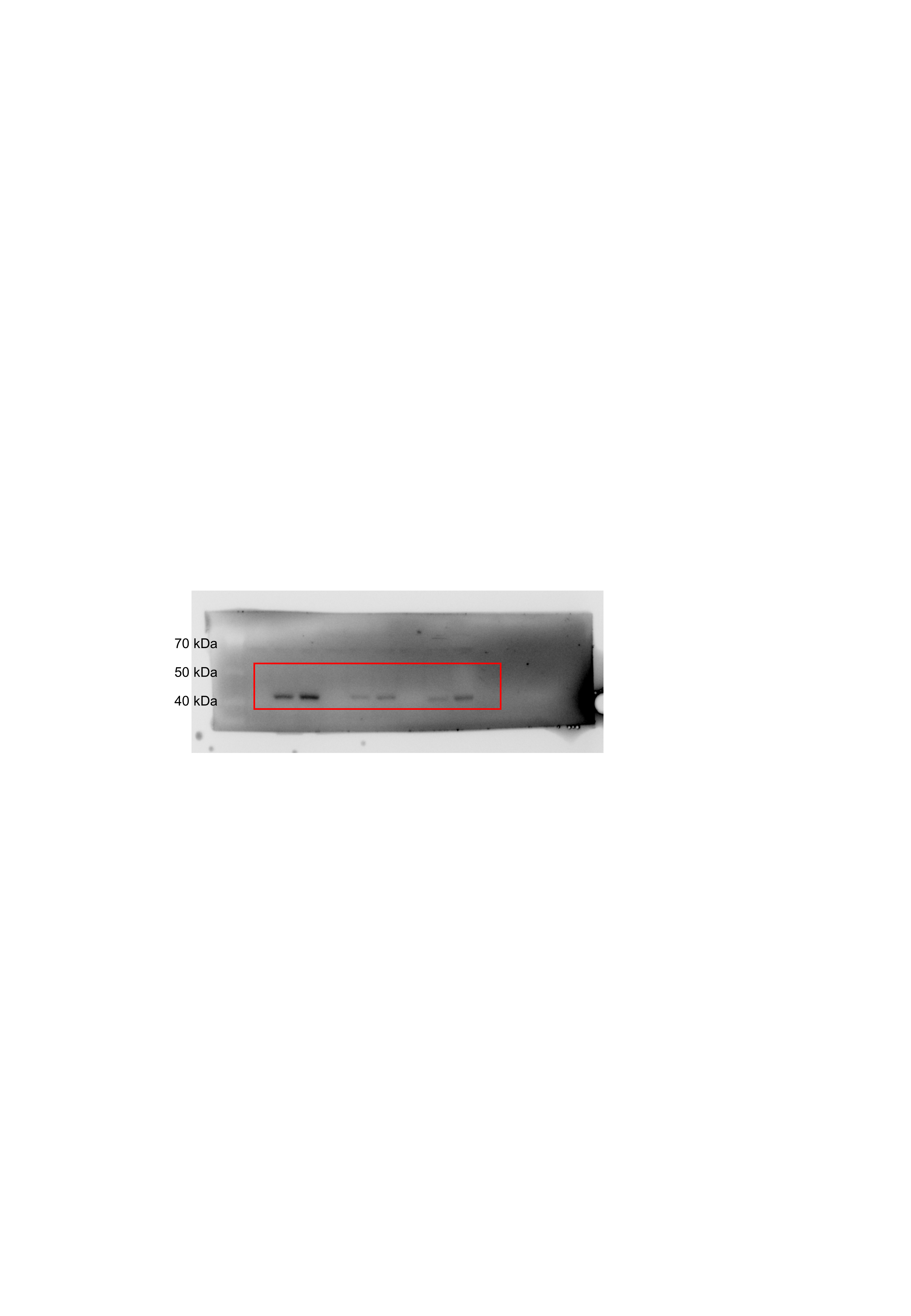

Supplement: Supplementary file 6 — Source data Fig. 1 [file 44318_2024_277_MOESM6_ESM.zip › SD figure 1/Figure 1E. anti-pS175-SnRK2s.tif]

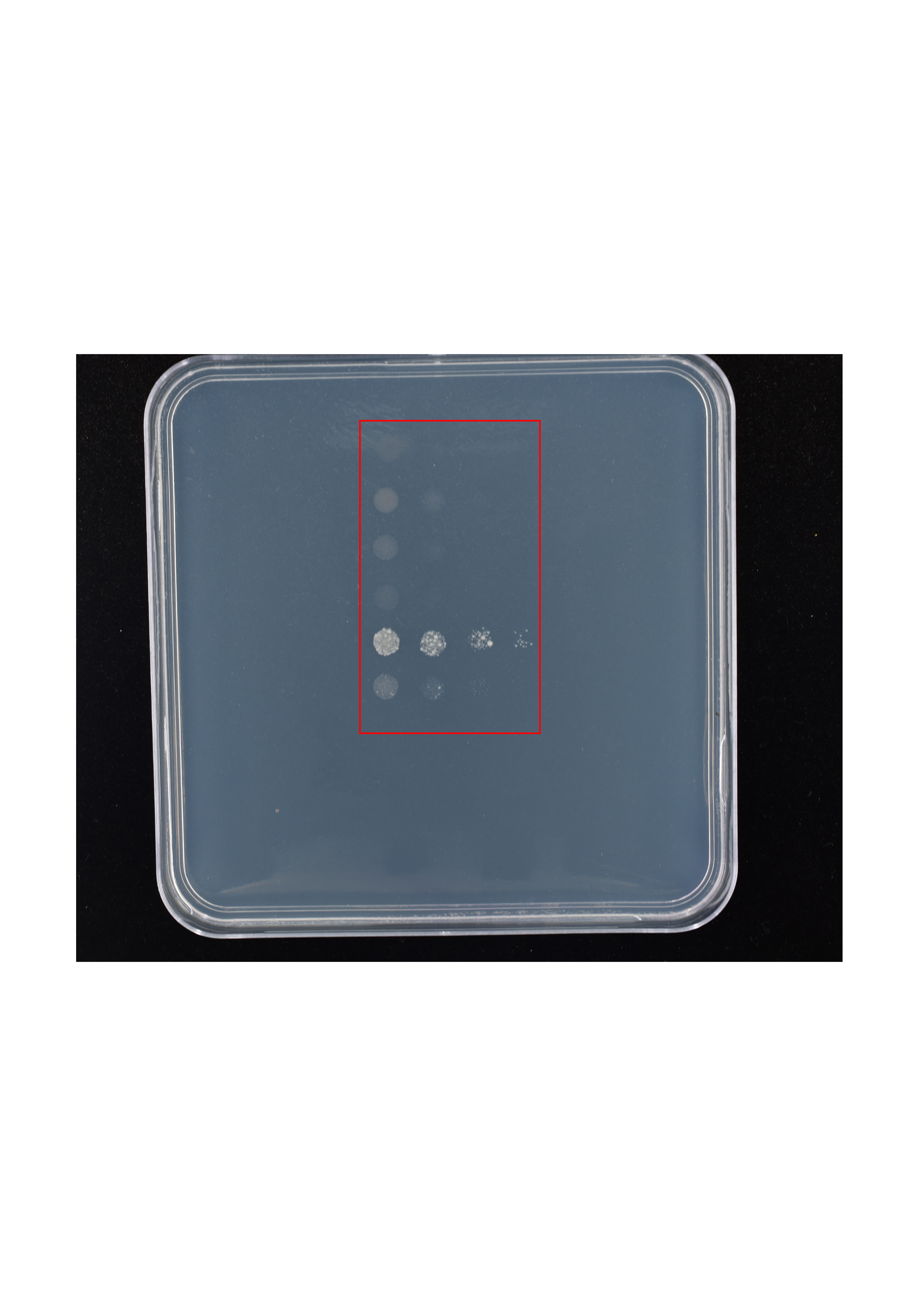

Supplement: Supplementary file 7 — Source data Fig. 2 [file 44318_2024_277_MOESM7_ESM.zip › SD figure 2/Figure 2A. Y3H, - ALWMH, with 1 M man.tif]

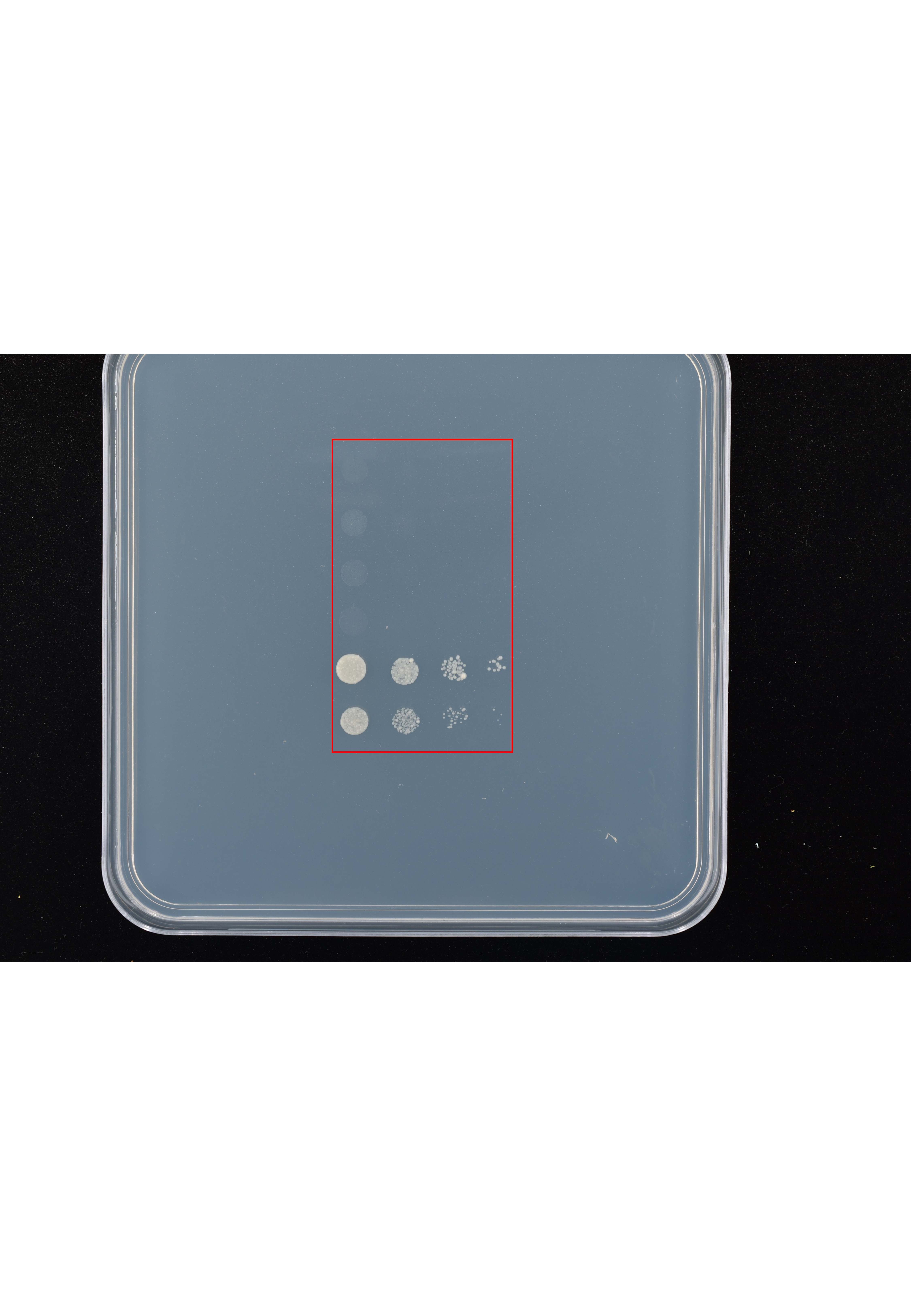

Supplement: Supplementary file 7 — Source data Fig. 2 [file 44318_2024_277_MOESM7_ESM.zip › SD figure 2/Figure 2A. Y3H, -ALWMH.tif]

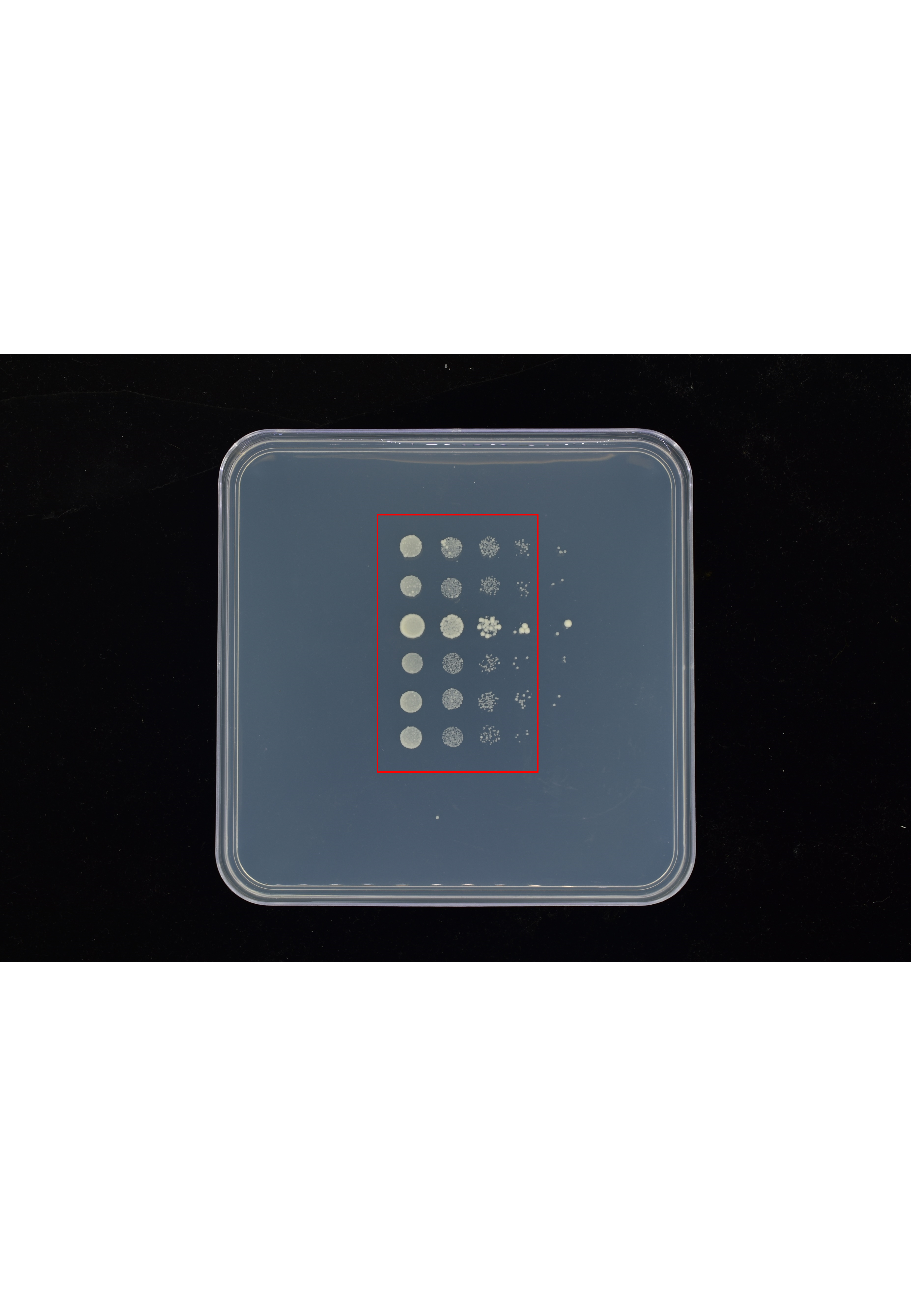

Supplement: Supplementary file 7 — Source data Fig. 2 [file 44318_2024_277_MOESM7_ESM.zip › SD figure 2/Figure 2A. Y3H, -LWM.tif]

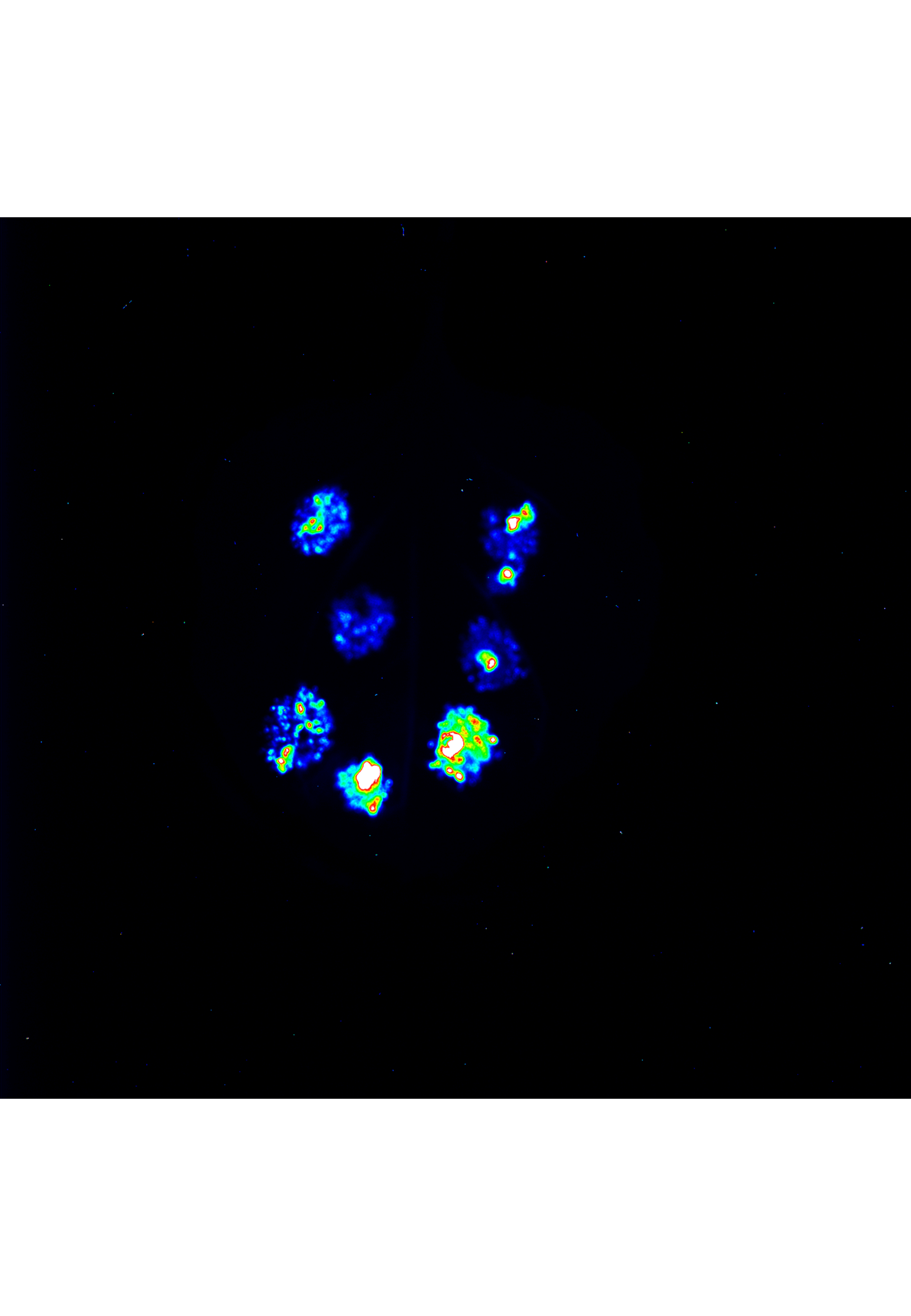

Supplement: Supplementary file 7 — Source data Fig. 2 [file 44318_2024_277_MOESM7_ESM.zip › SD figure 2/Figure 2B. split LUC.tif]

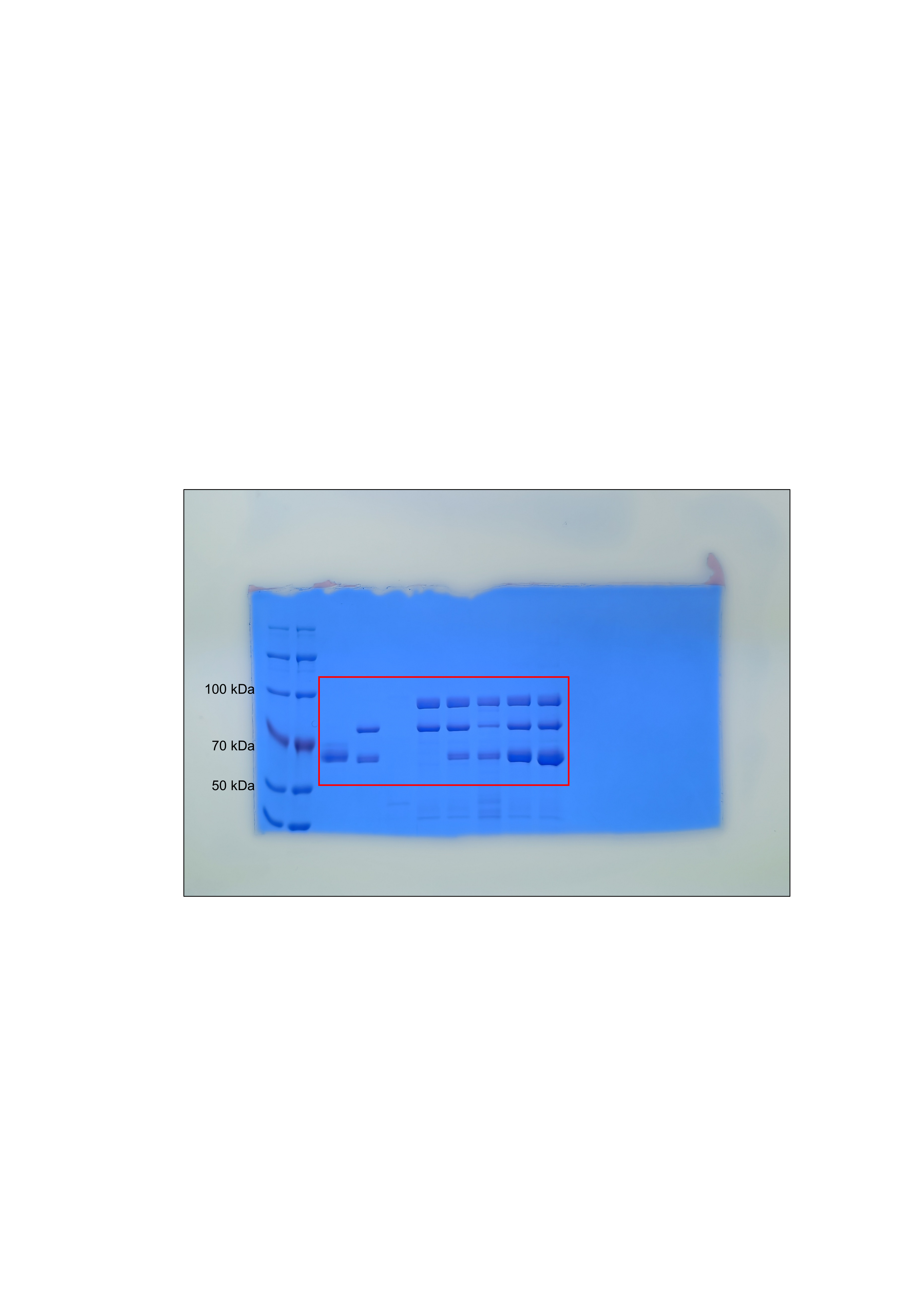

Supplement: Supplementary file 7 — Source data Fig. 2 [file 44318_2024_277_MOESM7_ESM.zip › SD figure 2/Figure 2C. CBB.tif]

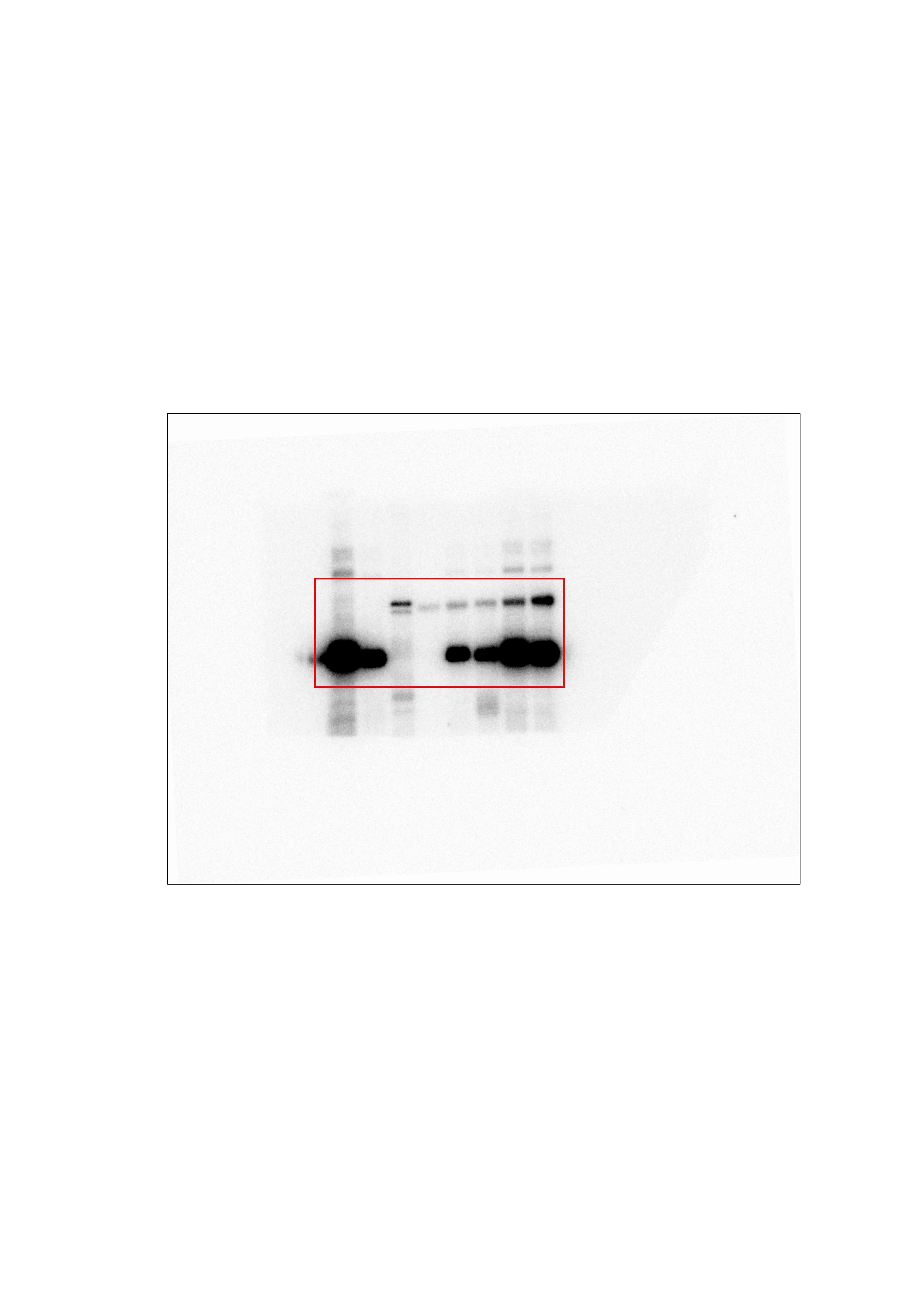

Supplement: Supplementary file 7 — Source data Fig. 2 [file 44318_2024_277_MOESM7_ESM.zip › SD figure 2/Figure 2C. auto-radiograph.tif]

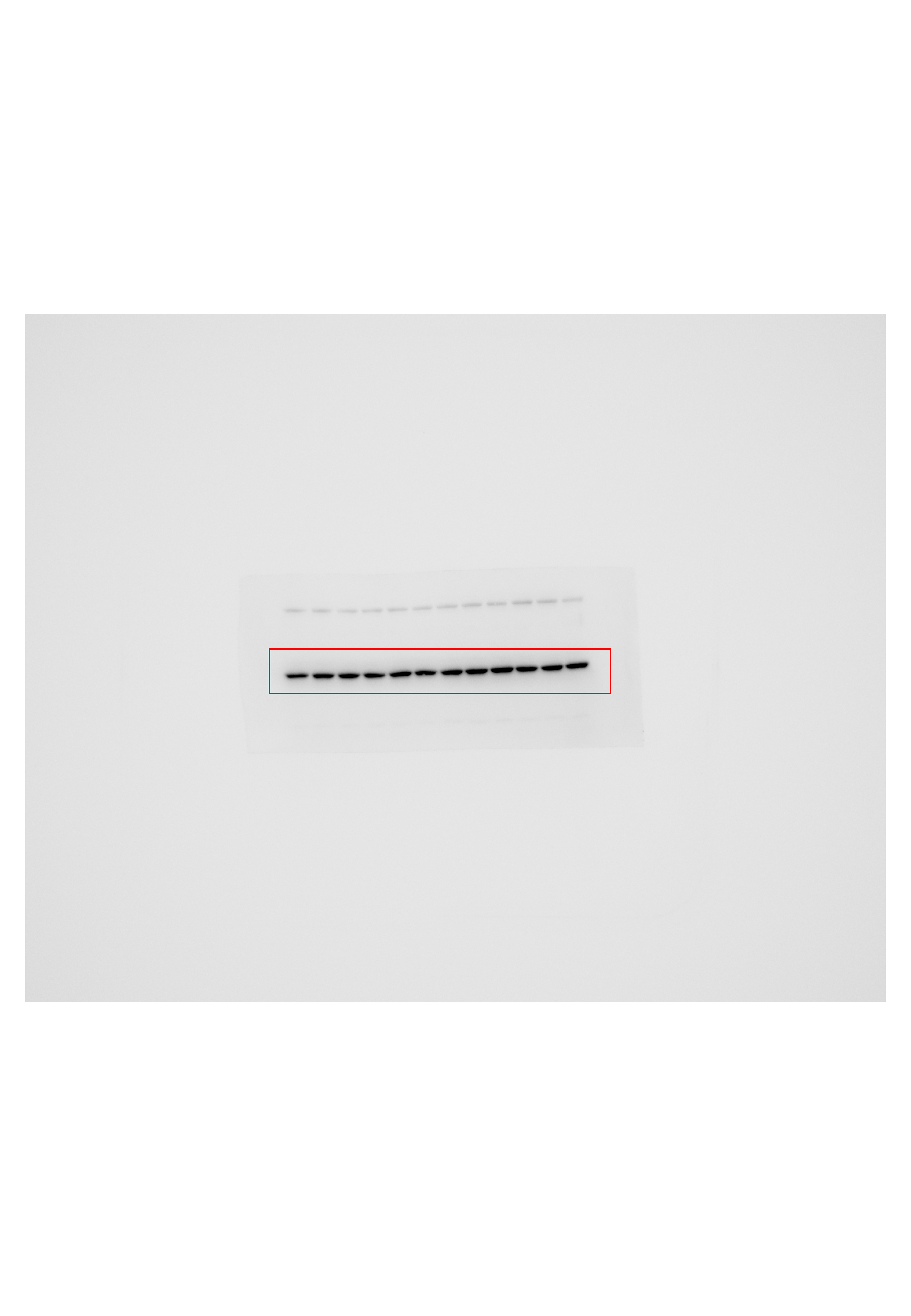

Supplement: Supplementary file 8 — Source data Fig. 3 [file 44318_2024_277_MOESM8_ESM.zip › SD figure 3/Figure 3A. anti-actin.tif]

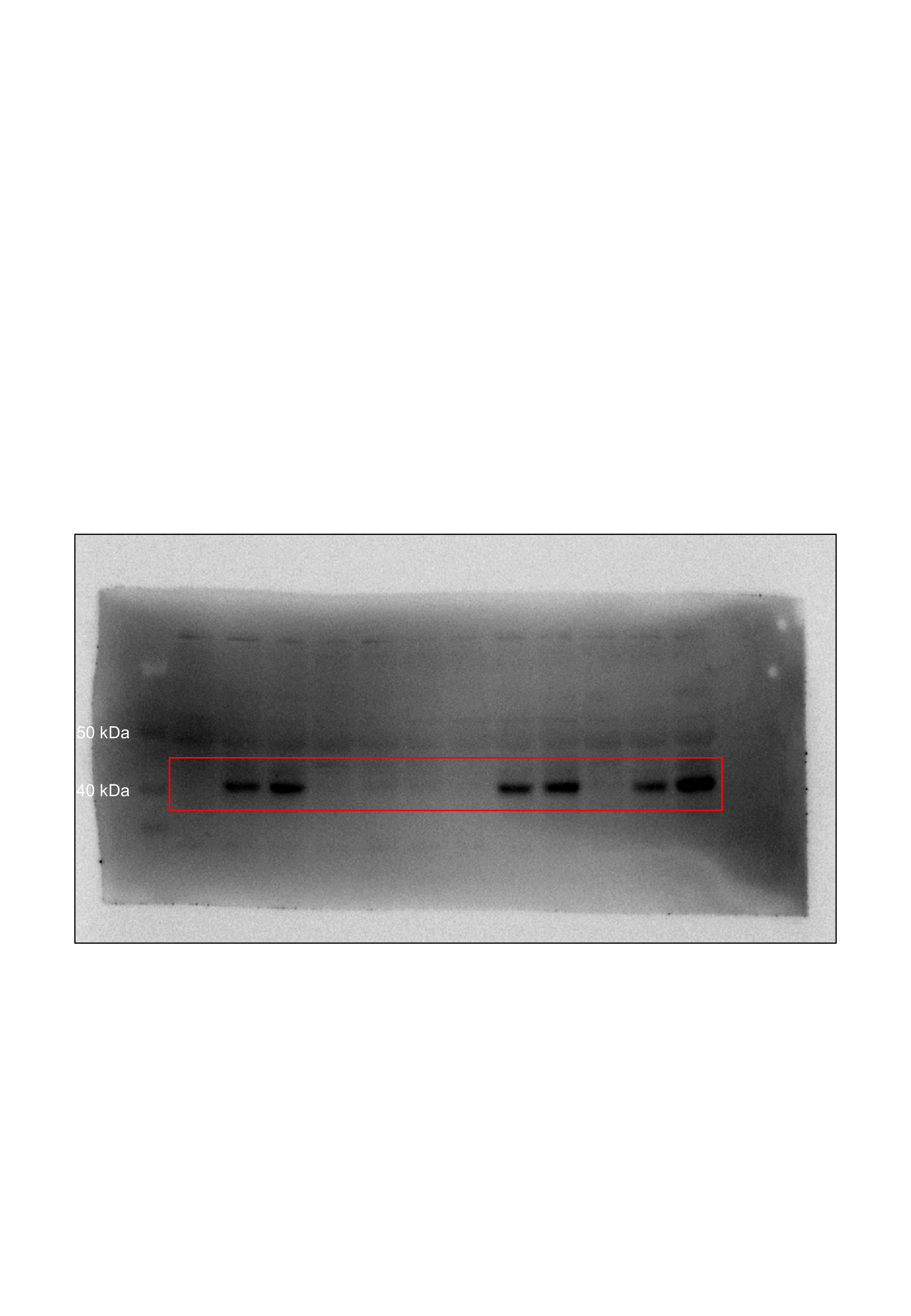

Supplement: Supplementary file 8 — Source data Fig. 3 [file 44318_2024_277_MOESM8_ESM.zip › SD figure 3/Figure 3A. anti-pS175-SnRK2s.tif]

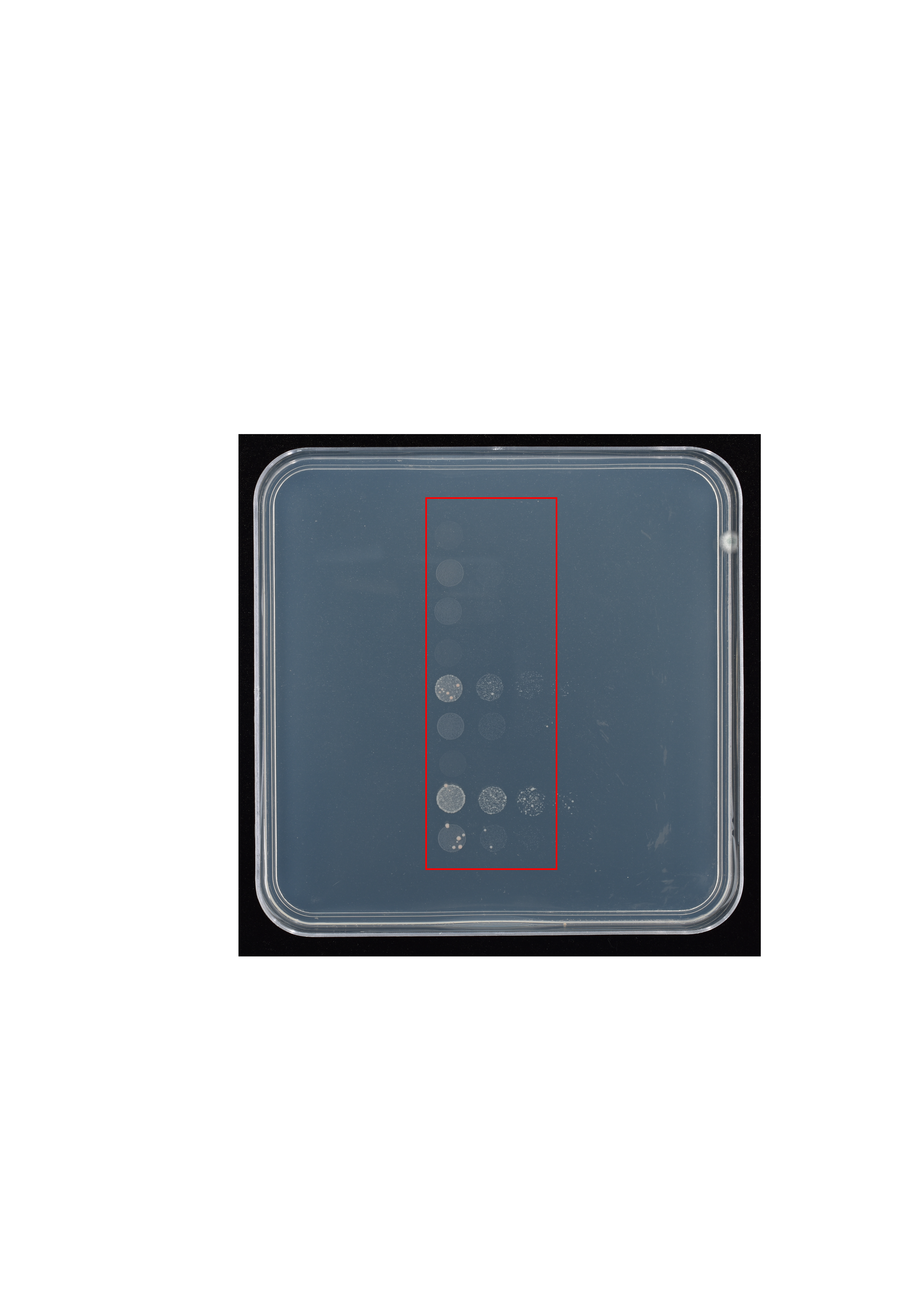

Supplement: Supplementary file 8 — Source data Fig. 3 [file 44318_2024_277_MOESM8_ESM.zip › SD figure 3/Figure 3C. -ALWMH, with 1 M man.tif]

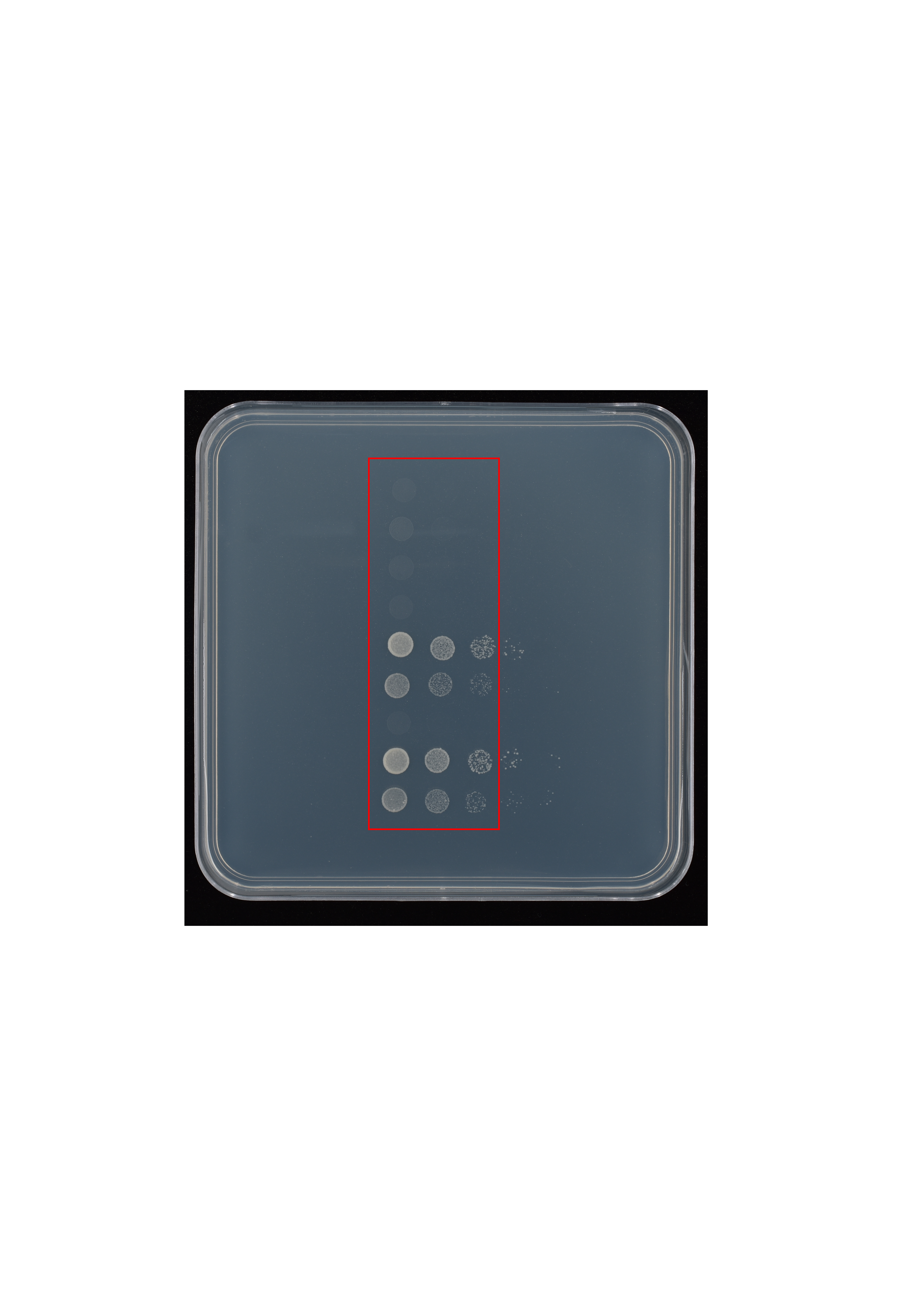

Supplement: Supplementary file 8 — Source data Fig. 3 [file 44318_2024_277_MOESM8_ESM.zip › SD figure 3/Figure 3C. -ALWMH.tif]

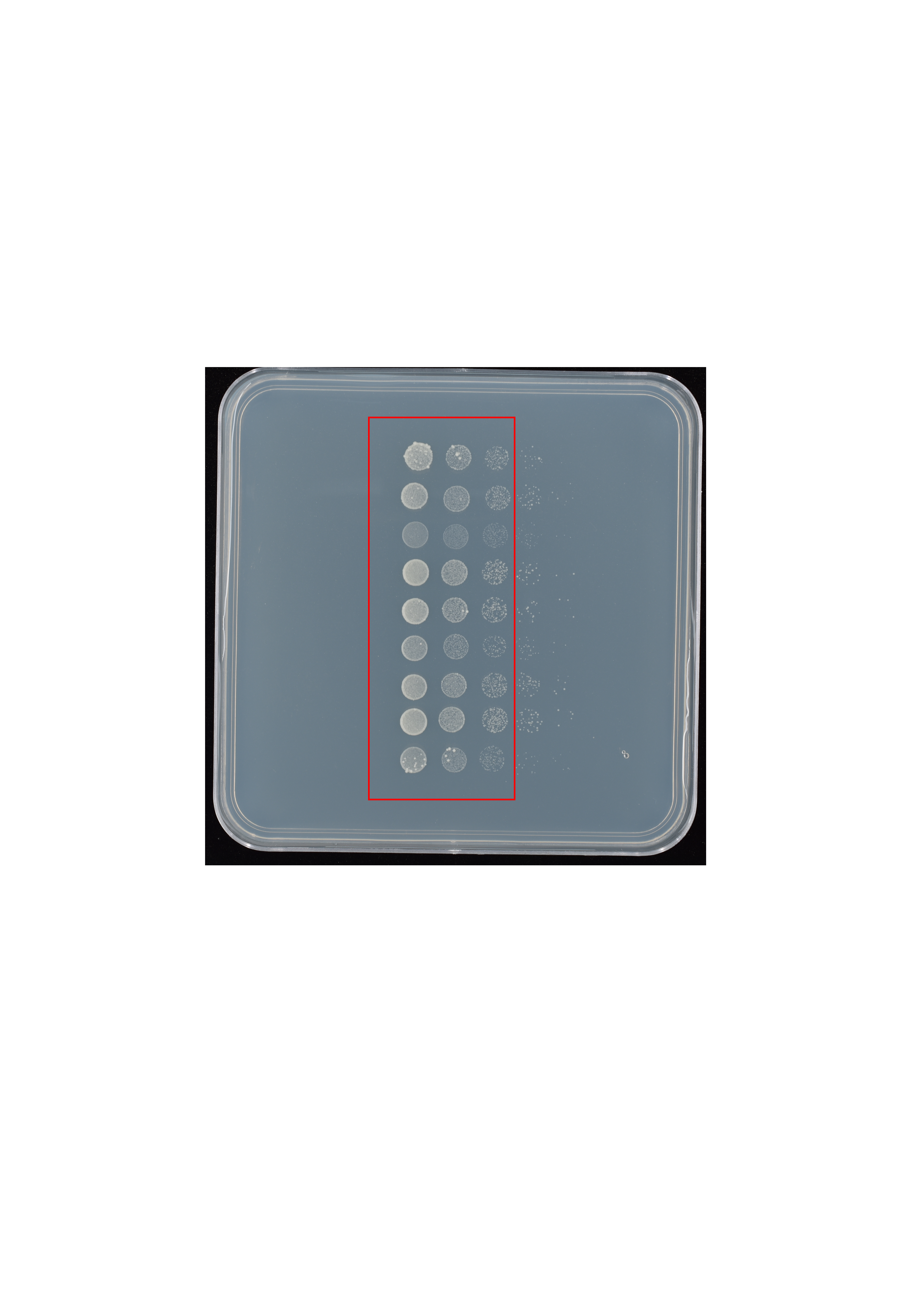

Supplement: Supplementary file 8 — Source data Fig. 3 [file 44318_2024_277_MOESM8_ESM.zip › SD figure 3/Figure 3C. -LWM.tif]

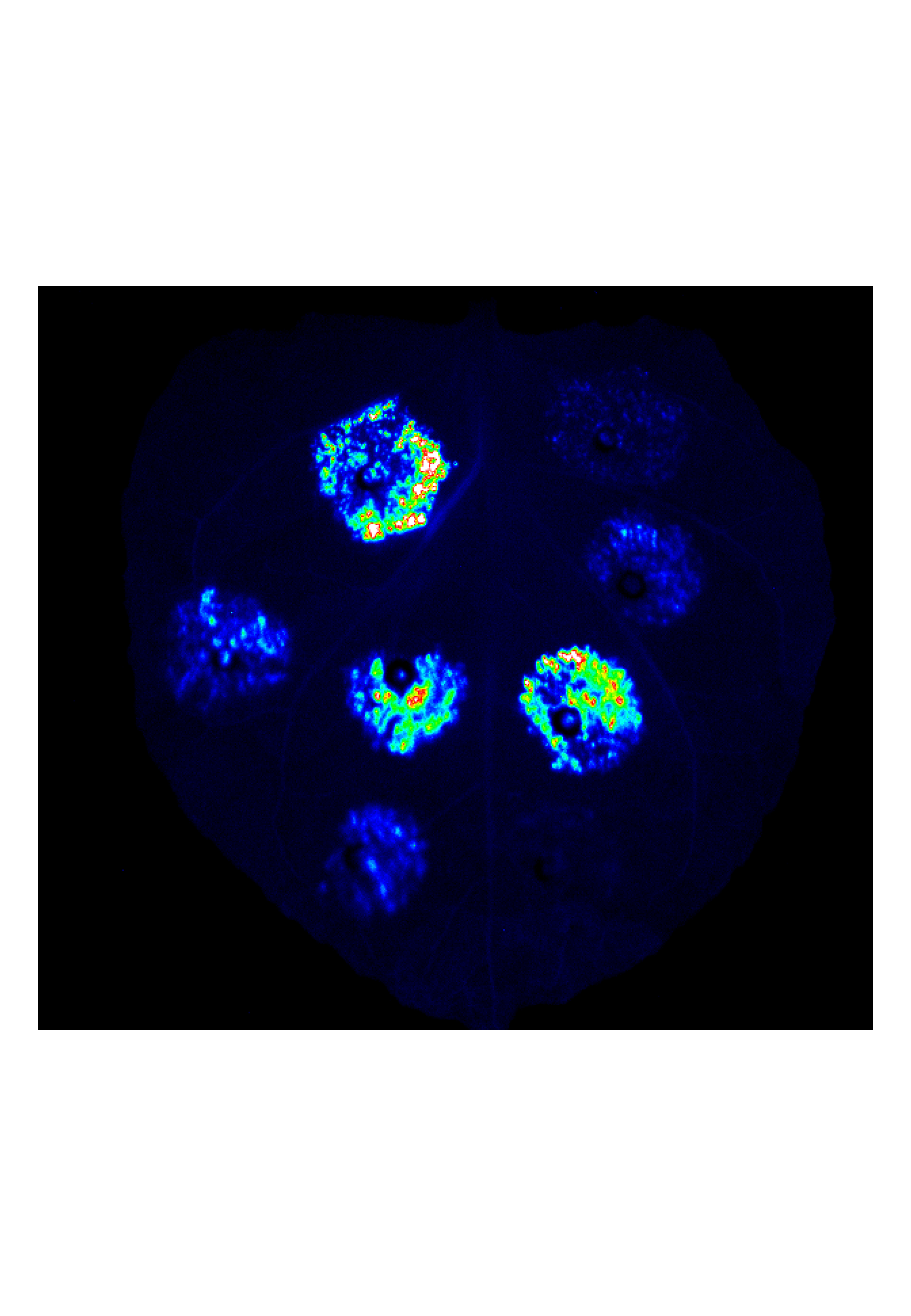

Supplement: Supplementary file 8 — Source data Fig. 3 [file 44318_2024_277_MOESM8_ESM.zip › SD figure 3/Figure 3D. split LUC.tif]

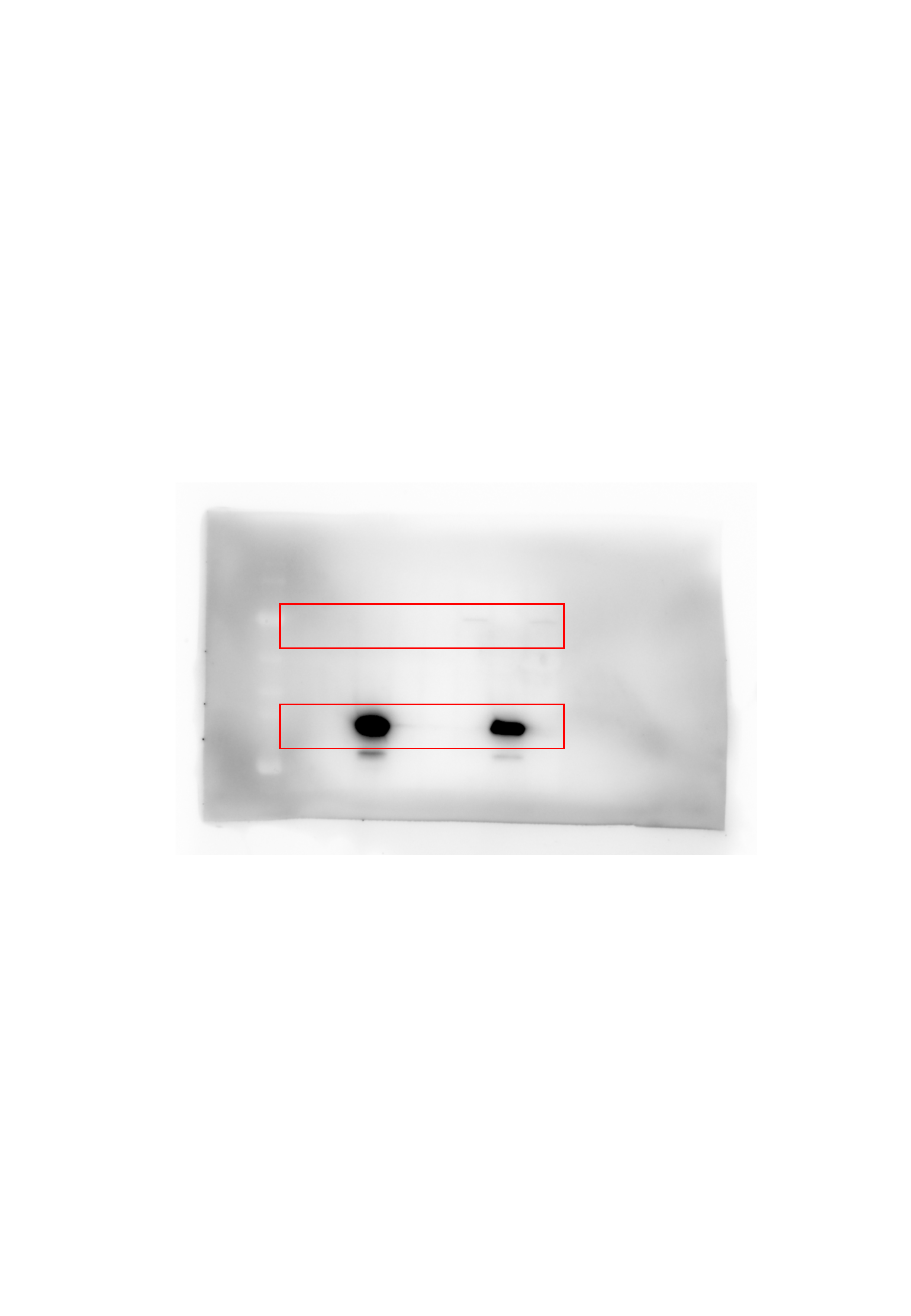

Supplement: Supplementary file 8 — Source data Fig. 3 [file 44318_2024_277_MOESM8_ESM.zip › SD figure 3/Figure 3E. anti-GFP.tif]

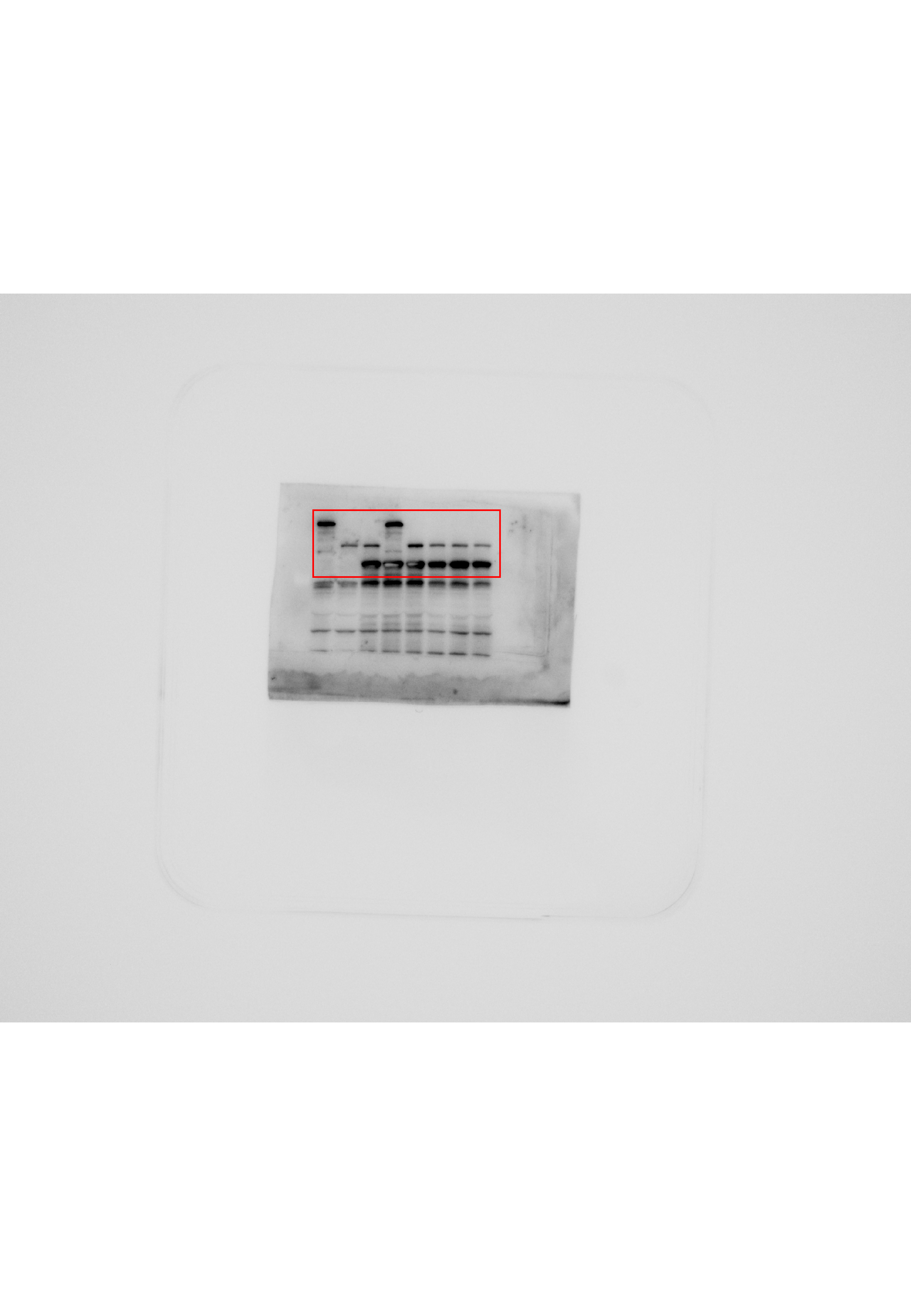

Supplement: Supplementary file 8 — Source data Fig. 3 [file 44318_2024_277_MOESM8_ESM.zip › SD figure 3/Figure 3E. anti-LUC.tif]

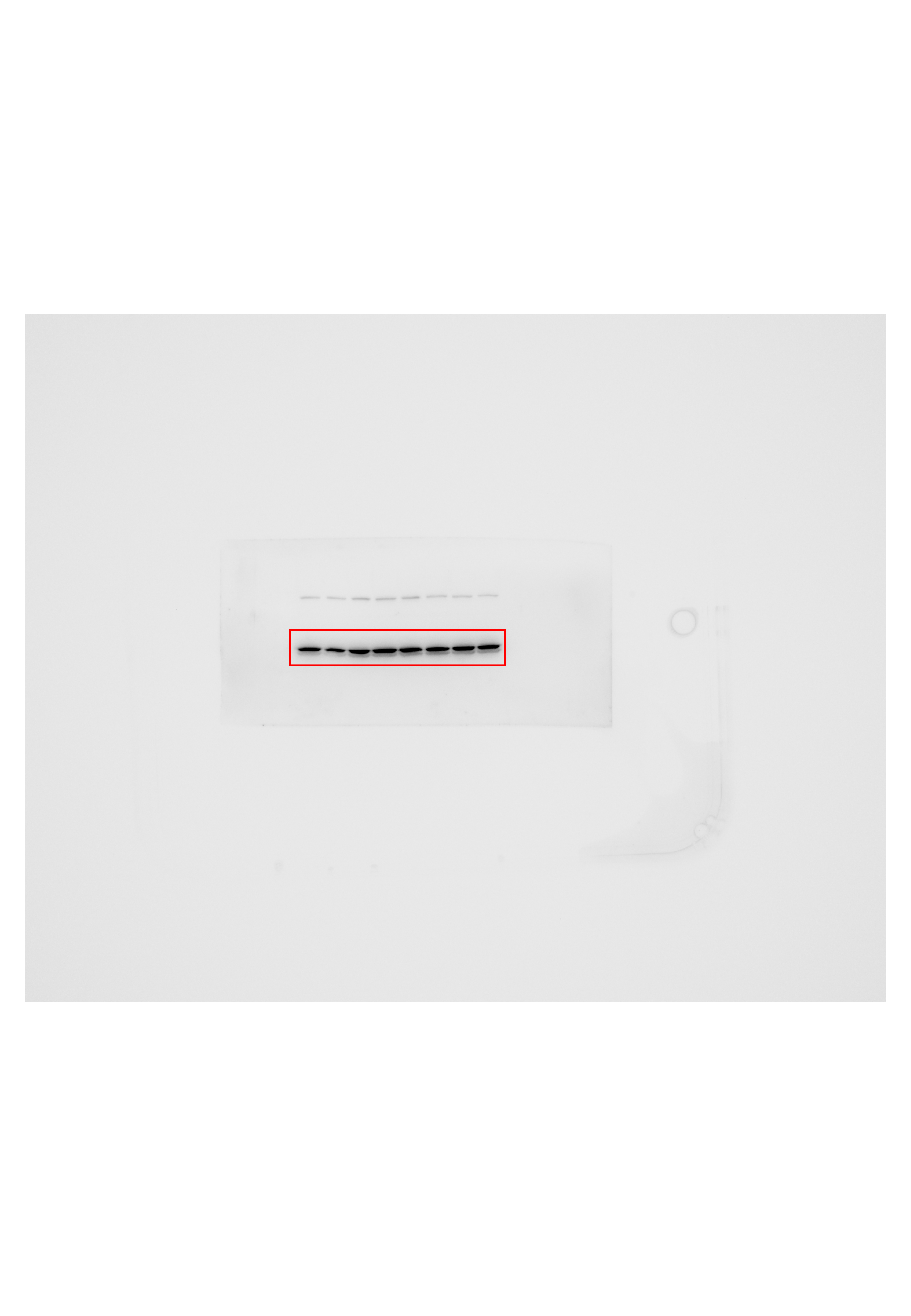

Supplement: Supplementary file 8 — Source data Fig. 3 [file 44318_2024_277_MOESM8_ESM.zip › SD figure 3/Figure 3E. anti-actin.tif]

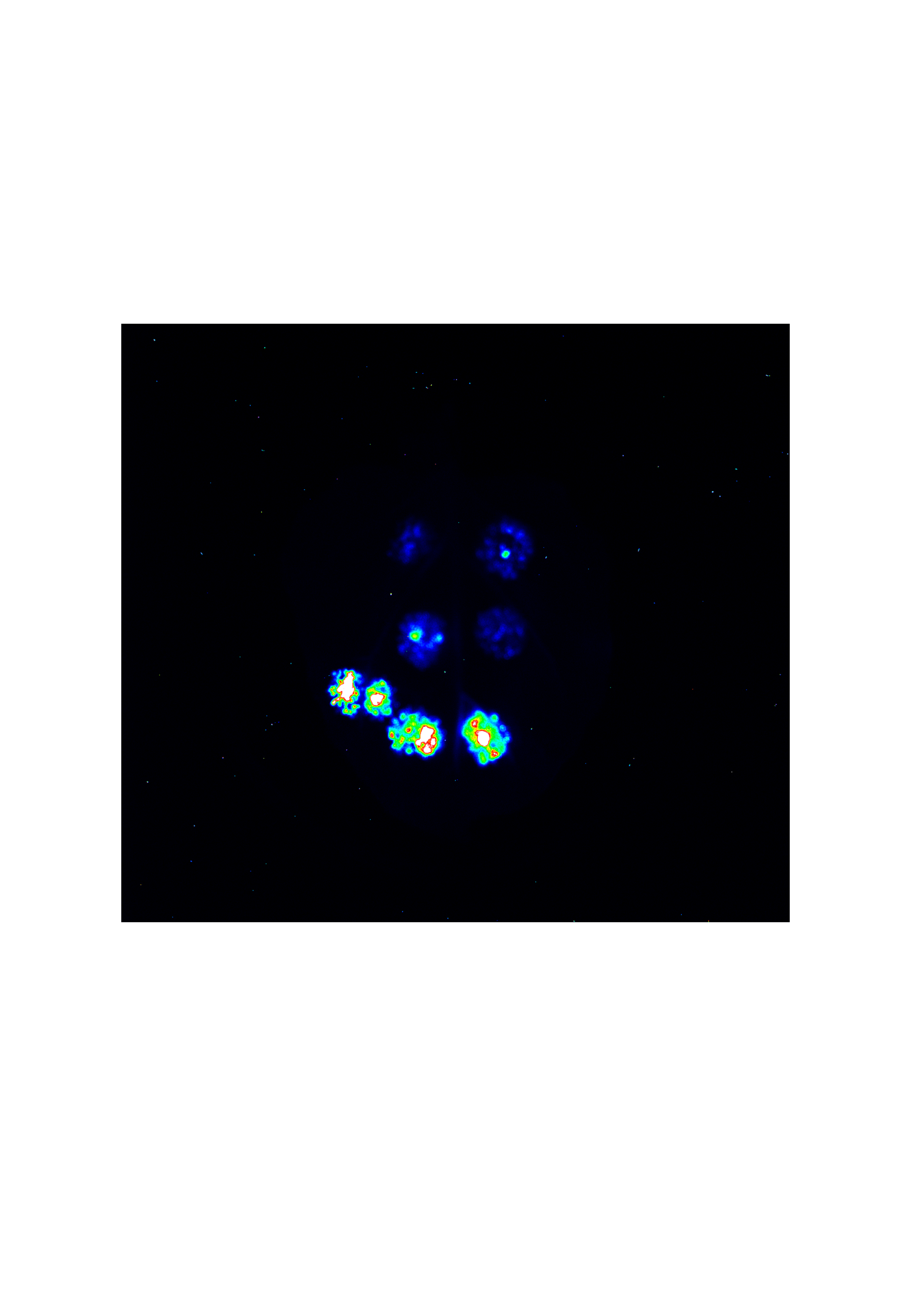

Supplement: Supplementary file 8 — Source data Fig. 3 [file 44318_2024_277_MOESM8_ESM.zip › SD figure 3/Figure 3F. split LUC.tif]

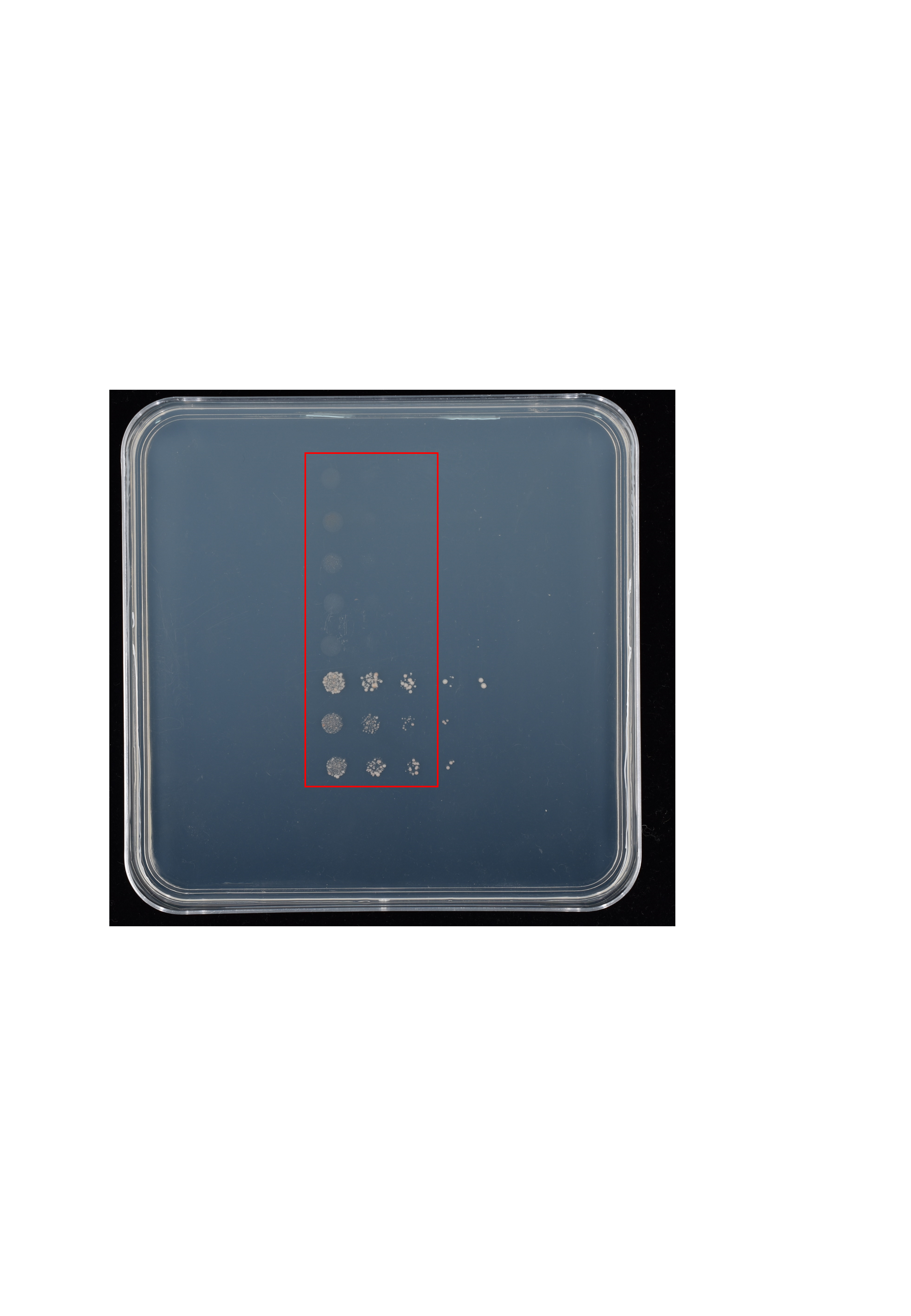

Supplement: Supplementary file 8 — Source data Fig. 3 [file 44318_2024_277_MOESM8_ESM.zip › SD figure 3/Figure 3G. -ALWMH, with 1 M man.tif]

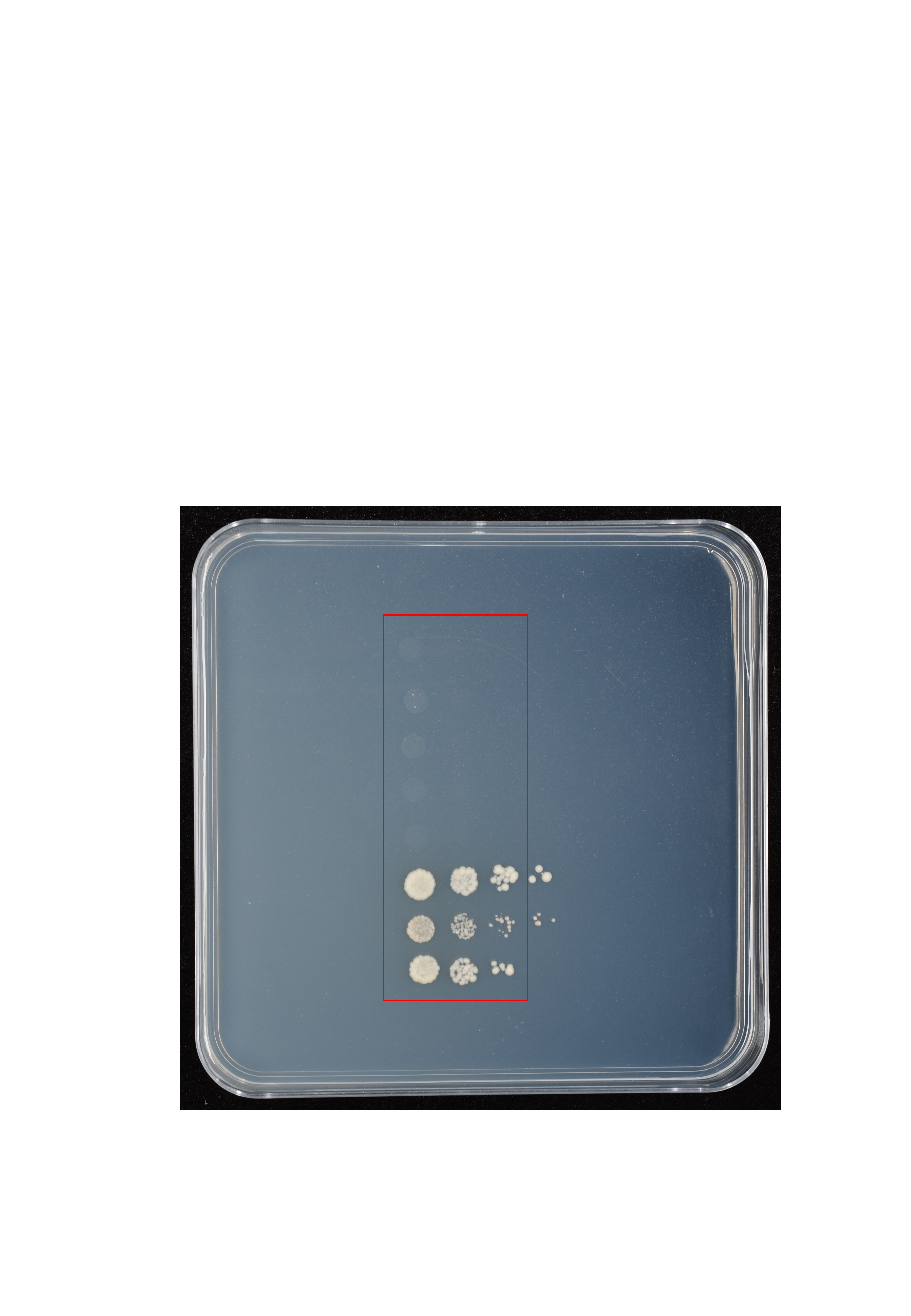

Supplement: Supplementary file 8 — Source data Fig. 3 [file 44318_2024_277_MOESM8_ESM.zip › SD figure 3/Figure 3G. -ALWMH.tif]

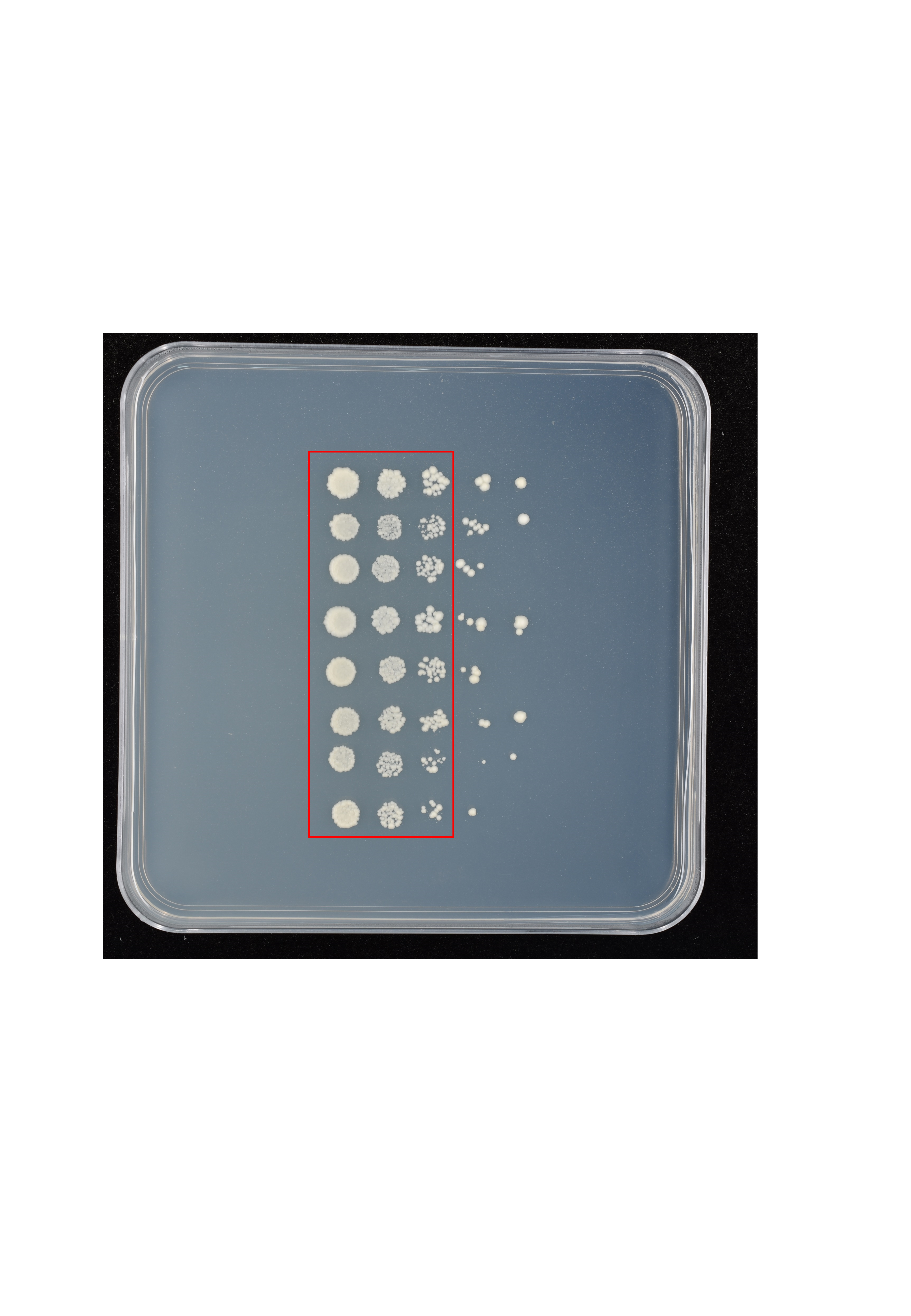

Supplement: Supplementary file 8 — Source data Fig. 3 [file 44318_2024_277_MOESM8_ESM.zip › SD figure 3/Figure 3G. -LWM.tif]

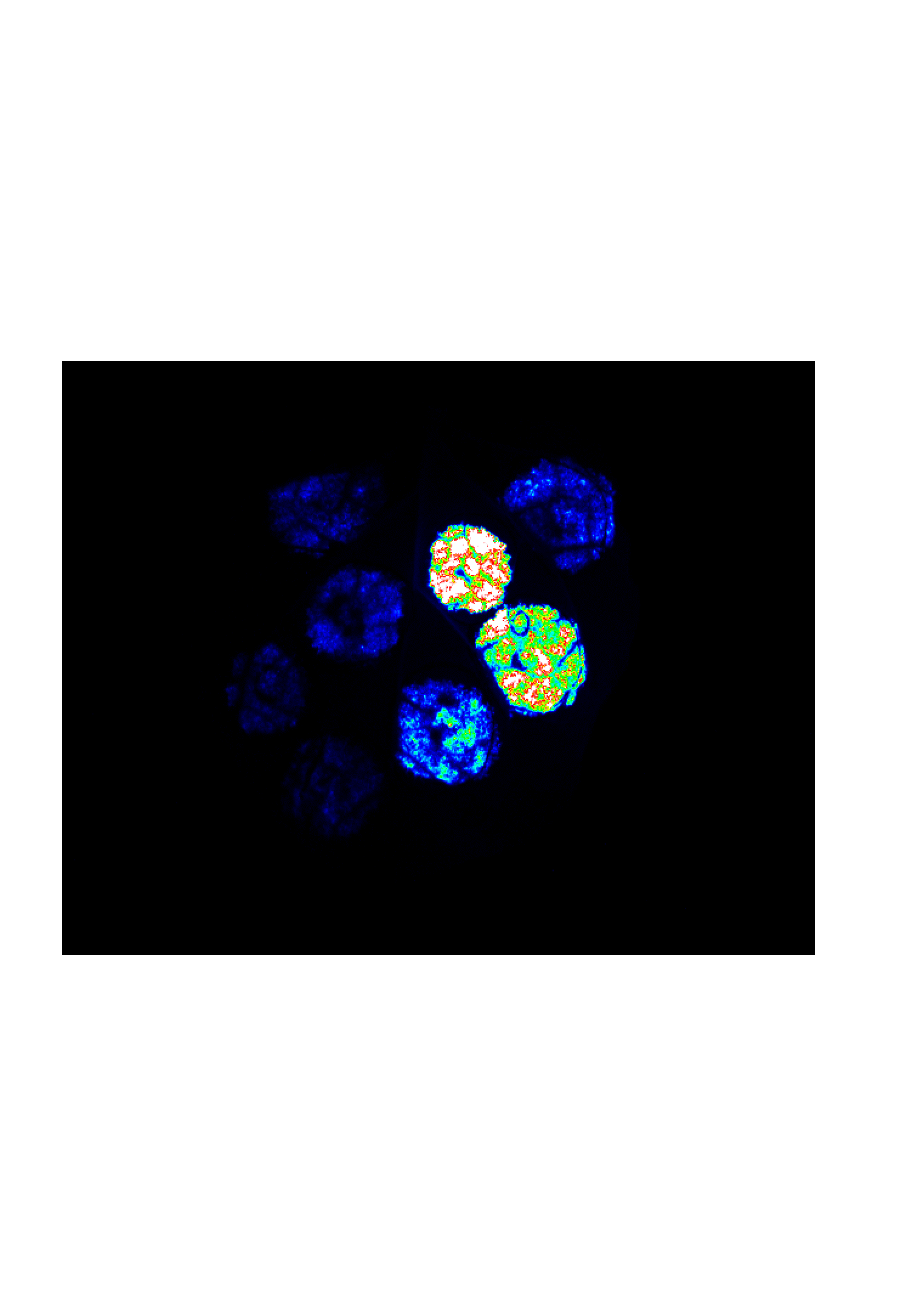

Supplement: Supplementary file 9 — Source data Fig. 4 [file 44318_2024_277_MOESM9_ESM.zip › SD figure 4/Figure 4A.tif]

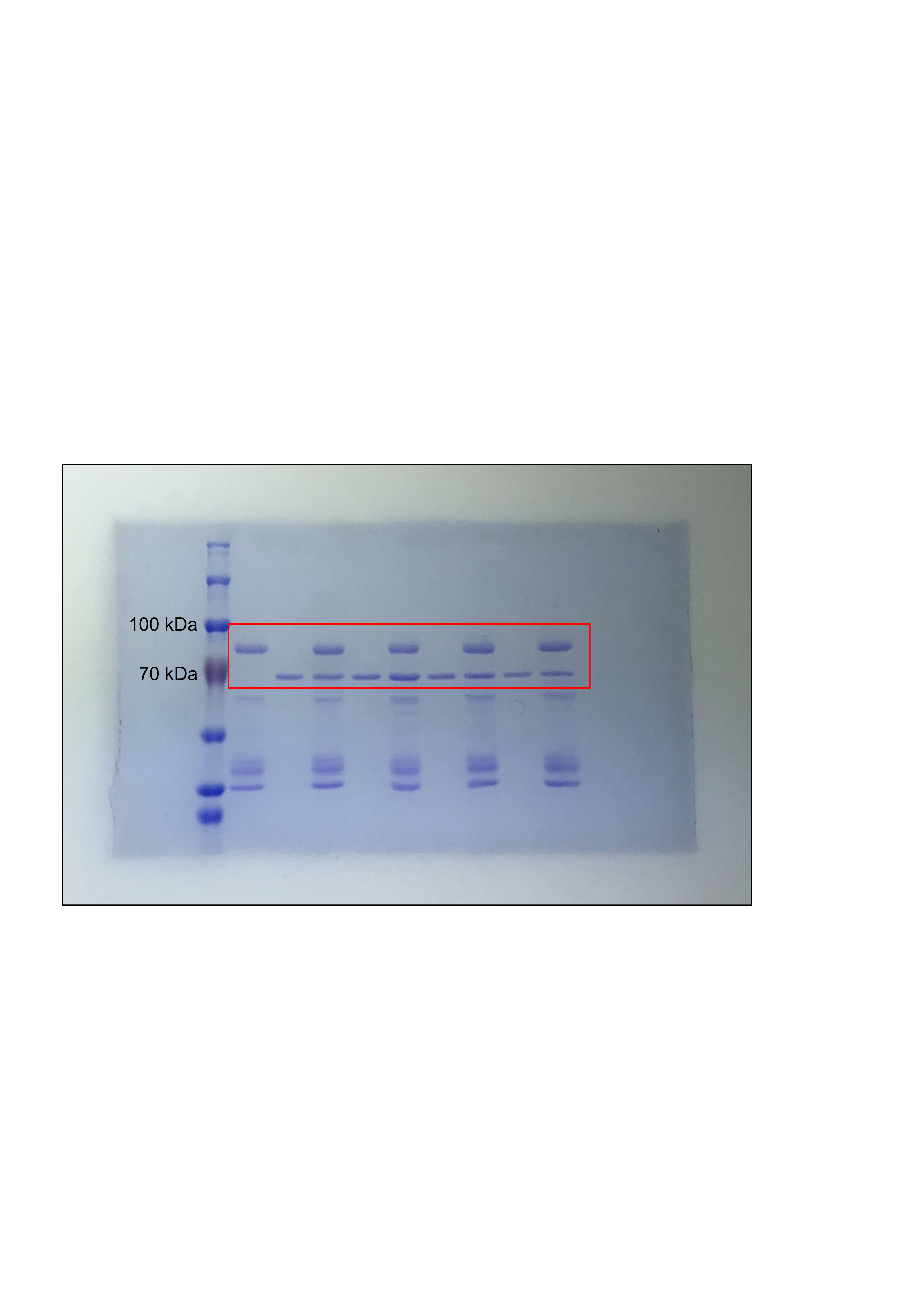

Supplement: Supplementary file 9 — Source data Fig. 4 [file 44318_2024_277_MOESM9_ESM.zip › SD figure 4/Figure 4B. CBB.tif]

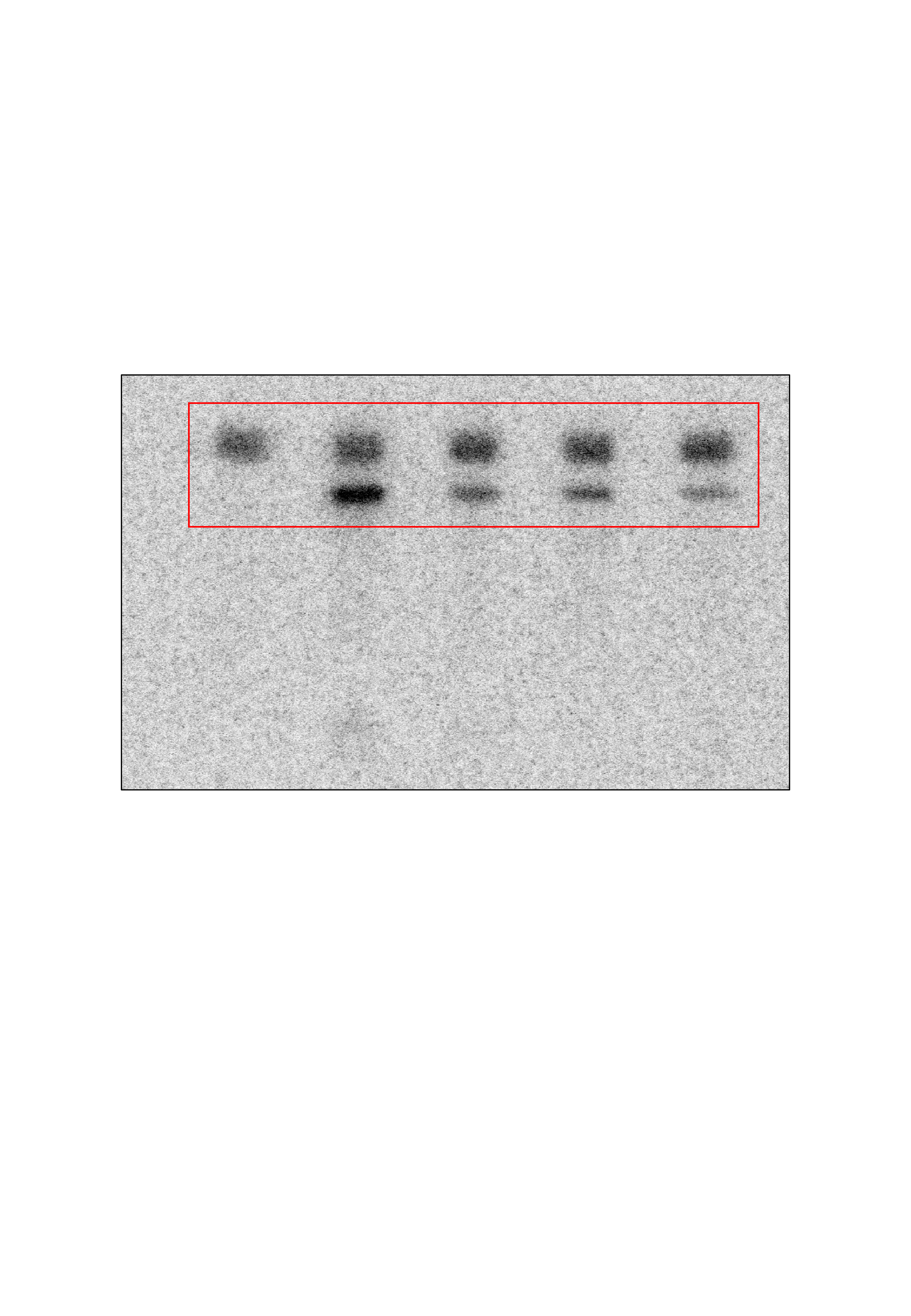

Supplement: Supplementary file 9 — Source data Fig. 4 [file 44318_2024_277_MOESM9_ESM.zip › SD figure 4/Figure 4B. auto-radiograph.tif]

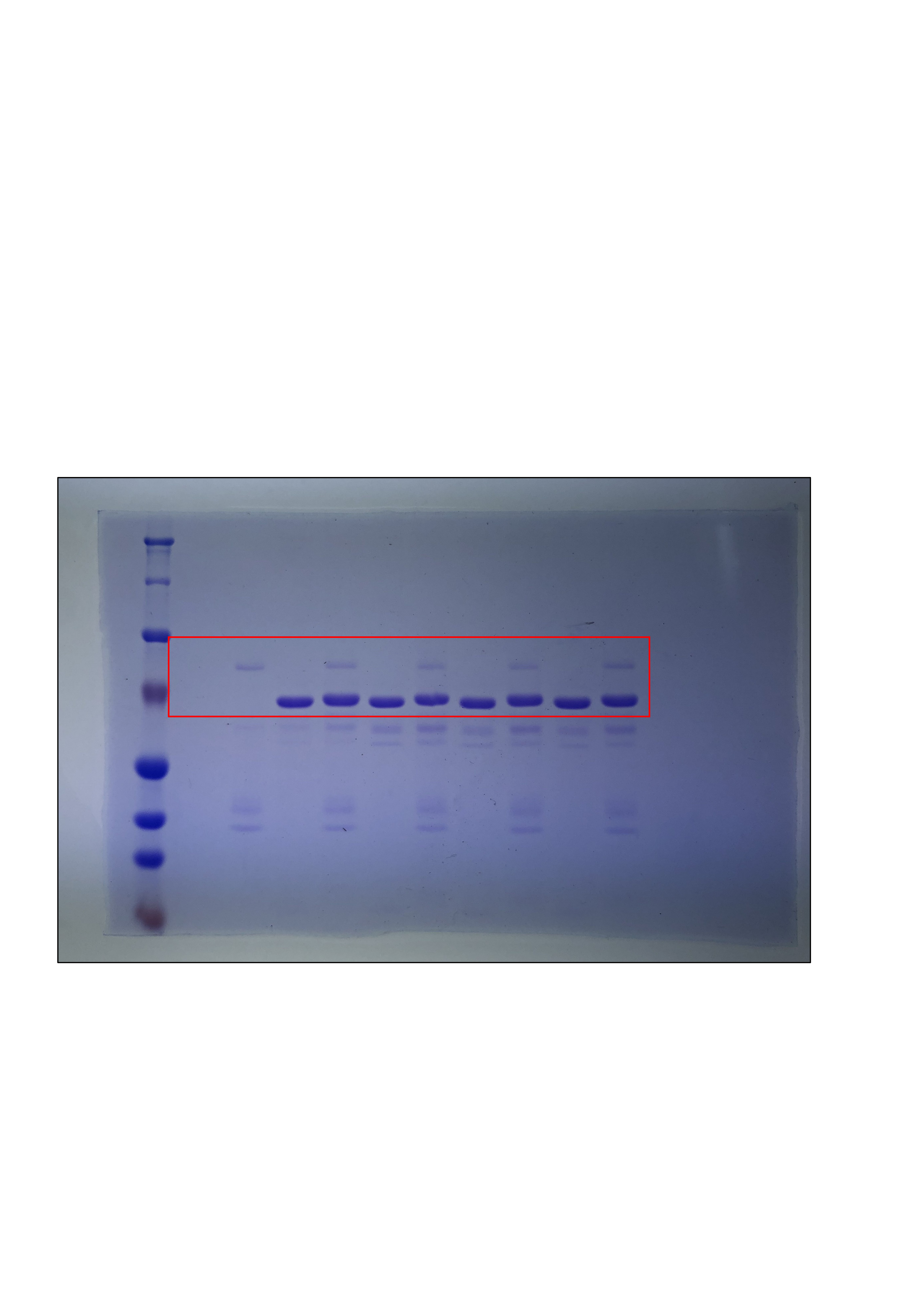

Supplement: Supplementary file 9 — Source data Fig. 4 [file 44318_2024_277_MOESM9_ESM.zip › SD figure 4/Figure 4C. CBB.tif]

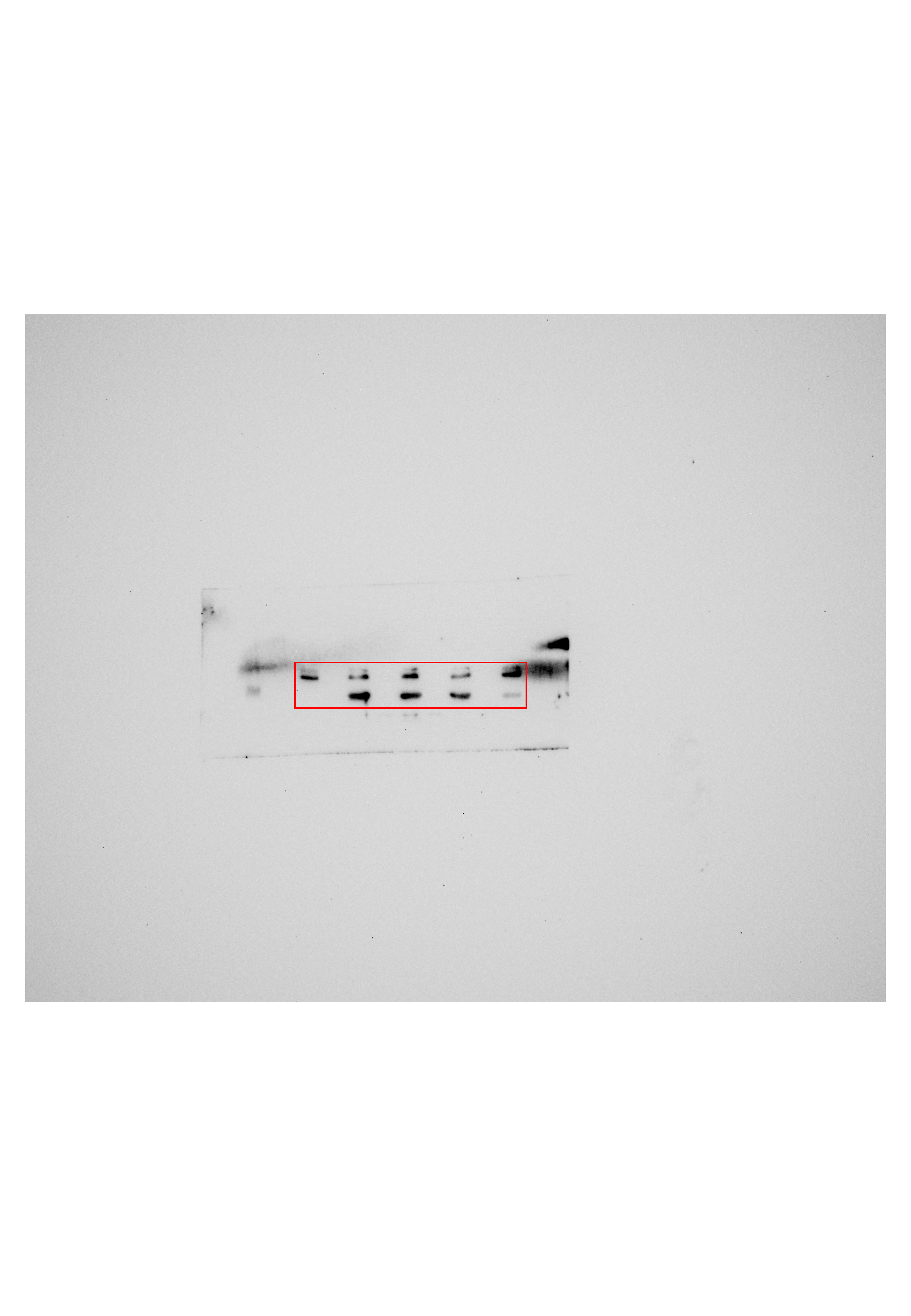

Supplement: Supplementary file 9 — Source data Fig. 4 [file 44318_2024_277_MOESM9_ESM.zip › SD figure 4/Figure 4C. anti-phos-tyrosine.tif]

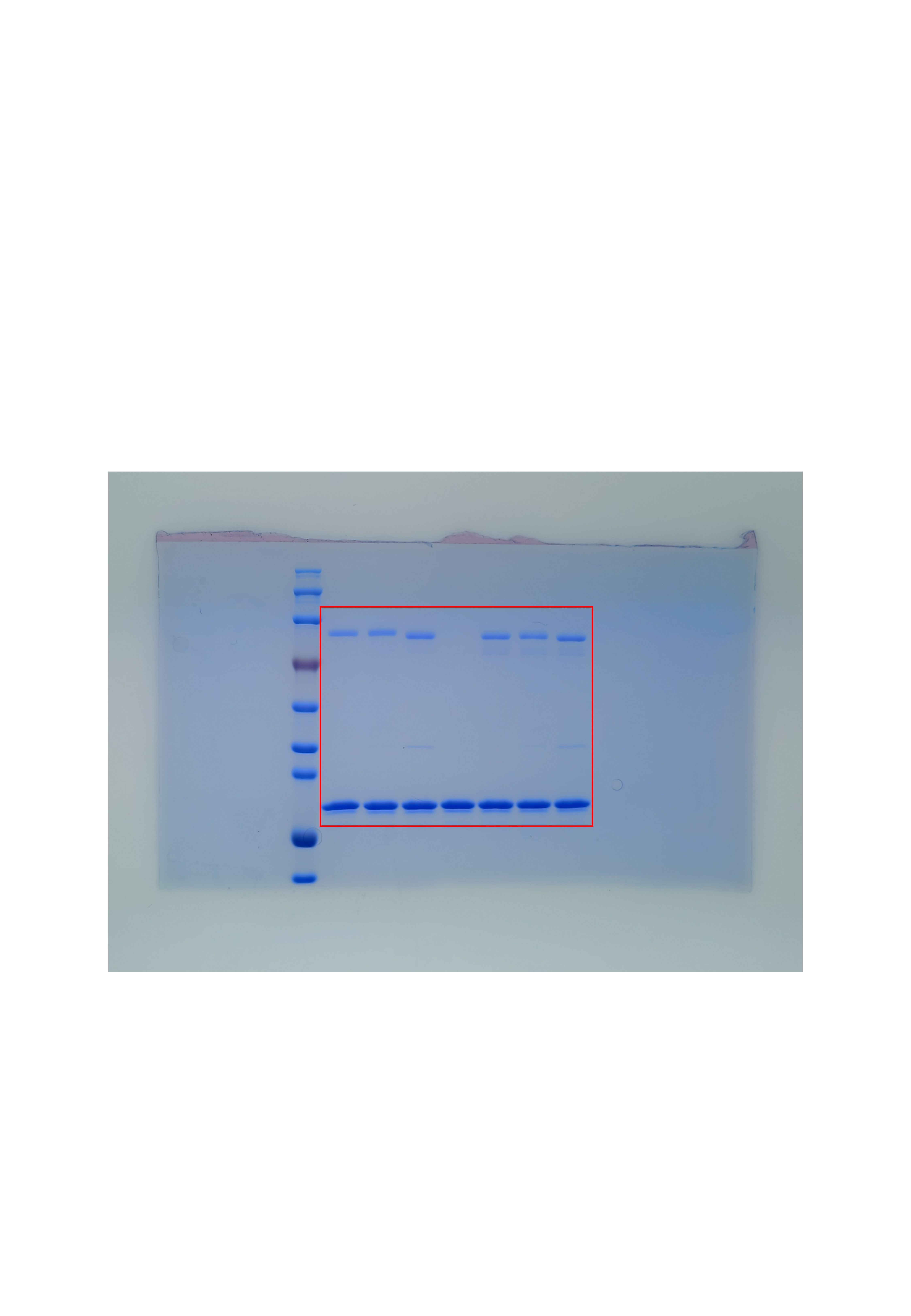

Supplement: Supplementary file 9 — Source data Fig. 4 [file 44318_2024_277_MOESM9_ESM.zip › SD figure 4/Figure 4H. CBB.tif]

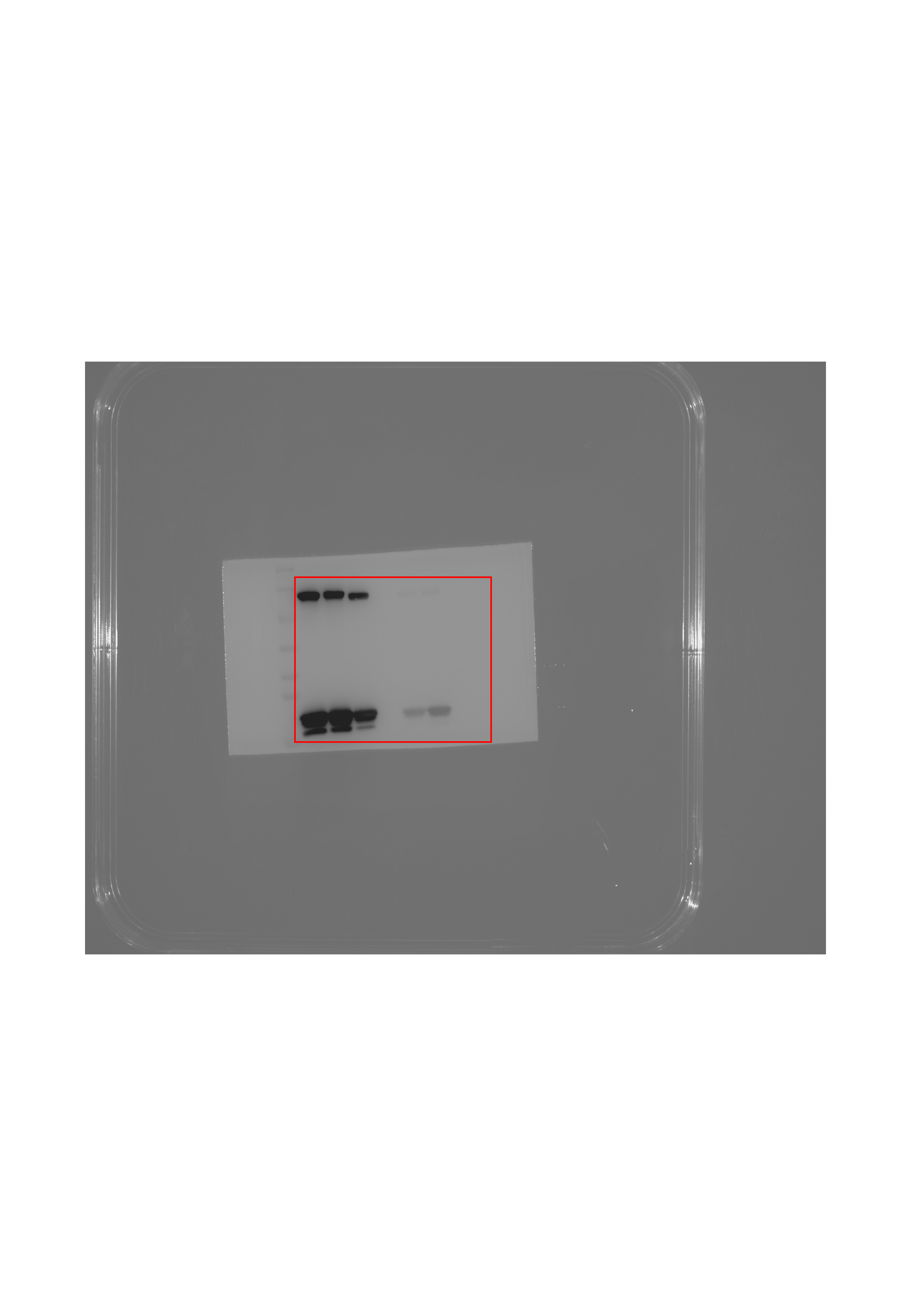

Supplement: Supplementary file 9 — Source data Fig. 4 [file 44318_2024_277_MOESM9_ESM.zip › SD figure 4/Figure 4H. anti-thiophosphate ester.tif]

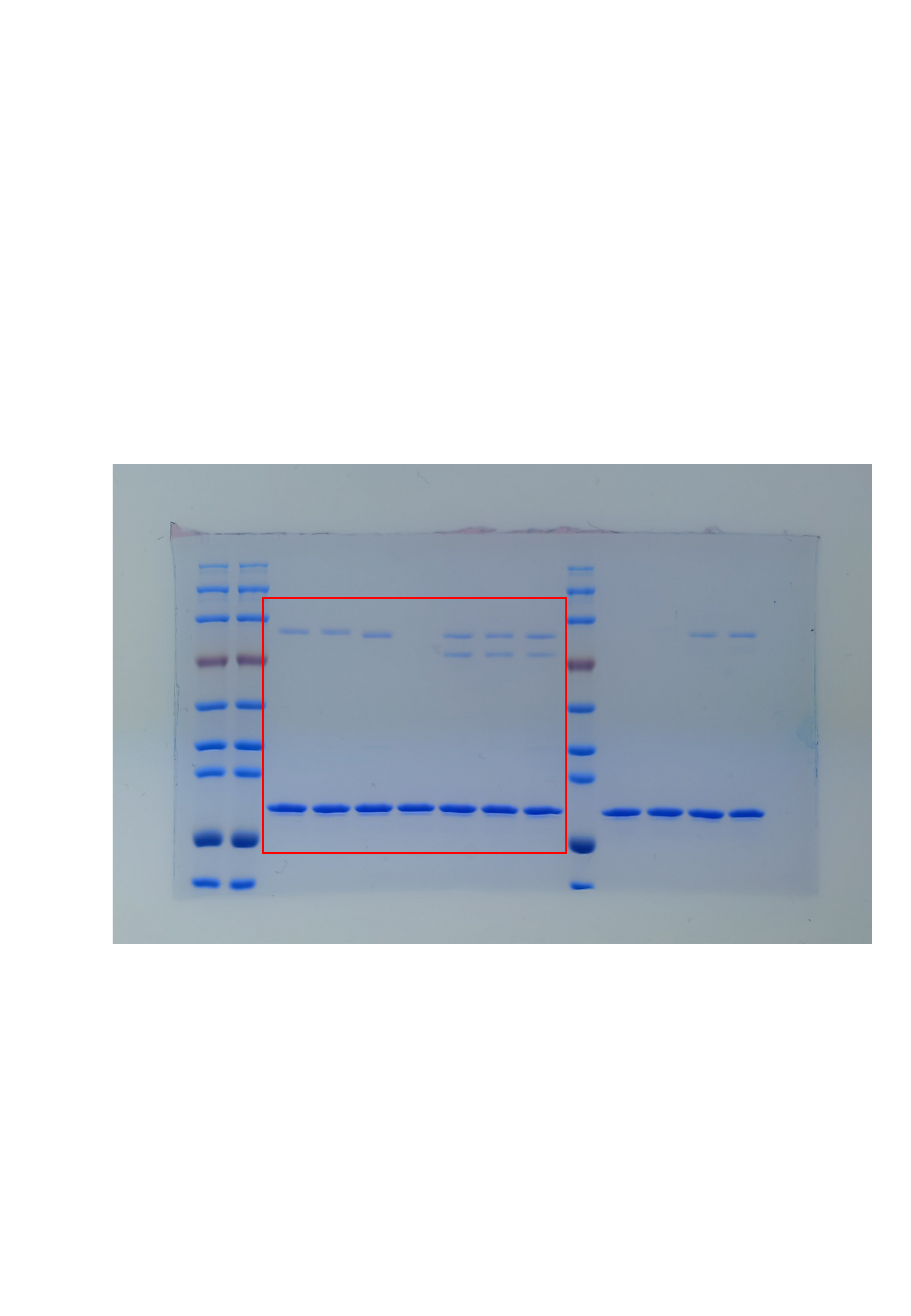

Supplement: Supplementary file 9 — Source data Fig. 4 [file 44318_2024_277_MOESM9_ESM.zip › SD figure 4/Figure 4I. CBB.tif]

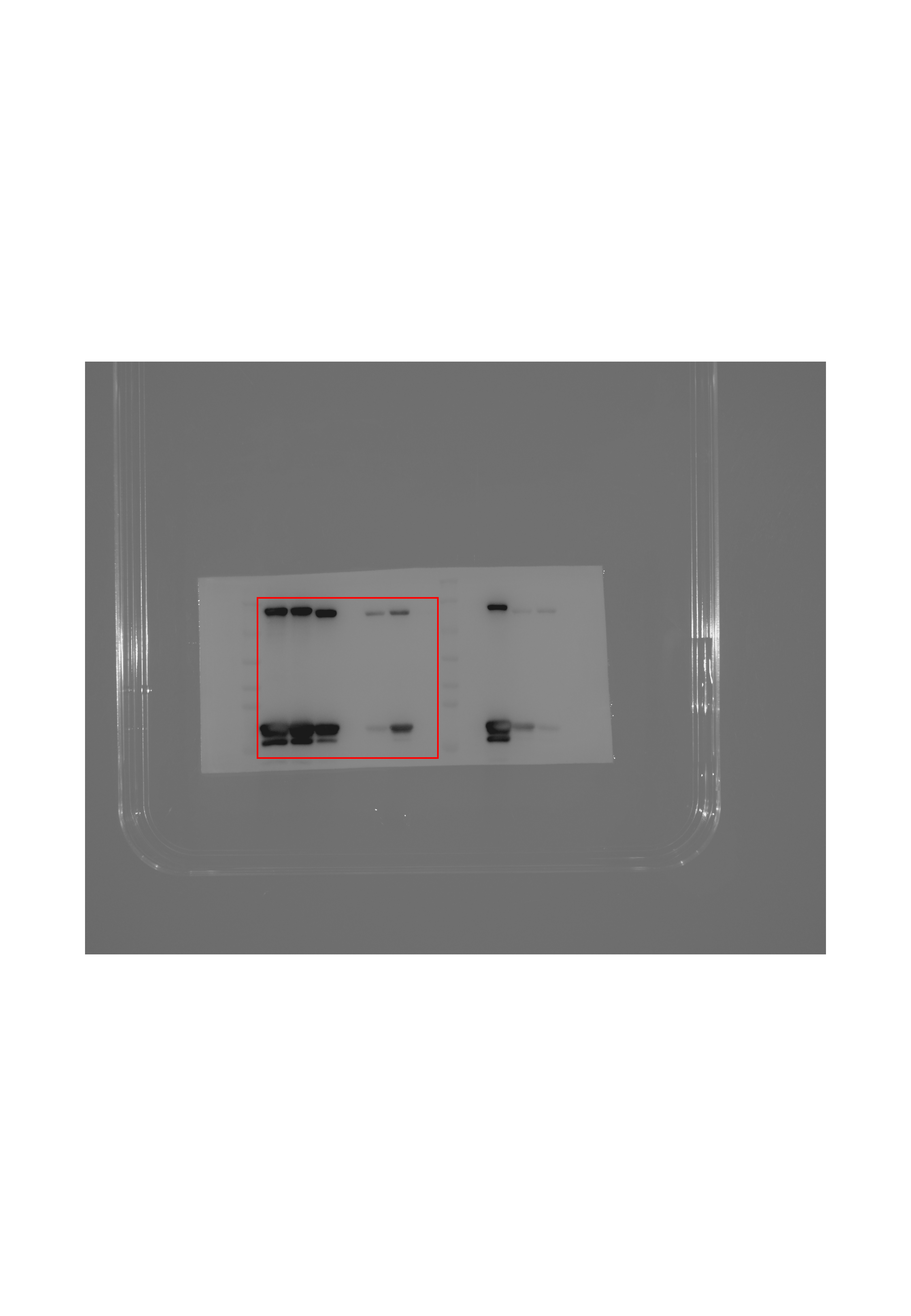

Supplement: Supplementary file 9 — Source data Fig. 4 [file 44318_2024_277_MOESM9_ESM.zip › SD figure 4/Figure 4I. anti-thiophosphate ester.tif]

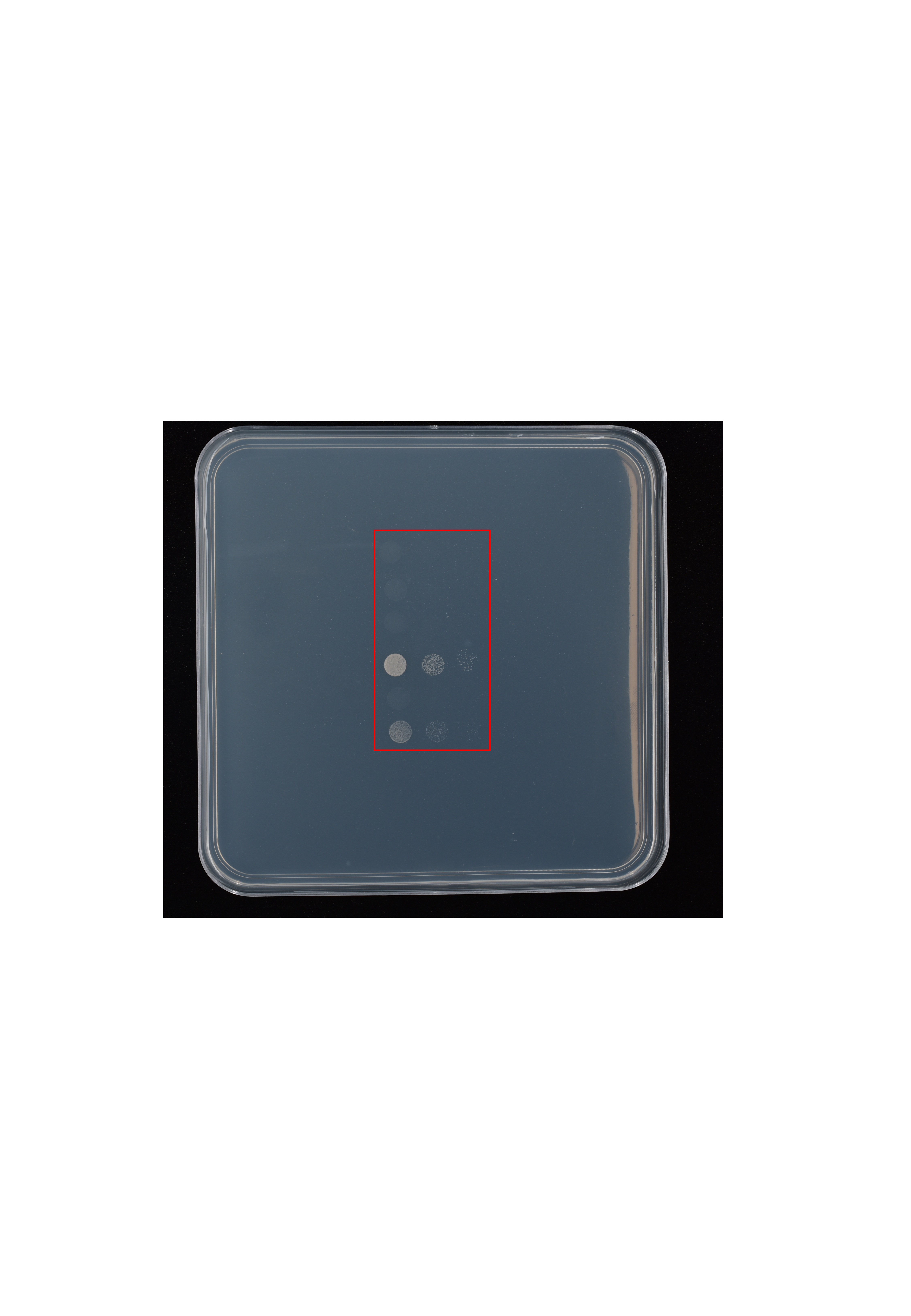

Supplement: Supplementary file 10 — Source data Fig. 5 [file 44318_2024_277_MOESM10_ESM.zip › SD figure 5/Figure 5A. -ALWH.tif]

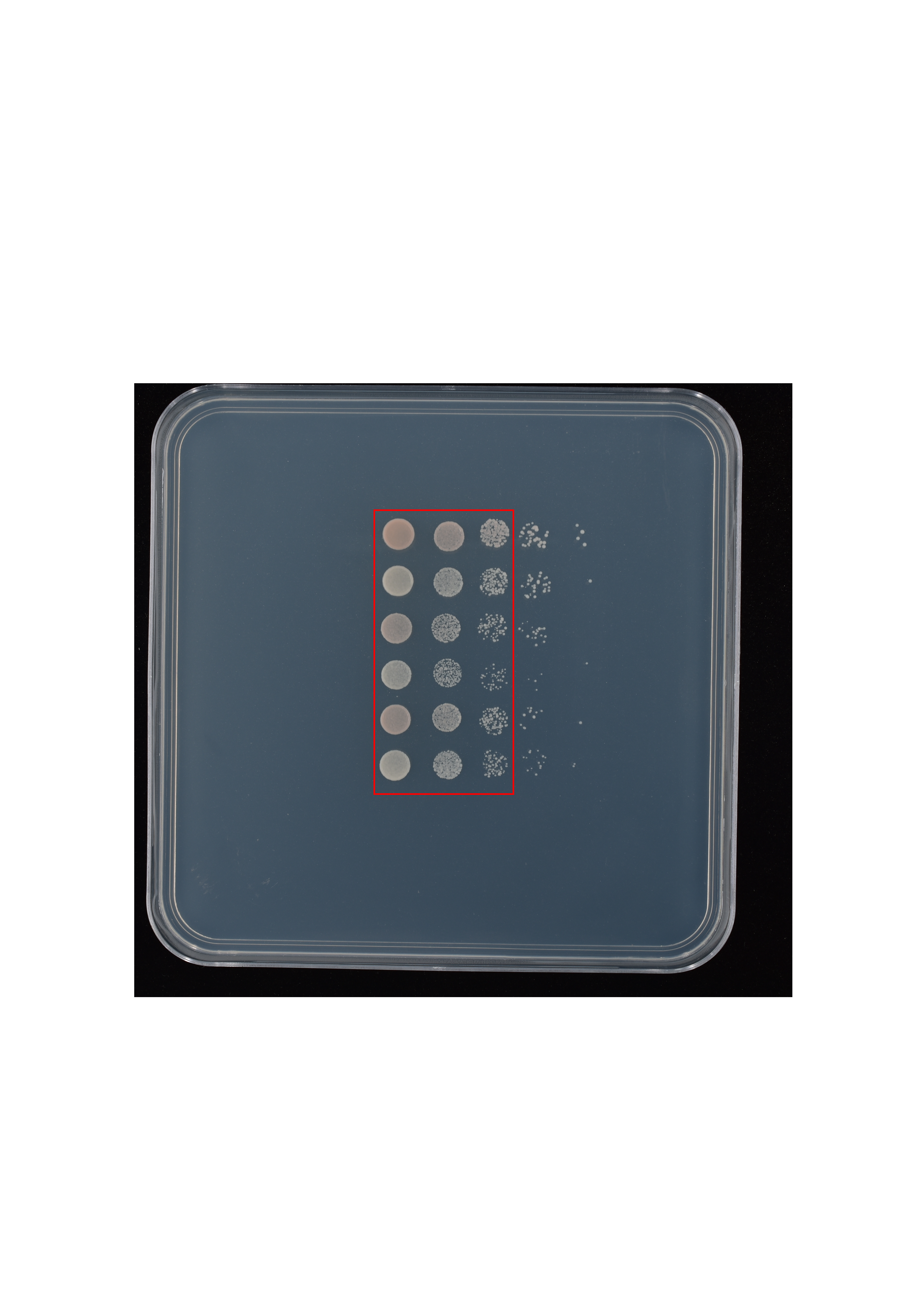

Supplement: Supplementary file 10 — Source data Fig. 5 [file 44318_2024_277_MOESM10_ESM.zip › SD figure 5/Figure 5A. -LW.tif]

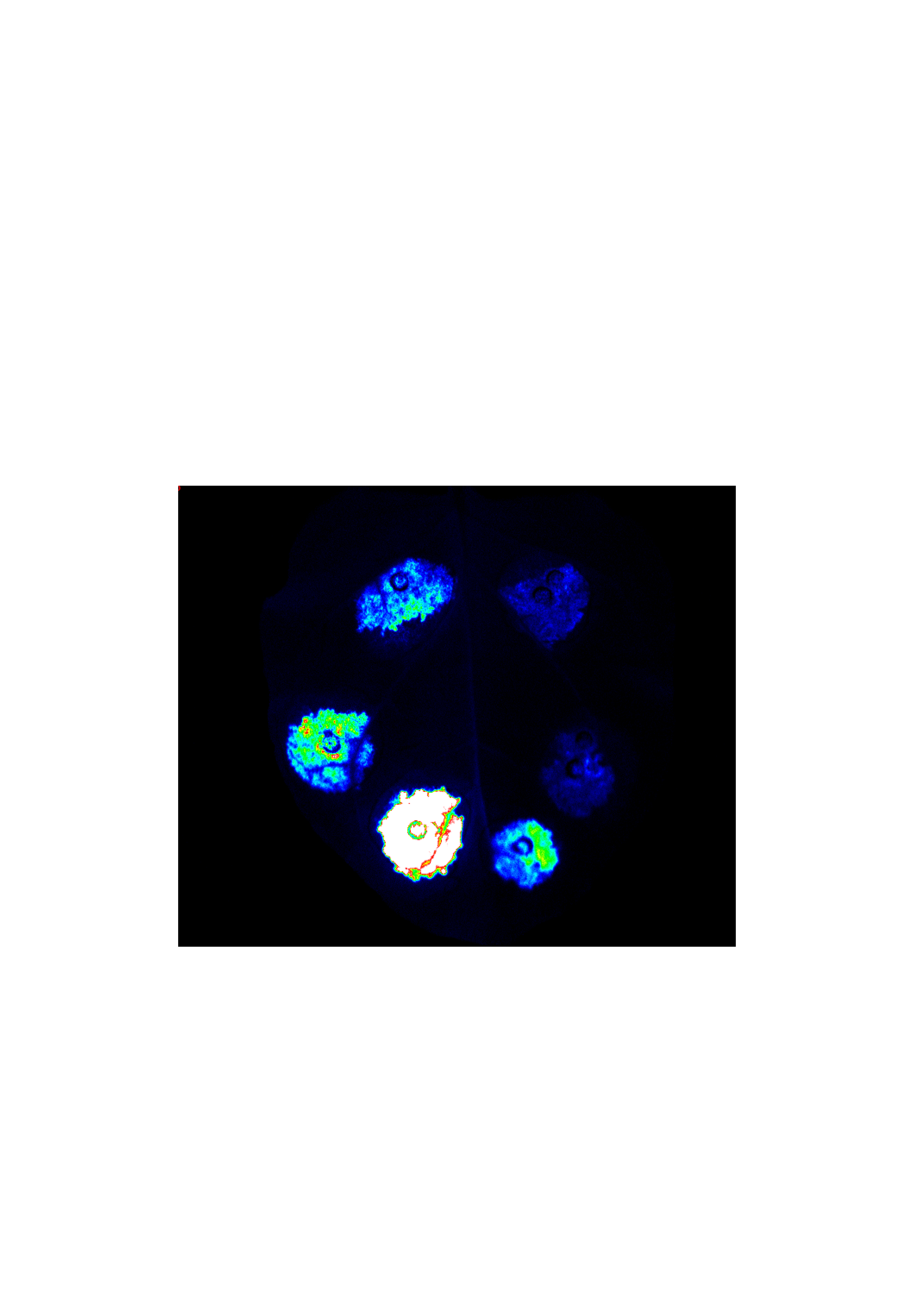

Supplement: Supplementary file 10 — Source data Fig. 5 [file 44318_2024_277_MOESM10_ESM.zip › SD figure 5/Figure 5B. split LUC.tif]

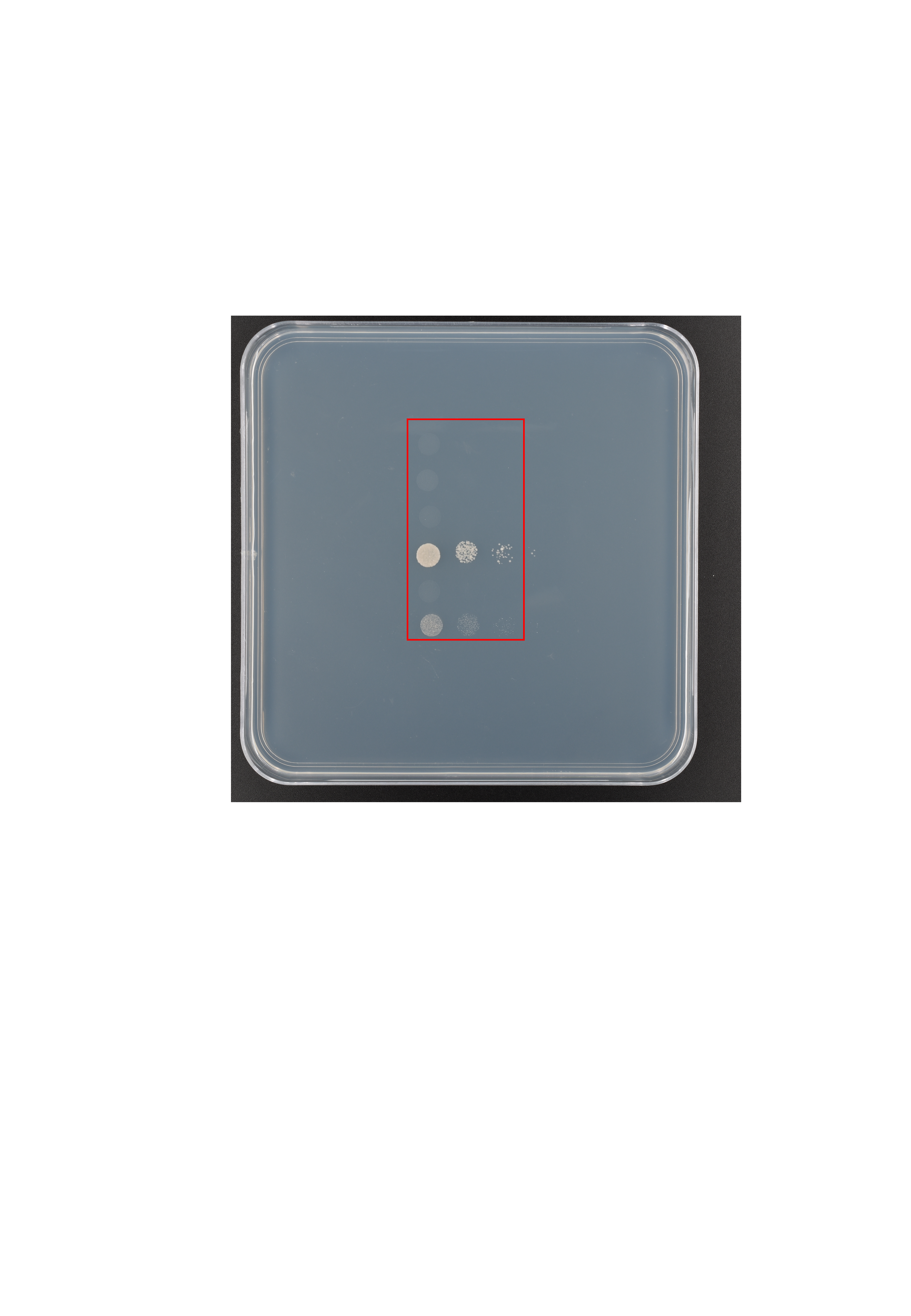

Supplement: Supplementary file 10 — Source data Fig. 5 [file 44318_2024_277_MOESM10_ESM.zip › SD figure 5/Figure 5C. -ALWH.tif]

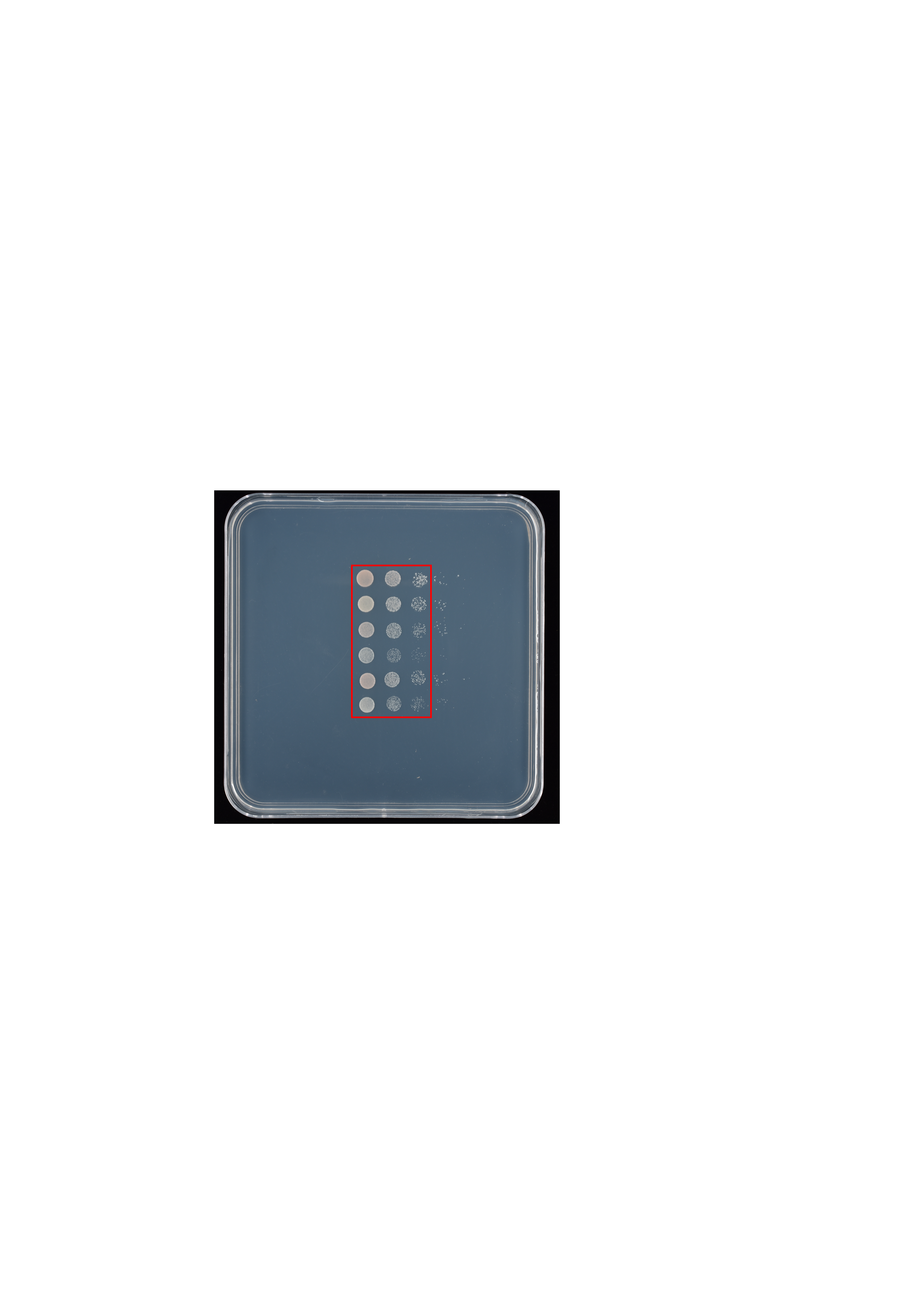

Supplement: Supplementary file 10 — Source data Fig. 5 [file 44318_2024_277_MOESM10_ESM.zip › SD figure 5/Figure 5C. -LW.tif]

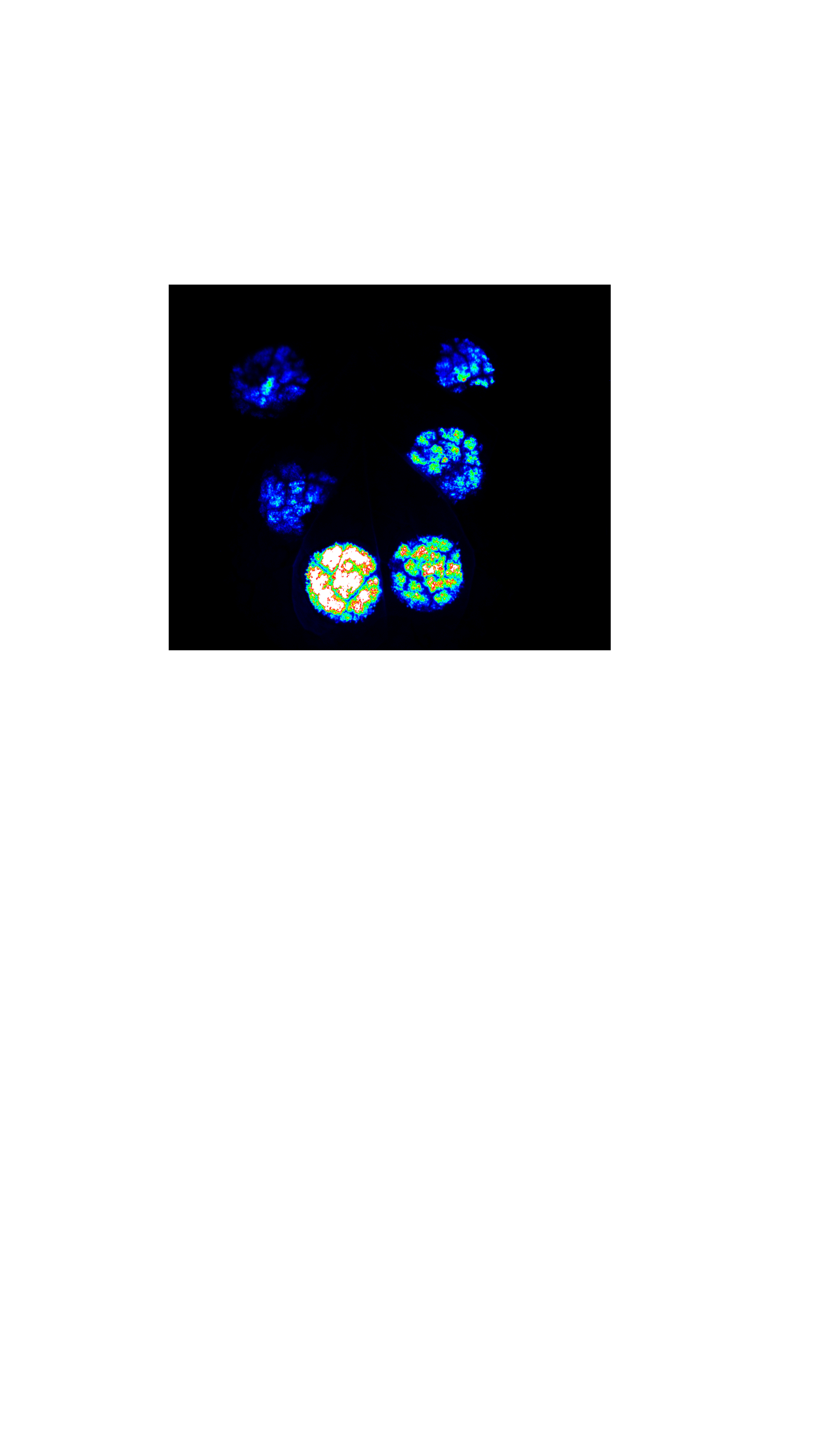

Supplement: Supplementary file 10 — Source data Fig. 5 [file 44318_2024_277_MOESM10_ESM.zip › SD figure 5/Figure 5D. split LUC.tif]

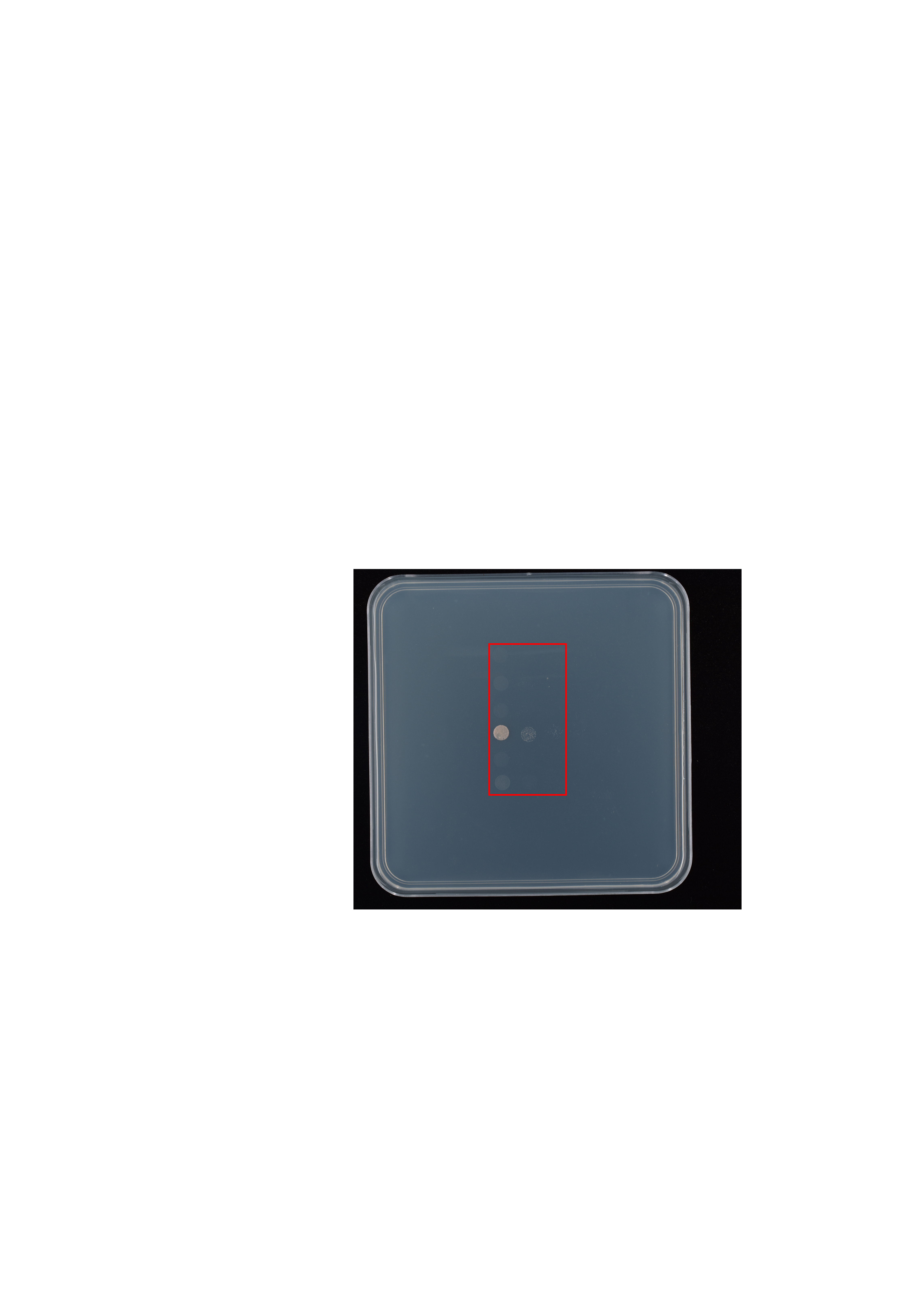

Supplement: Supplementary file 10 — Source data Fig. 5 [file 44318_2024_277_MOESM10_ESM.zip › SD figure 5/Figure 5E. -ALWH.tif]

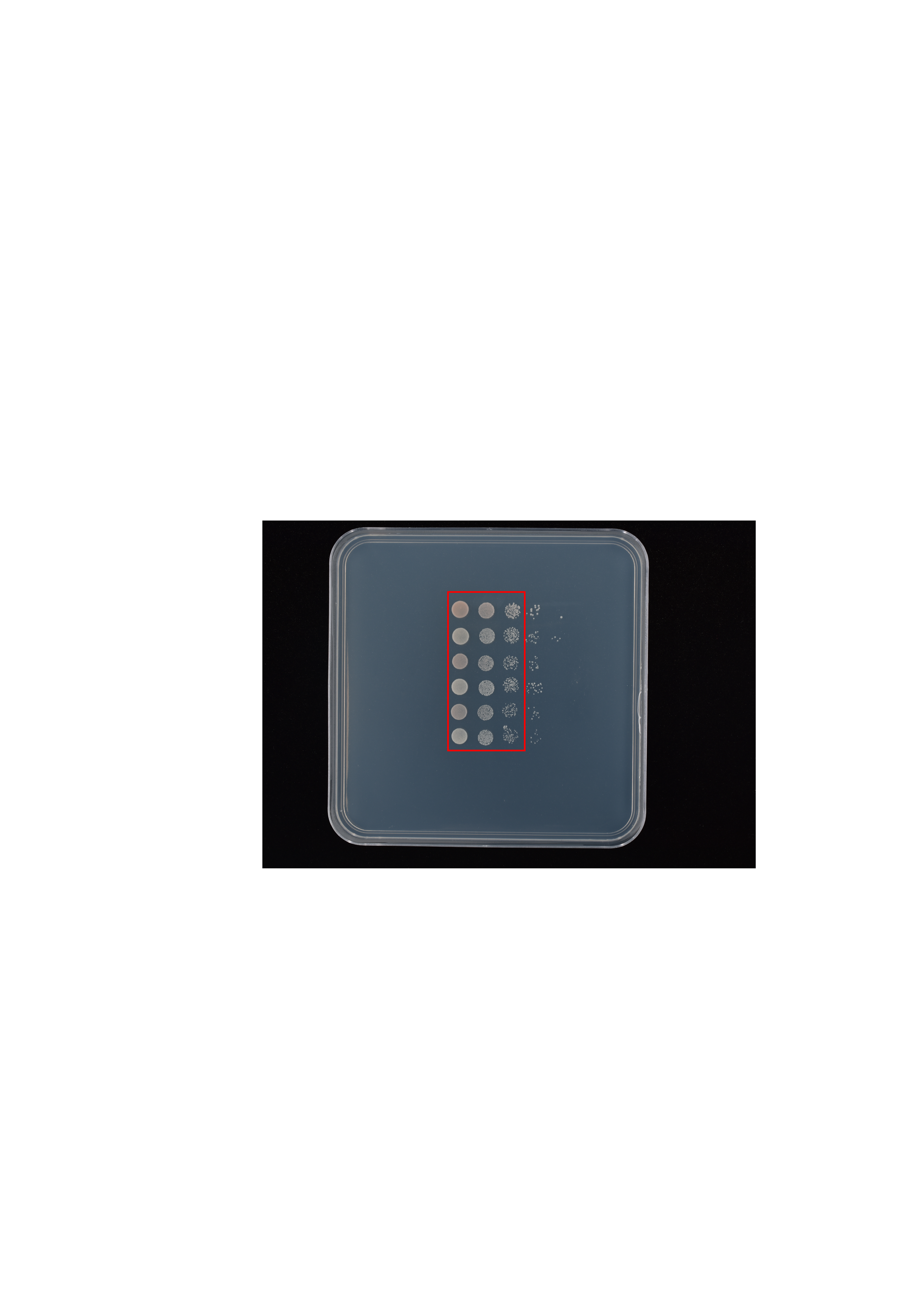

Supplement: Supplementary file 10 — Source data Fig. 5 [file 44318_2024_277_MOESM10_ESM.zip › SD figure 5/Figure 5E. -LW.tif]

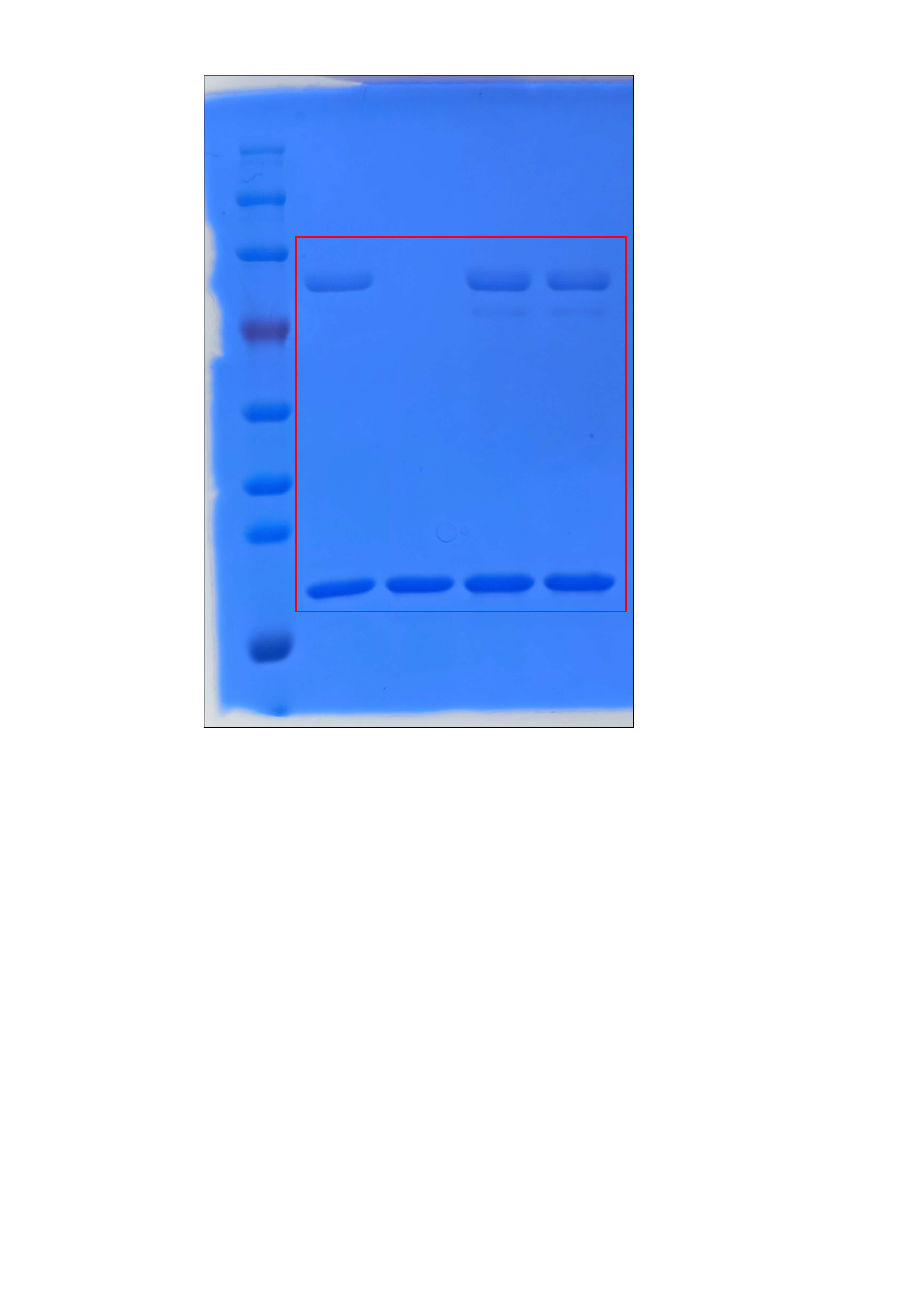

Supplement: Supplementary file 10 — Source data Fig. 5 [file 44318_2024_277_MOESM10_ESM.zip › SD figure 5/Figure 5F. CBB.tif]

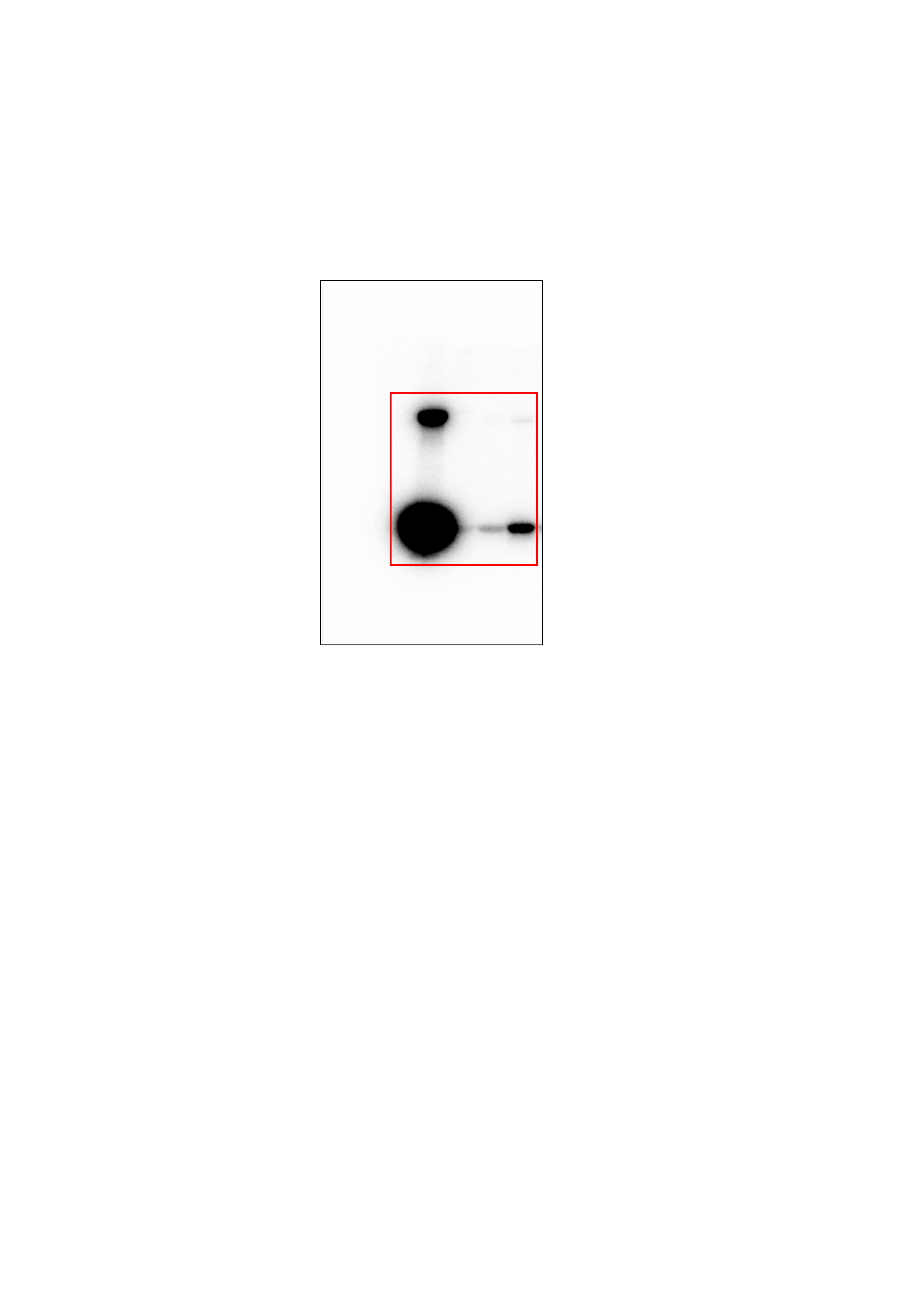

Supplement: Supplementary file 10 — Source data Fig. 5 [file 44318_2024_277_MOESM10_ESM.zip › SD figure 5/Figure 5F. auto-radiograph.tif]

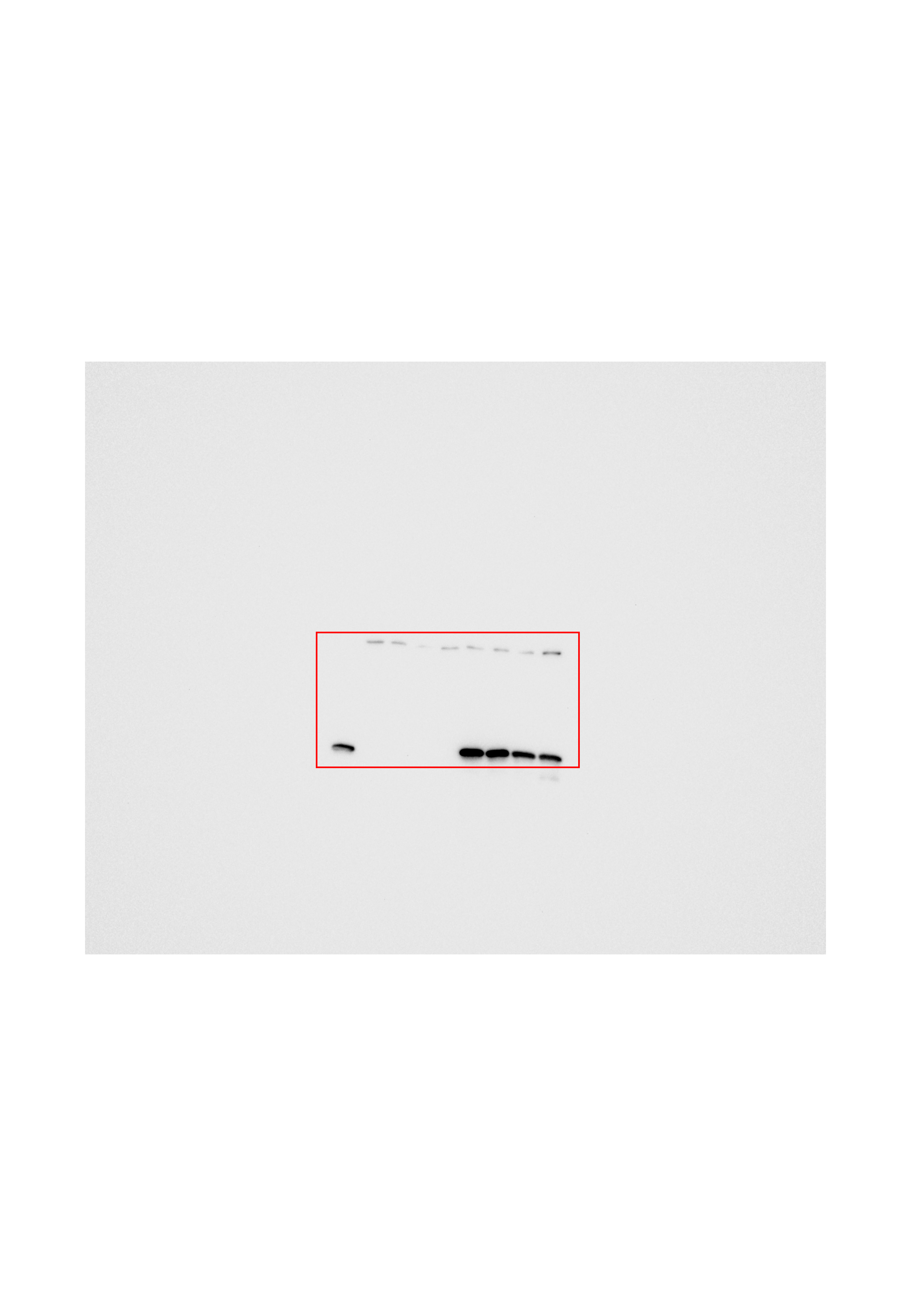

Supplement: Supplementary file 11 — Source data Fig. 6 [file 44318_2024_277_MOESM11_ESM.zip › SD figure 6/Figure 6A. anti-GST.tif]

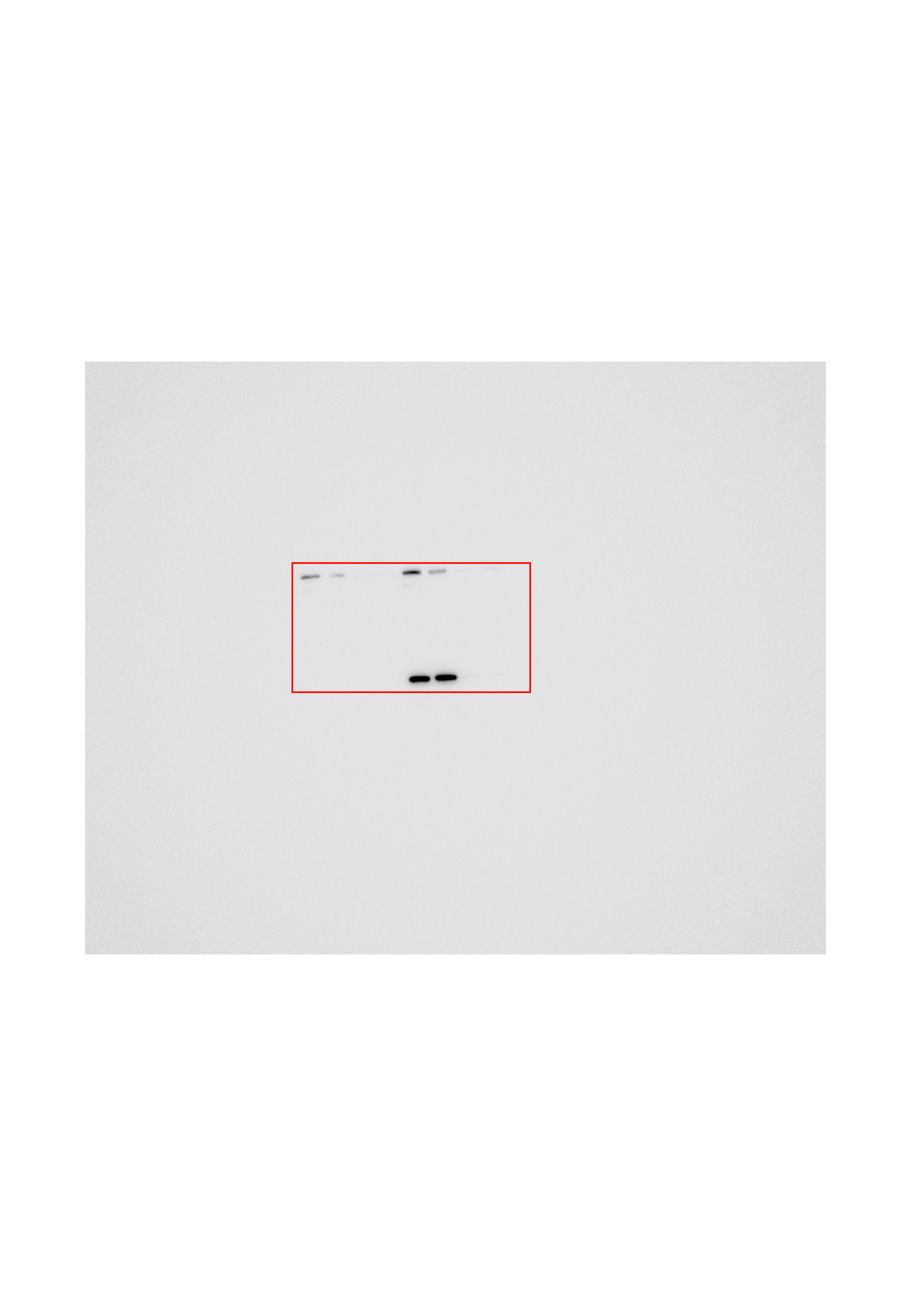

Supplement: Supplementary file 11 — Source data Fig. 6 [file 44318_2024_277_MOESM11_ESM.zip › SD figure 6/Figure 6A. anti-thiophosphate ester.tif]

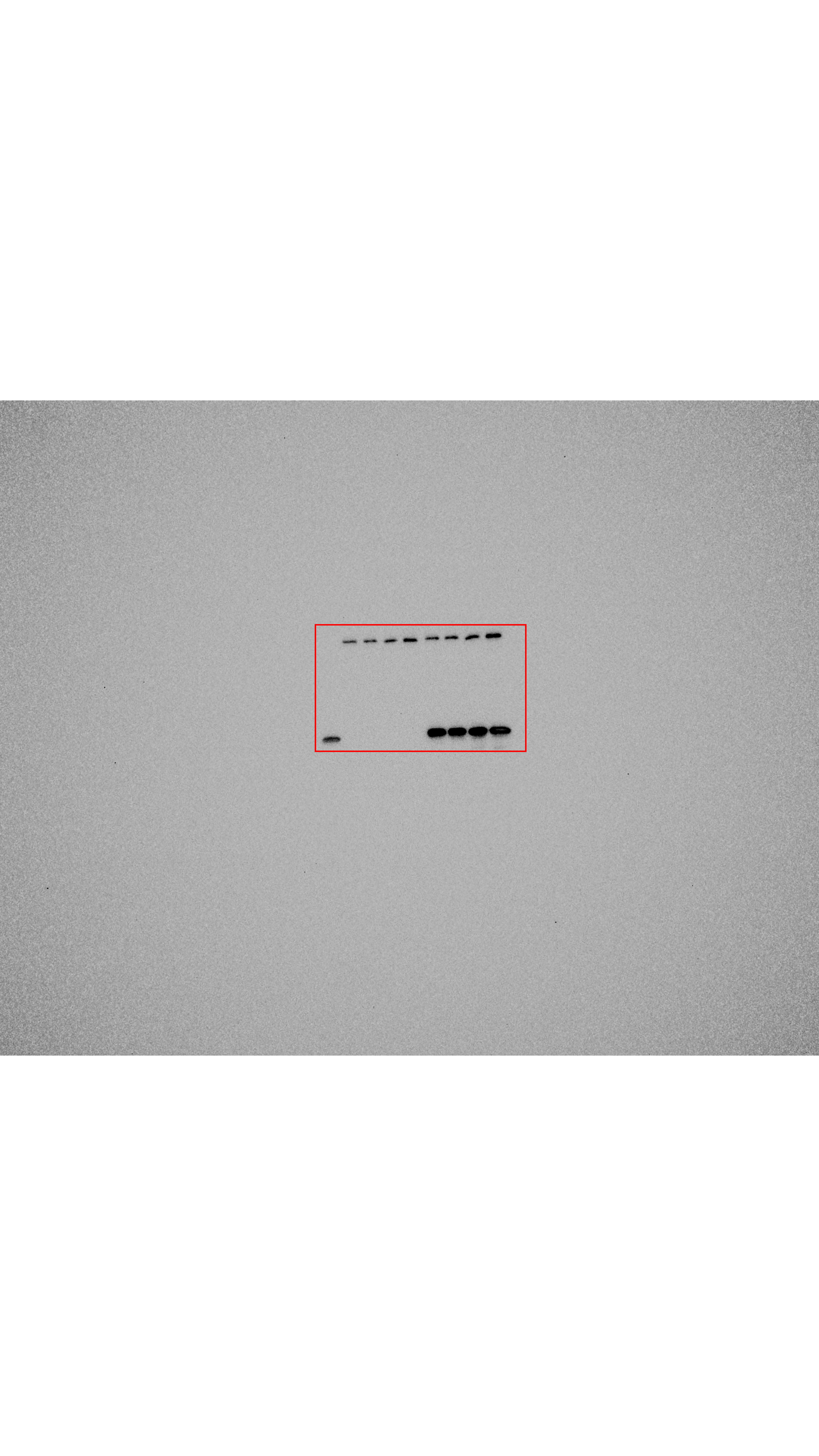

Supplement: Supplementary file 11 — Source data Fig. 6 [file 44318_2024_277_MOESM11_ESM.zip › SD figure 6/Figure 6D. anti-GST.tif]

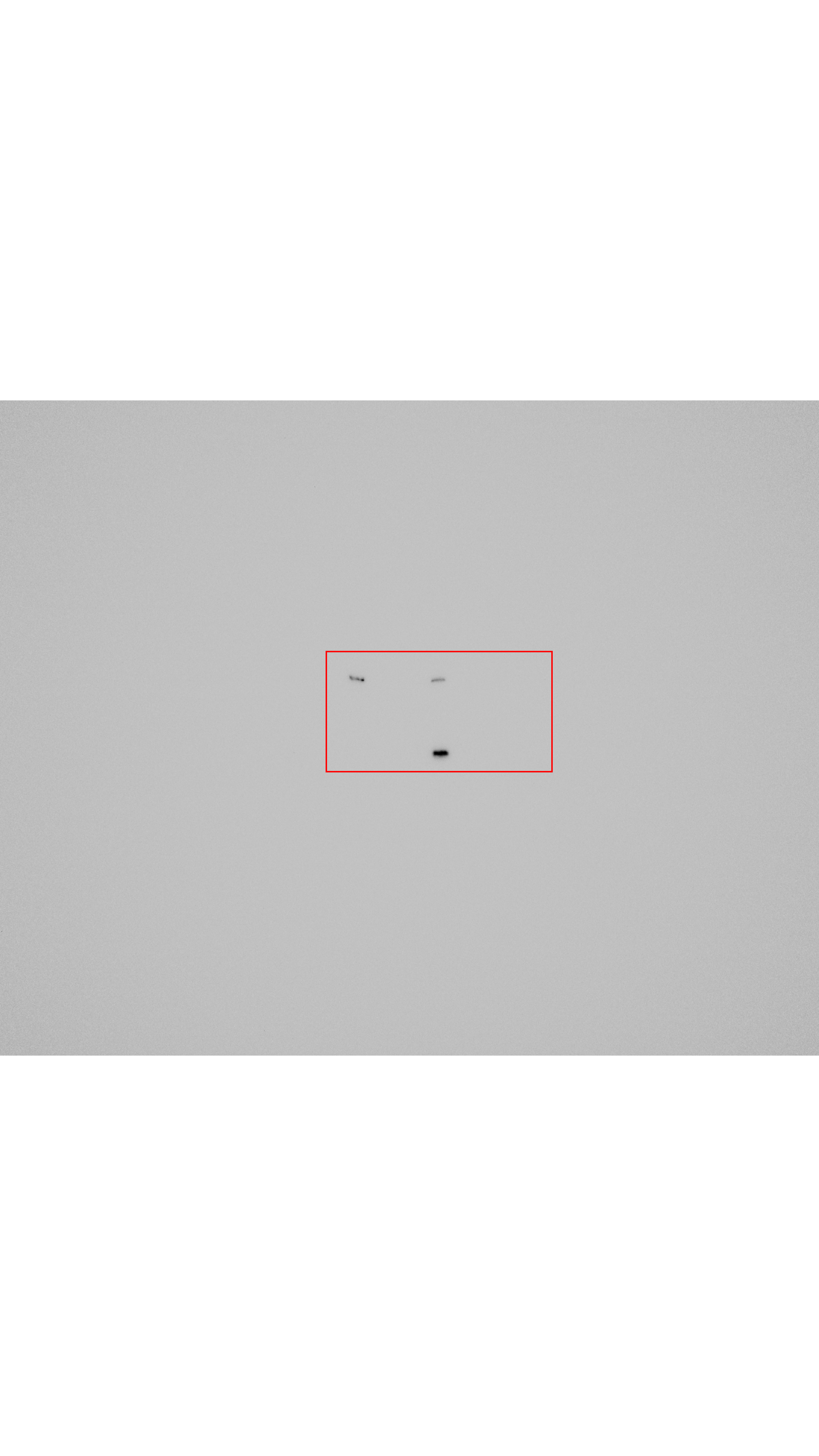

Supplement: Supplementary file 11 — Source data Fig. 6 [file 44318_2024_277_MOESM11_ESM.zip › SD figure 6/Figure 6D. anti-thiophosphate ester.tif]

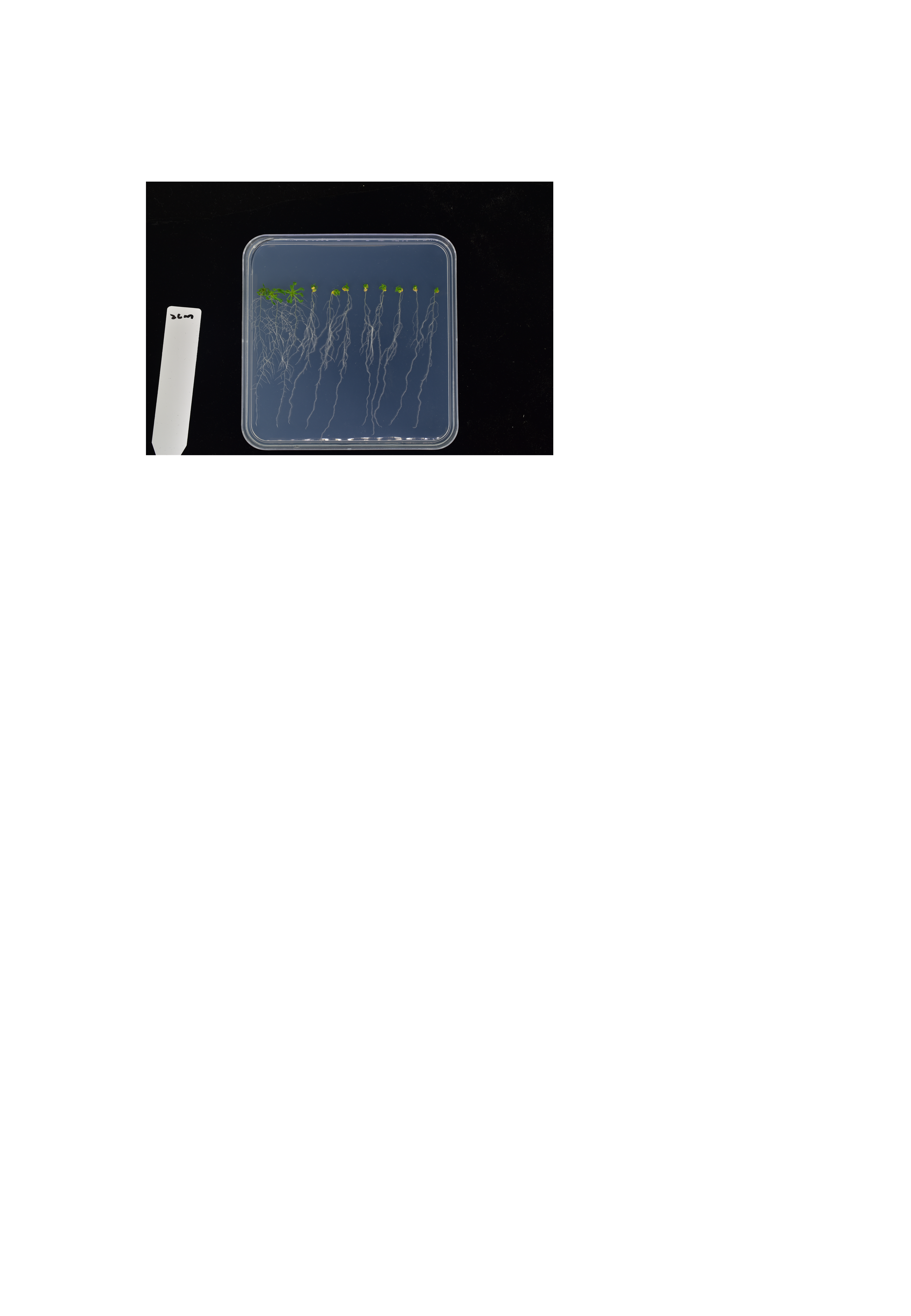

Supplement: Supplementary file 12 — Source data Fig. 7 [file 44318_2024_277_MOESM12_ESM.zip › SD figure 7/Figure 7A. 100 mM mannitol.tif]

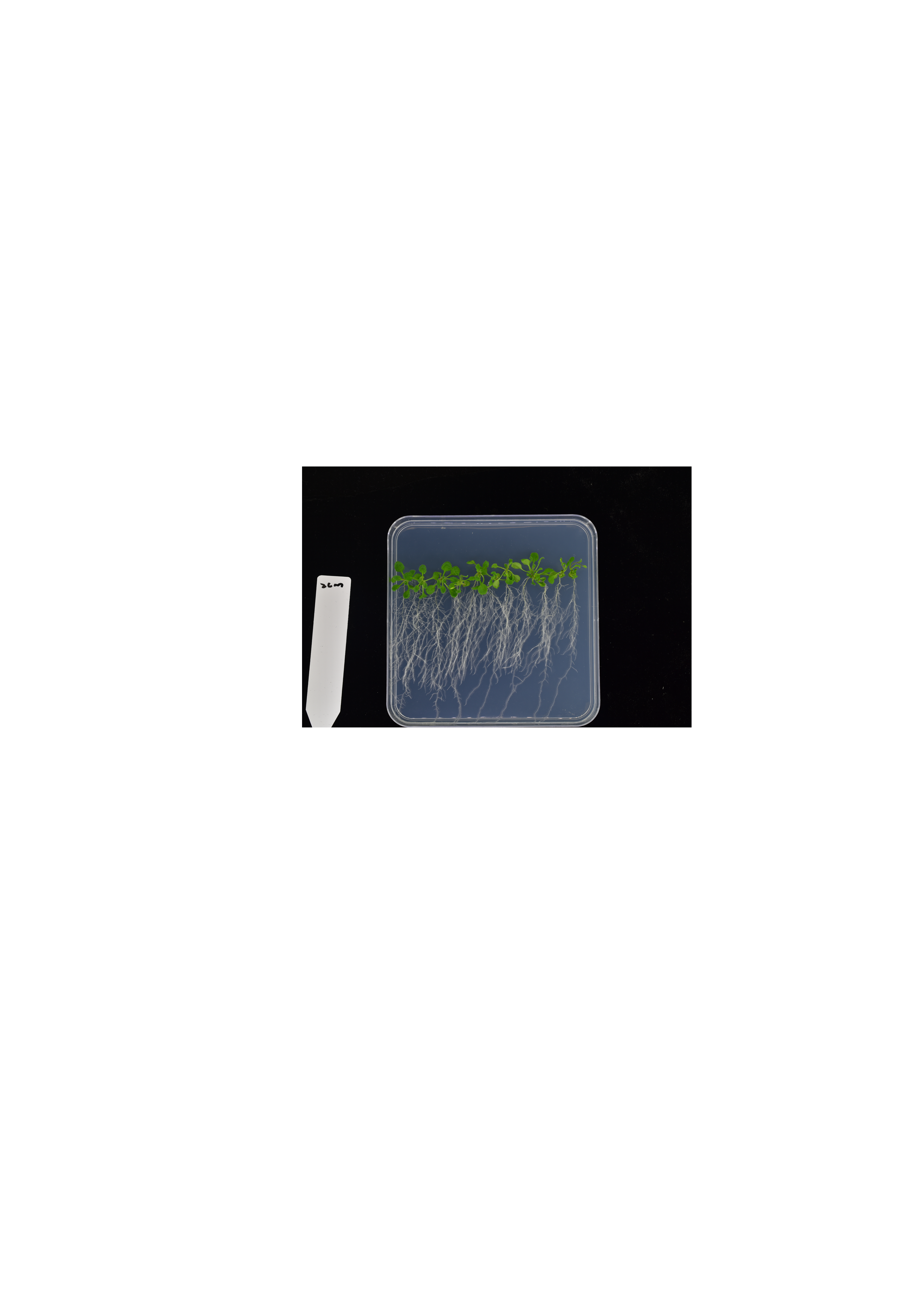

Supplement: Supplementary file 12 — Source data Fig. 7 [file 44318_2024_277_MOESM12_ESM.zip › SD figure 7/Figure 7A. MS.tif]

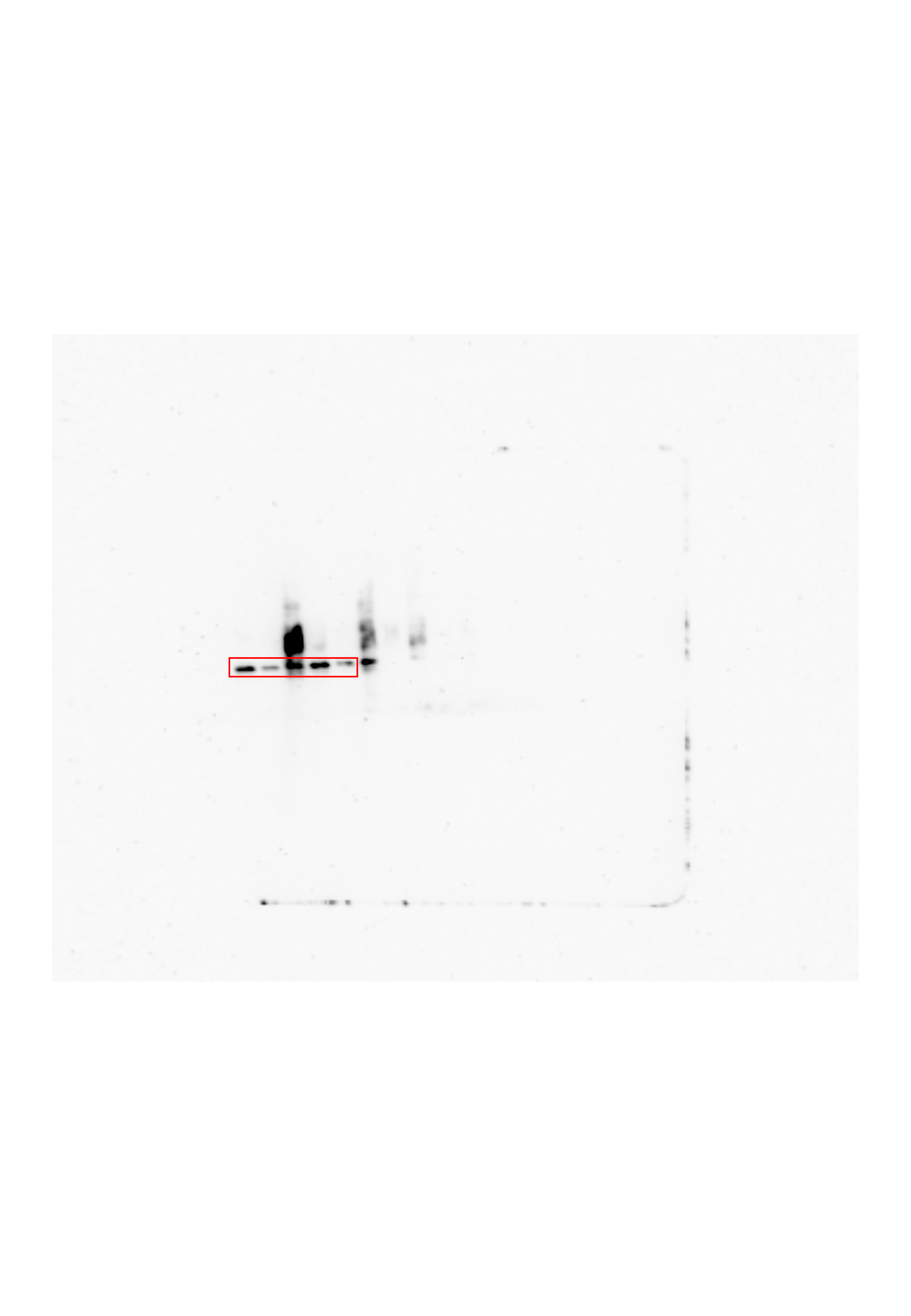

Supplement: Supplementary file 12 — Source data Fig. 7 [file 44318_2024_277_MOESM12_ESM.zip › SD figure 7/Figure 7F. anti-HA.tif]

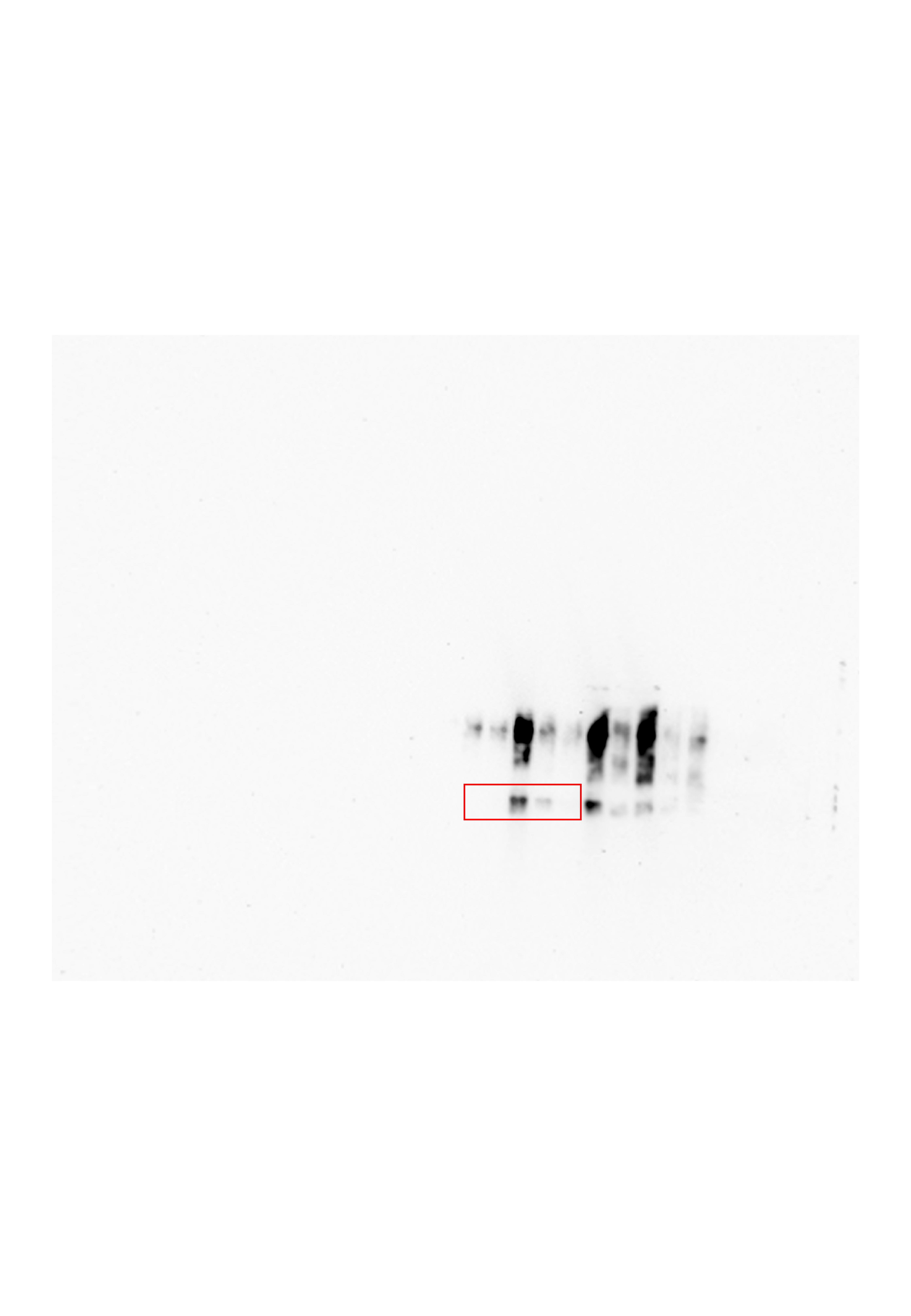

Supplement: Supplementary file 12 — Source data Fig. 7 [file 44318_2024_277_MOESM12_ESM.zip › SD figure 7/Figure 7F. anti-thiophosphate ester.tif]

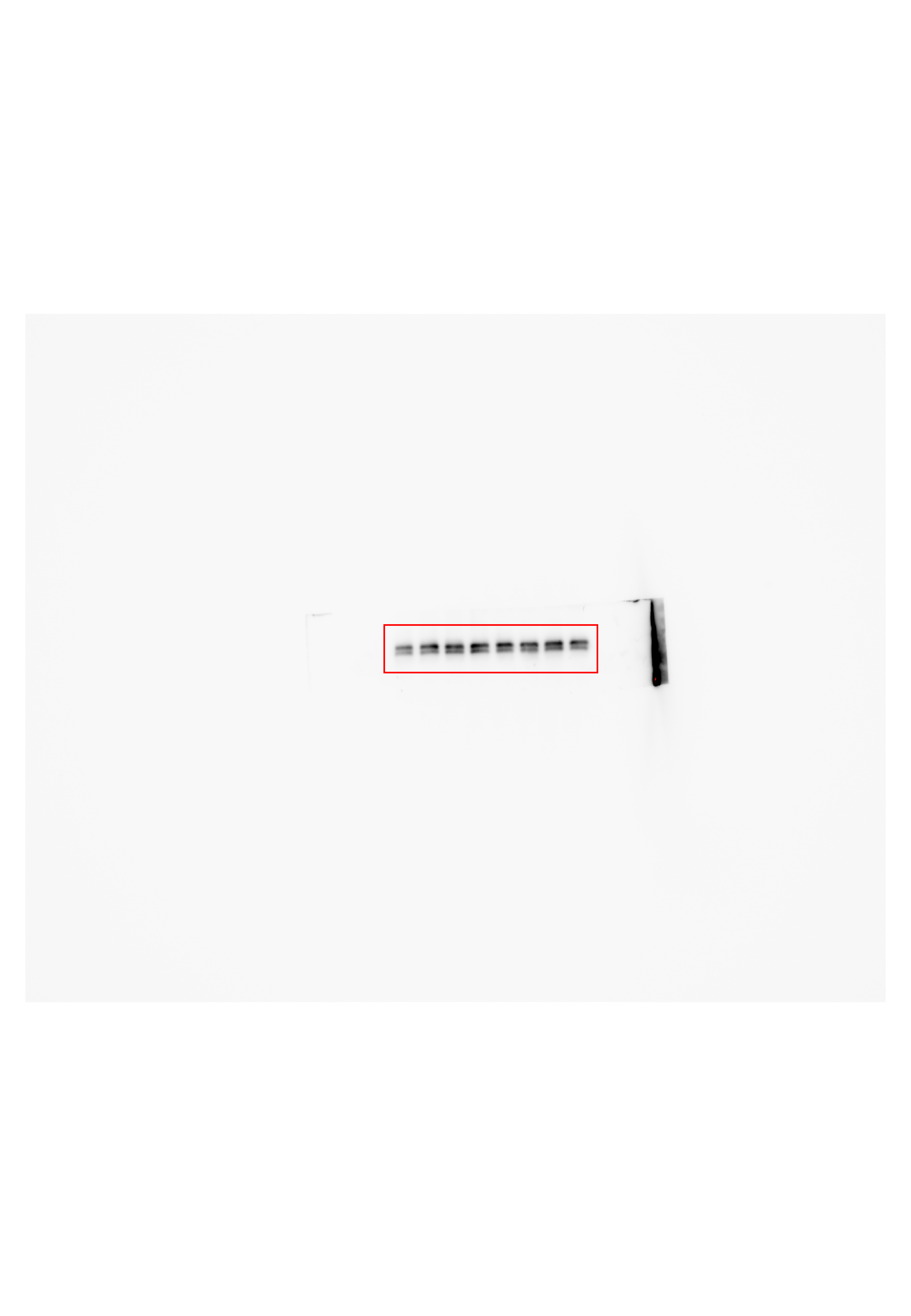

Supplement: Supplementary file 12 — Source data Fig. 7 [file 44318_2024_277_MOESM12_ESM.zip › SD figure 7/Figure 7G. anti-Myc.tif]

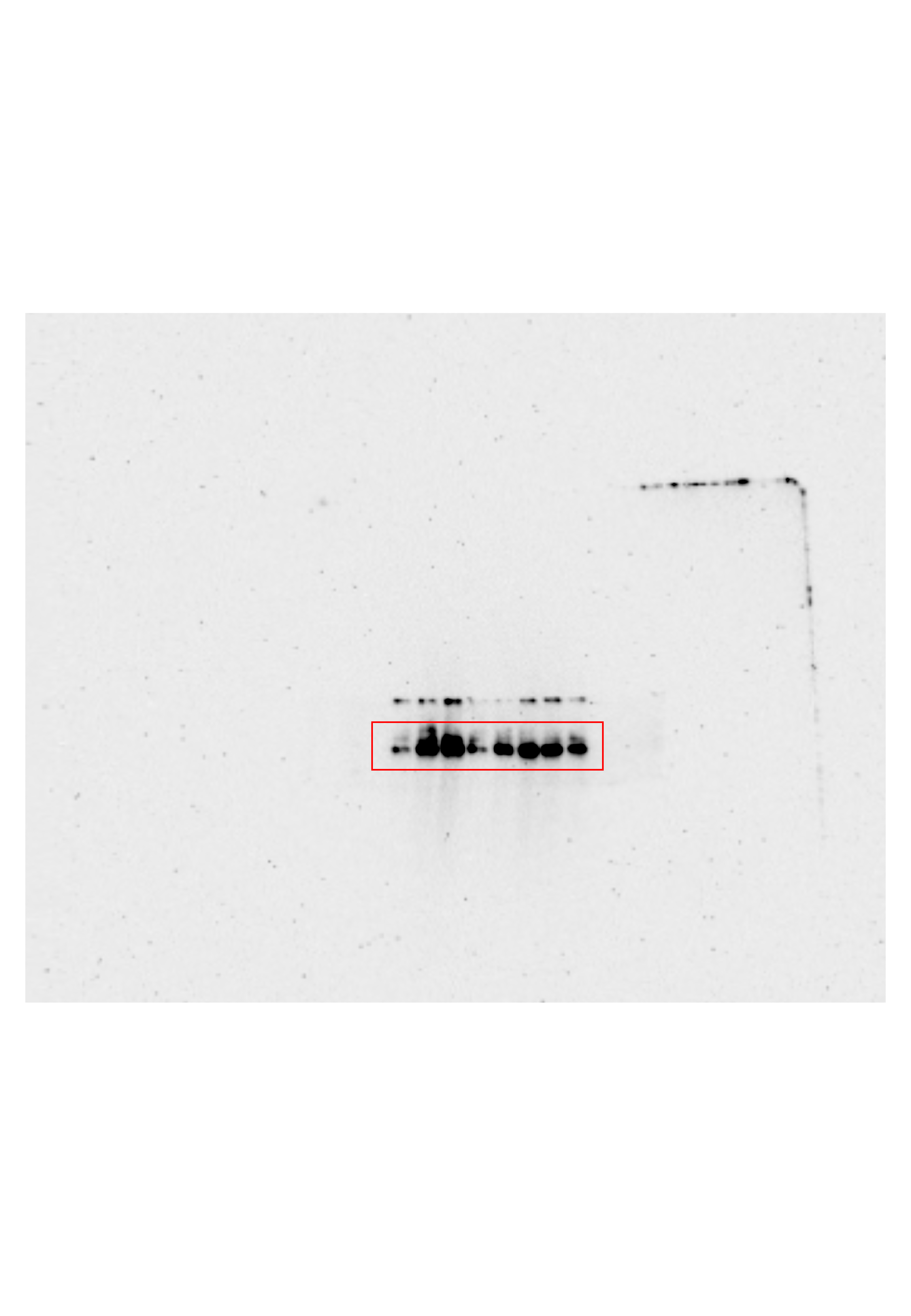

Supplement: Supplementary file 12 — Source data Fig. 7 [file 44318_2024_277_MOESM12_ESM.zip › SD figure 7/Figure 7G. anti-thiophosphate ester.tif]

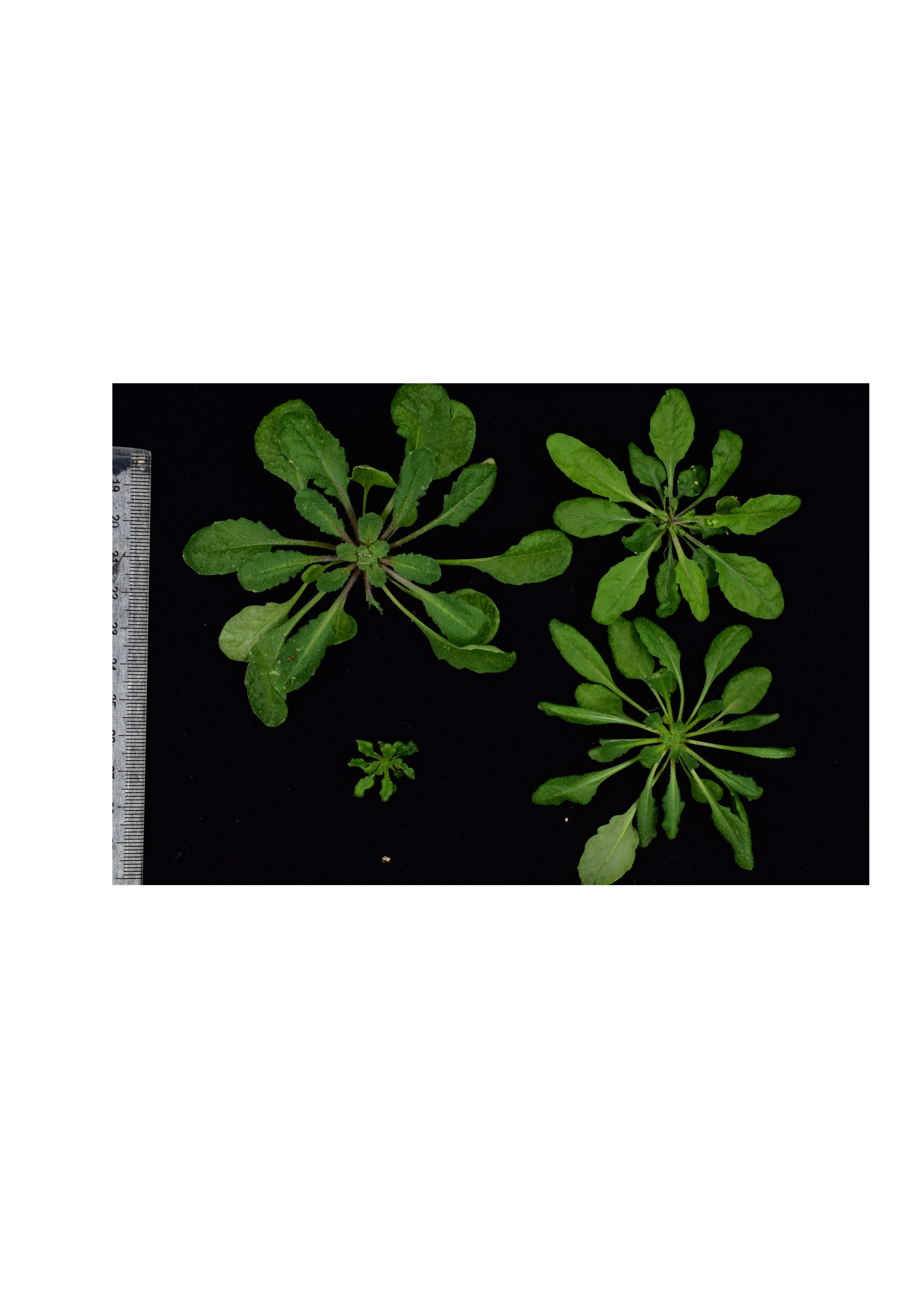

Supplement: Supplementary file 12 — Source data Fig. 7 [file 44318_2024_277_MOESM12_ESM.zip › SD figure 7/Figure 7H. plant growth.tif]

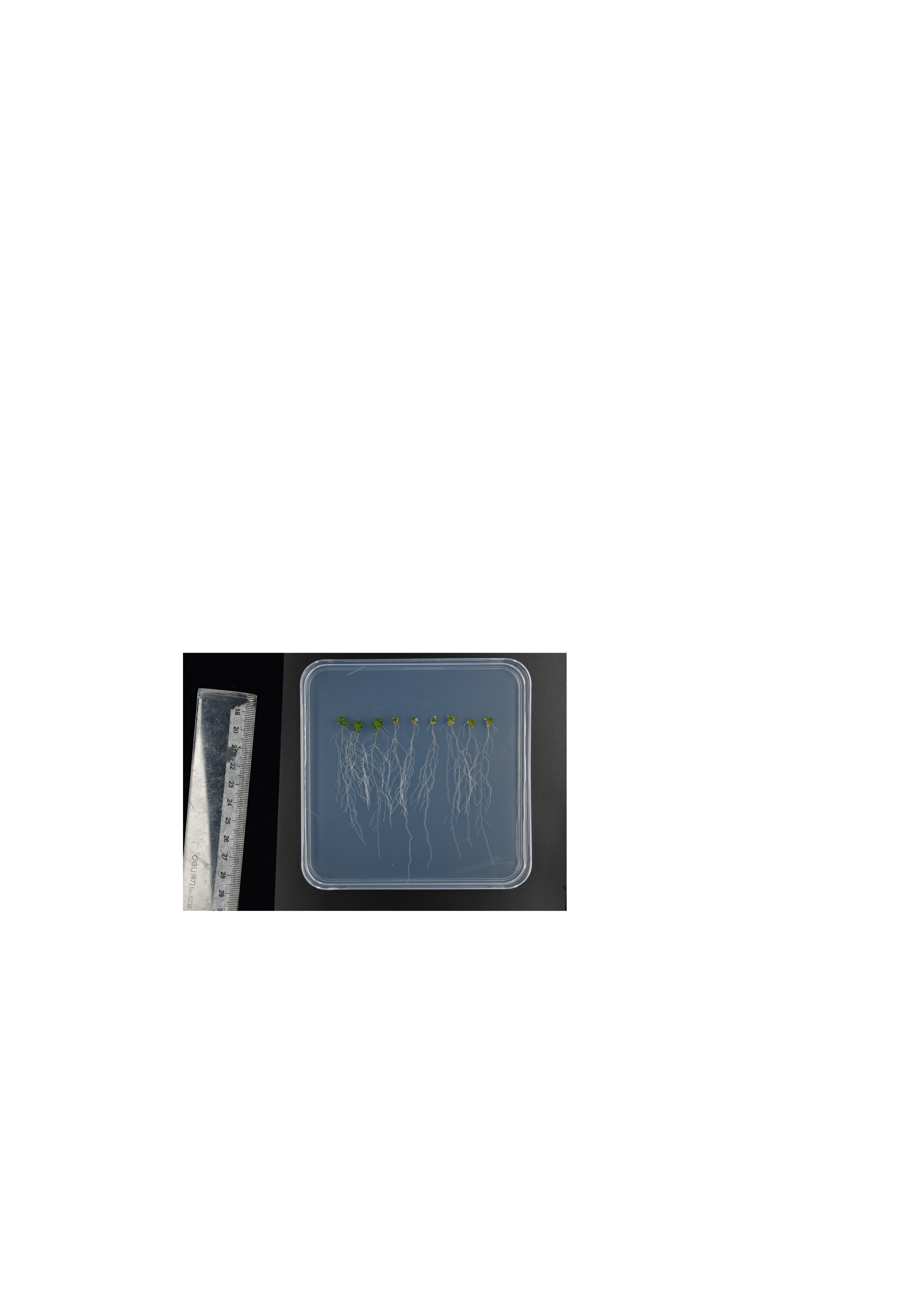

Supplement: Supplementary file 12 — Source data Fig. 7 [file 44318_2024_277_MOESM12_ESM.zip › SD figure 7/Figure 7I. 150 mM mannitol.tif]

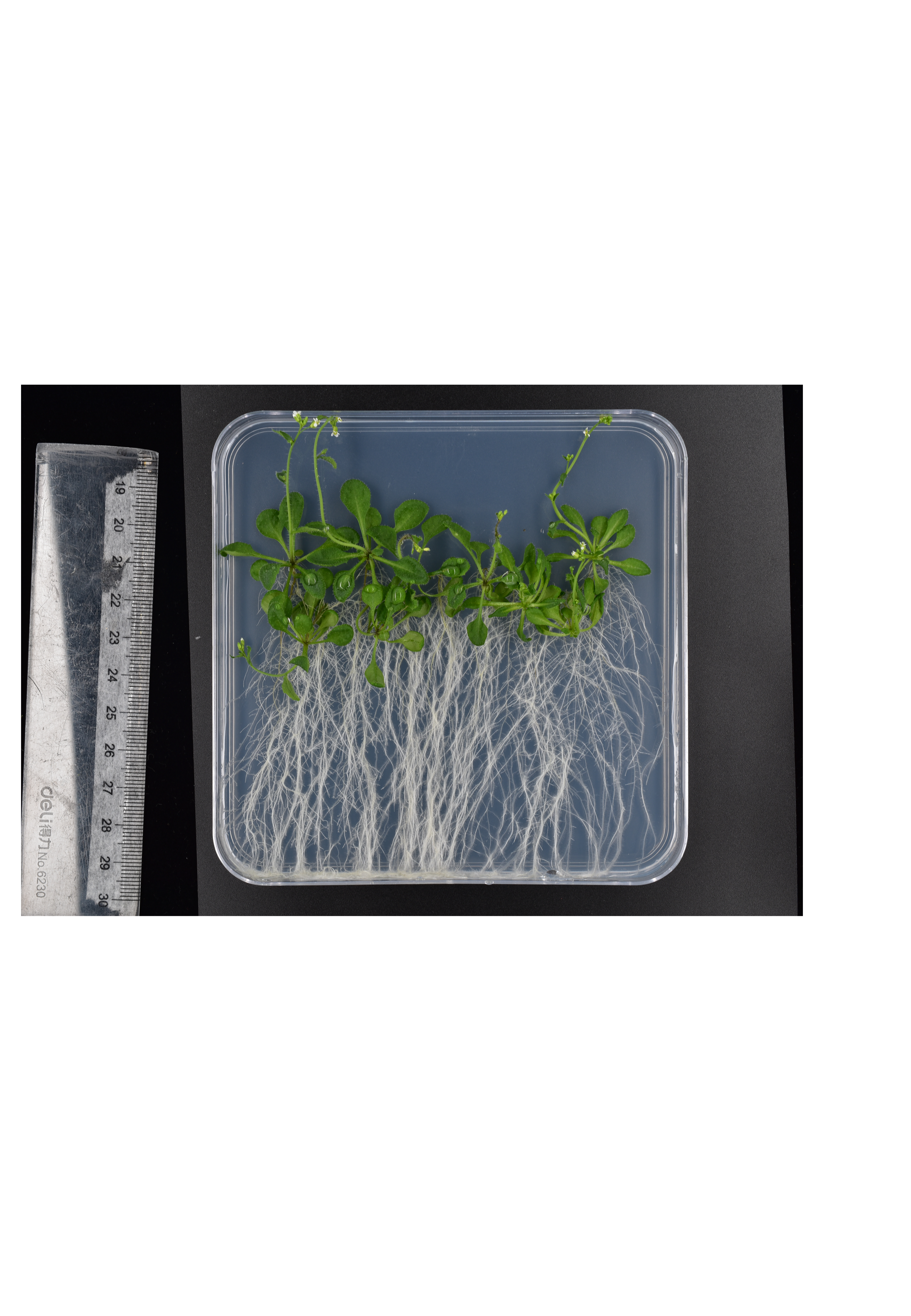

Supplement: Supplementary file 12 — Source data Fig. 7 [file 44318_2024_277_MOESM12_ESM.zip › SD figure 7/Figure 7I. MS.tif]
