# Supplementary material for: Osmotic signaling releases PP2C-mediated inhibition of Arabidopsis SnRK2s via the receptor-like cytoplasmic kinase BIK1
Source: EMBO J. 2024 Oct 21;43(23):6076–103. doi: 10.1038/s44318-024-00277-0 (PMC11612456; doi:10.1038/s44318-024-00277-0)
Supplement: Supplementary file 13 — Expanded View Figures [file 44318_2024_277_MOESM13_ESM.pdf]

Expanded View Figures

Figure EV1. BIK1 interacts with SnRK2.6 and mediates SnRK2 activation under osmotic stress.

(**A**) Immunoprecipitation followed by mass spectrometry was used to identify candidate OST1-Myc-interacting proteins under control or 600 mM mannitol treatment. Venn diagrams show the numbers of proteins identified from *Super:OST1-Myc* transgenic seedlings (left panel). Representative families of protein kinases were enriched among the SnRK2.6-Myc interacting proteins detected only under osmotic stress (right panel). (**B**) BIK1 mainly interacts with SnRK2.6 at the plasma membrane in *N. benthamiana* leaves, as shown using bimolecular fluorescence complementation (BiFC) assay. The plasma membrane marker PIP2a-mRFP was co-transformed, and cells expressing both fluorescent proteins were analyzed. Scale bars, 20 µm. (**C**, **D**) Replications of experiments showing reduced SnRK2 activation in the *bik1* mutant under osmotic stress. Phosphorylation of SnRK2s induced by 0.6 M mannitol was detected with the anti-phospho-S175-SnRK2s antibody (top), using total proteins extracted from wild-type (WT) Col-0 and *bik1* mutant seedlings. Actin was used as the loading control (bottom). (**E**, **F**) ABA-induced SnRK2 activation was not reduced in the *bik1* mutant compared with the WT. Phosphorylation of SnRK2s induced by 0.6 M mannitol or 50 µM ABA was detected with the anti-phospho-S175-SnRK2s antibody (top), using total proteins extracted from WT Col-0 and *bik1* mutant seedlings. Actin was used as the loading control (bottom). Quantification of pS175-SnRK2s (**E**) represents the ratio of band intensity of phosphorylated SnRK2s to that of actin. Data represent mean ± SD ($n = 3$ independent biological repeats). Different letters denote statistically significant differences according to one-way ANOVA followed by Tukey's test ($P < 0.05$). (**G**, **H**) Plant growth of Col-0 and *bik1* mutant seedlings, 12 days after the seedlings were transferred from ½ MS medium to ½ MS medium with or without 5 µM or 20 µM ABA (**G**). The rosette widths were quantified (**H**). Values are means ± SD ($n \geq 10$ seedlings). Two-way ANOVA followed by Tukey's test.

© The Author(s)

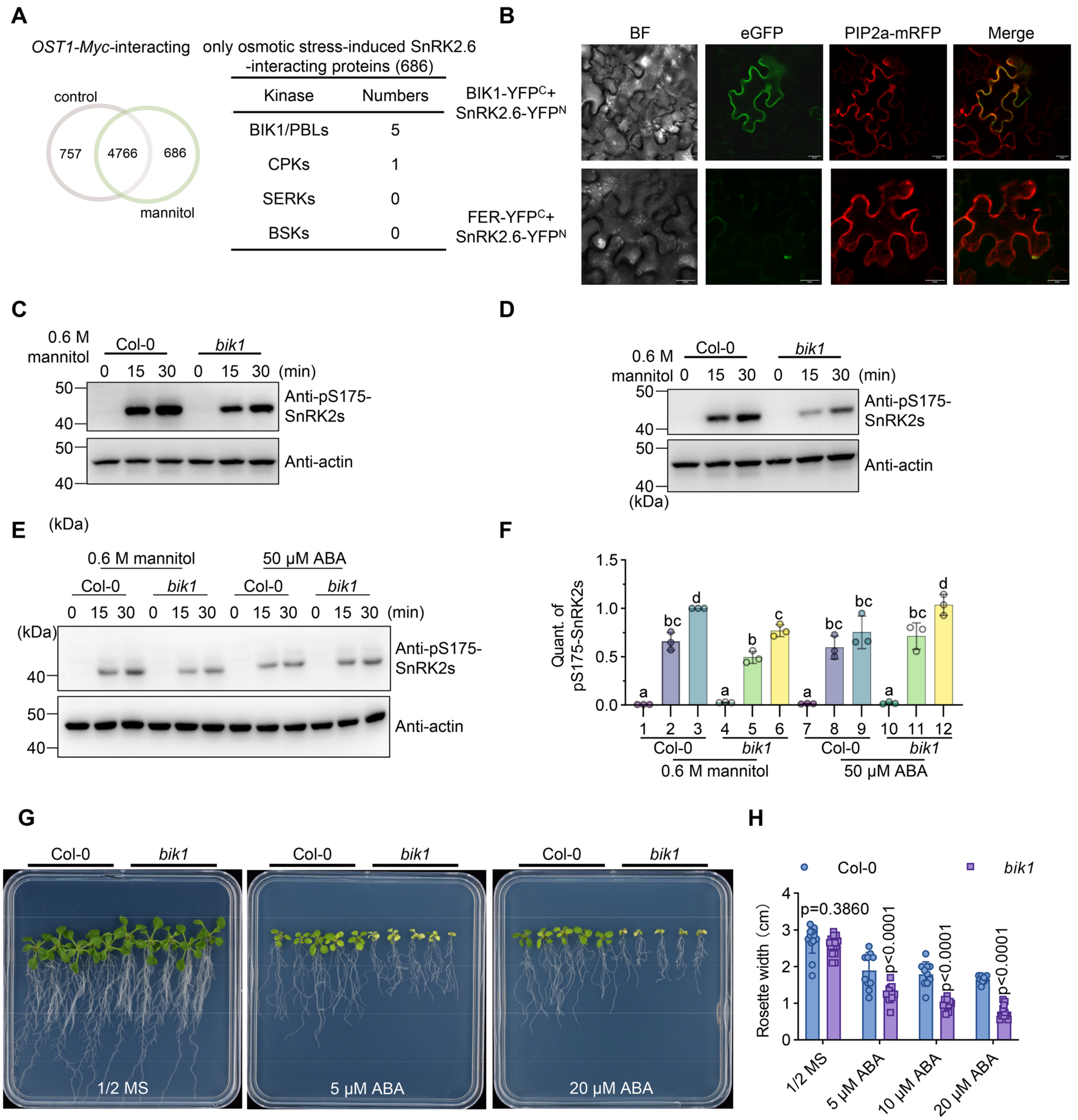
A
OST1-Myc-interacting
control
757
4766
686
mannitol
only osmotic stress-induced SnRK2.6
-interacting proteins (686)
Kinase Numbers
BIK1/PBLs 5
CPKs 1
SERKs 0
BSKs 0
B
BF
eGFP
PIP2a-mRFP
Merge
BIK1-YFPC+
SnRK2.6-YFPN
FER-YFPC+
SnRK2.6-YFPN
C
0.6 M mannitol
Col-0
bik1
0 15 30 0 15 30 (min)
50
40
Anti-pS175-SnRK2s
50
40
Anti-actin
D
0.6 M mannitol
Col-0
bik1
0 15 30 0 15 30 (min)
50
40
Anti-pS175-SnRK2s
50
40
(kDa)
Anti-actin
E
(kDa)
0.6 M mannitol
50 μM ABA
Col-0
bik1
Col-0
bik1
0 15 30 0 15 30 0 15 30 0 15 30 (min)
(kDa)
40
Anti-pS175-SnRK2s
50
40
Anti-actin
F
Quant. of pS175-SnRK2s
1.5
1.0
0.5
0.0
a bc d a b c a bc bc a bc d
1 2 3 4 5 6 7 8 9 10 11 12
Col-0
bik1
Col-0
bik1
0.6 M mannitol
50 μM ABA
G
Col-0
bik1
Col-0
bik1
Col-0
bik1
1/2 MS
5 μM ABA
20 μM ABA
H
Col-0
bik1
Rosette width (cm)
4
3
2
1
0
p=0.3860
p<0.0001
p<0.0001
p<0.0001
1/2 MS
5 μM ABA
10 μM ABA
20 μM ABA

 © The Author(s)

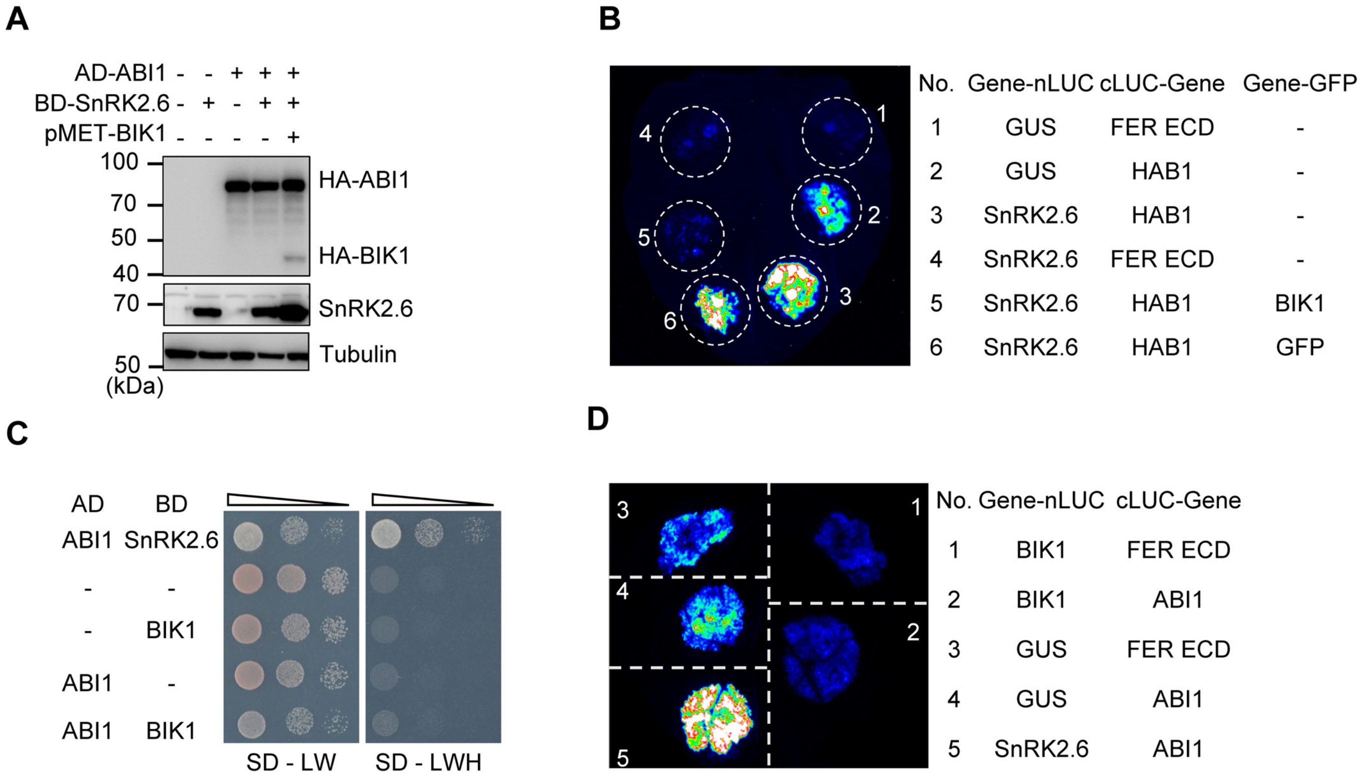

Figure EV2. BIK1 cannot interact with ABI1.

(**A**) Protein expression levels of BD-SnRK2.6, AD-HA-ABI1, and HA-BIK1 in yeast cells for Y3H assay. Total proteins were extracted from the yeast AH109 cells and detected by western blot using anti-HA and anti-GAL4 DNA Binding Domain polyclonal antibodies. The anti-tubulin antibody was used as a loading control. Yeast growth in the Y3H assay is shown in Fig. 2A. (**B**) BIK1 can release SnRK2.6 from HAB1 binding in split luciferase (LUC) complementation assays. SnRK2.6 and HAB1 were fused to the split N- or C-terminal fragments of LUC (SnRK2.6-nLUC and cLUC-HAB1). The SnRK2.6-nLUC and cLUC-HAB1 combination was used as a positive control, and combinations of SnRK2.6-nLUC/cLUC-FER ECD, cLUC-HAB1/GUS-nLUC, and cLUC-HAB1/SnRK2.6-nLUC/GFP were used as negative controls. (**C**, **D**) BIK1 cannot interact with ABI1 in yeast two-hybrid (Y2H) (**C**) and split LUC assays (**D**). For Y2H assays, transformed yeast cells were grown on the nonselective medium lacking Leu and Trp (SD/ − LW) and the selective medium lacking Leu, Trp, and His (SD/ − LWH) (**C**). The AD-ABI1 and BD-SnRK2.6 combination was used as a positive control. For split LUC complementation assays, BIK1 and ABI1 were fused to the split N- or C-terminal fragments of LUC (BIK1-nLUC and cLUC-ABI1). The SnRK2.6-nLUC and ABI1-cLUC combination was used as a positive control, and combinations of BIK1-nLUC/cLUC-FER ECD and cLUC-ABI1/GUS-nLUC were used as negative controls.

© The Author(s)

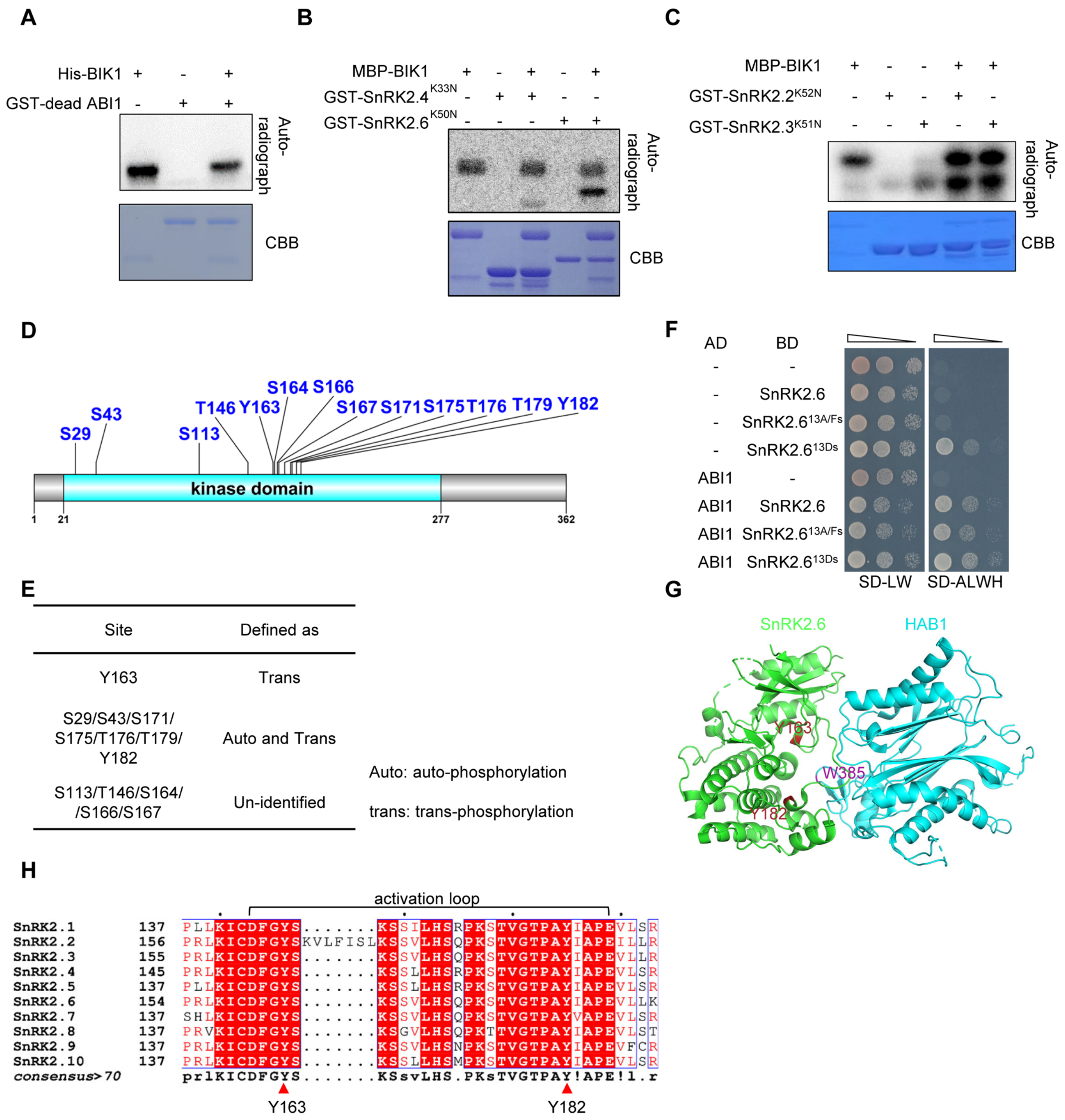
A
His-BIK1 + - +
GST-dead ABI1 - + +
Auto-radiograph
CBB
B
MBP-BIK1 + - + - +
GST-SnRK2.4K33N - + + - -
GST-SnRK2.6K50N - - - + +
Auto-radiograph
CBB
C
MBP-BIK1 + - - + +
GST-SnRK2.2K52N - + - + -
GST-SnRK2.3K51N - - + - +
Auto-radiograph
CBB
D
S29 S43 S113 T146 Y163 S164 S166 S167 S171 S175 T176 T179 Y182
kinase domain
1 21 277 362
E
Site | Defined as
Y163 | Trans
S29/S43/S171/S175/T176/T179/Y182 | Auto and Trans
S113/T146/S164//S166/S167 | Un-identified
Auto: auto-phosphorylation
trans: trans-phosphorylation
F
AD BD
- -
- SnRK2.6
- SnRK2.613A/Fs
- SnRK2.613Ds
ABI1 -
ABI1 SnRK2.6
ABI1 SnRK2.613A/Fs
ABI1 SnRK2.613Ds
SD-LW SD-ALWH
G
SnRK2.6 HAB1
Y163
W385
Y182
H
activation loop
SnRK2.1 137 PLLKICDFGYS.......KSSILHSRPKSTVGTPAYIAPEVLSR
SnRK2.2 156 PRLKICDFGYSKVLFISLKSSVLHSQPKSTVGTPAYIAPEILLR
SnRK2.3 155 PRLKICDFGYS.......KSSVLHSQPKSTVGTPAYIAPEVLLR
SnRK2.4 145 PRLKICDFGYS.......KSSLLHSRPKSTVGTPAYIAPEVLSR
SnRK2.5 137 PLLKICDFGYS.......KSSLLHSRPKSTVGTPAYIAPEVLSR
SnRK2.6 154 PRLKICDFGYS.......KSSVLHSQPKSTVGTPAYIAPEVLLK
SnRK2.7 137 SHLKICDFGYS.......KSSVLHSQPKSTVGTPAYVAPEVLSR
SnRK2.8 137 PRVKICDFGYS.......KSGVLHSQPKTTVGTPAYIAPEVLST
SnRK2.9 137 PRLKICDFGYS.......KSSVLHSNPKSTVGTPAYIAPEVFCR
SnRK2.10 137 PRLKICDFGYS.......KSSLLHSMPKSTVGTPAYIAPEVLSR
consensus>70 prlKICDFGYS.......KSsvLHS.PKsTVGTPAY!APE!l.r
Y163 Y182

Figure EV3. BIK1 phosphorylates SnRK2.6 at multiple phosphosites.

(**A**) Recombinant His-BIK1 cannot phosphorylate the catalytically inactive recombinant GST-ABI1 that was boiled for 10 min. Autoradiograph (top) and Coomassie blue staining (CBB, bottom) showed the phosphorylation and loading of proteins, respectively. (**B**, **C**) Phosphorylation of catalytically inactive variants of GST-tagged SnRK2.4 (K33N), SnRK2.6 (K50N), SnRK2.2 (K52N), and SnRK2.3 (K51N) by recombinant MBP–BIK1 during in vitro phosphorylation assays. Autoradiograph (top) and CBB staining (bottom) exhibited the phosphorylation and loading of purified recombinant proteins. (**D**) BIK1 phosphorylates SnRK2.6 at 13 putative phosphosites. These phosphosites were identified by mass spectrometry and distributed within the kinase domain, especially within the activation loop. (**E**) Summary of putative phosphosites in SnRK2.6 mediated by BIK1. (**F**) Interactions between ABI1 and wild-type and variant SnRK2.6 proteins, including the phospho-mimic (S/T/Y-to-D) mutations of all 13 putative BIK1-mediated phosphosites (SnRK2.6^{13Ds}) and the non-phosphorylatable (S/T/Y-to-A/F) mutations (SnRK2.6$^{13A/Fs}$). The transformed yeast cells were grown on the nonselective SD/ − LW medium and the selective SD/ − LWH medium. The AD-ABI1 and BD-SnRK2.6 combination was used as a positive control. The BD-SnRK2.6^{13Ds} has strong self-activation in the Y2H assay. (**G**) Cartoon presentation of the SnRK2.6-HAB1 (PDB: 3UJG) structures highlighting the key tyrosine residues (red, Y163 and Y182) in SnRK2.6 and the tryptophan lock of HAB1 (magenta, W385). SnRK2.6 is shown in green, and HAB1 is shown in cyan. (**H**) Sequence alignment of the ten *Arabidopsis* SnRK2s showing the two conserved tyrosine residues. Y163 and Y182 residues are marked with red triangles.

© The Author(s)

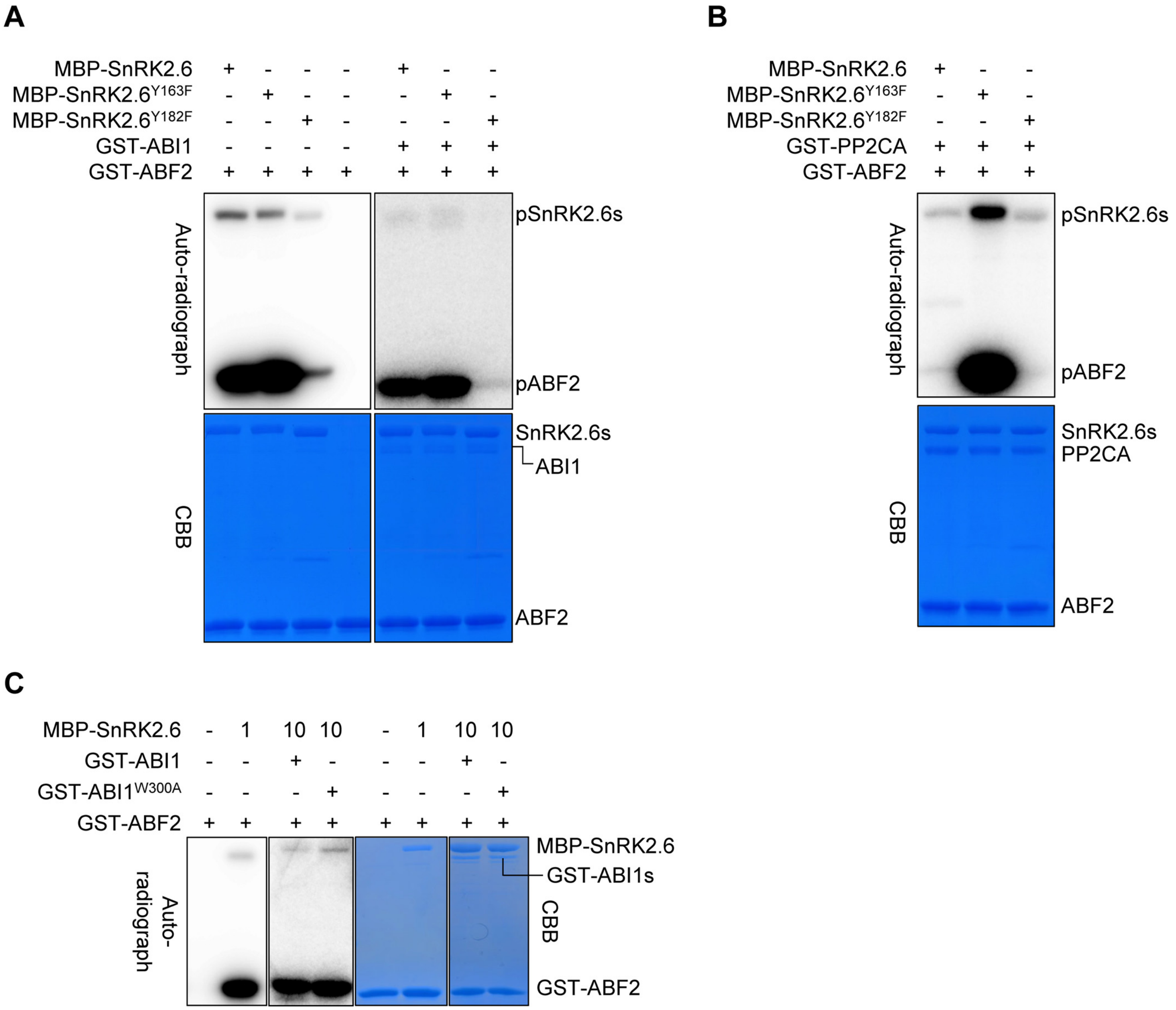

Figure EV4. The Y163F mutation of SnRK2.6 reduces its inhibition by PP2Cs.

(**A**, **B**) The Y163F mutation of SnRK2.6 reduces the inhibition of SnRK2.6 by ABI1 (**A**) and PP2CA (**B**) during in vitro phosphorylation assays. Autoradiography (top) and CBB staining (bottom) exhibited phosphorylation and loading of MBP-SnRK2.6, GST-ABI1, GST-PP2CA, and the GST-ABF2 fragment, respectively. The GST-ABF2 fragment was used as the substrate for SnRK2.6. (**C**) The tryptophan W300 of ABI1 is important for ABI1-mediated inhibition of SnRK2.6. The W300A mutation of ABI1 reduced its inhibition on SnRK2.6 during in vitro phosphorylation assays. The GST-ABF2 fragment was used as the substrate for SnRK2.6. Autoradiography (left) and CBB staining (right) exhibited phosphorylation and loading of proteins, respectively.

 © The Author(s)

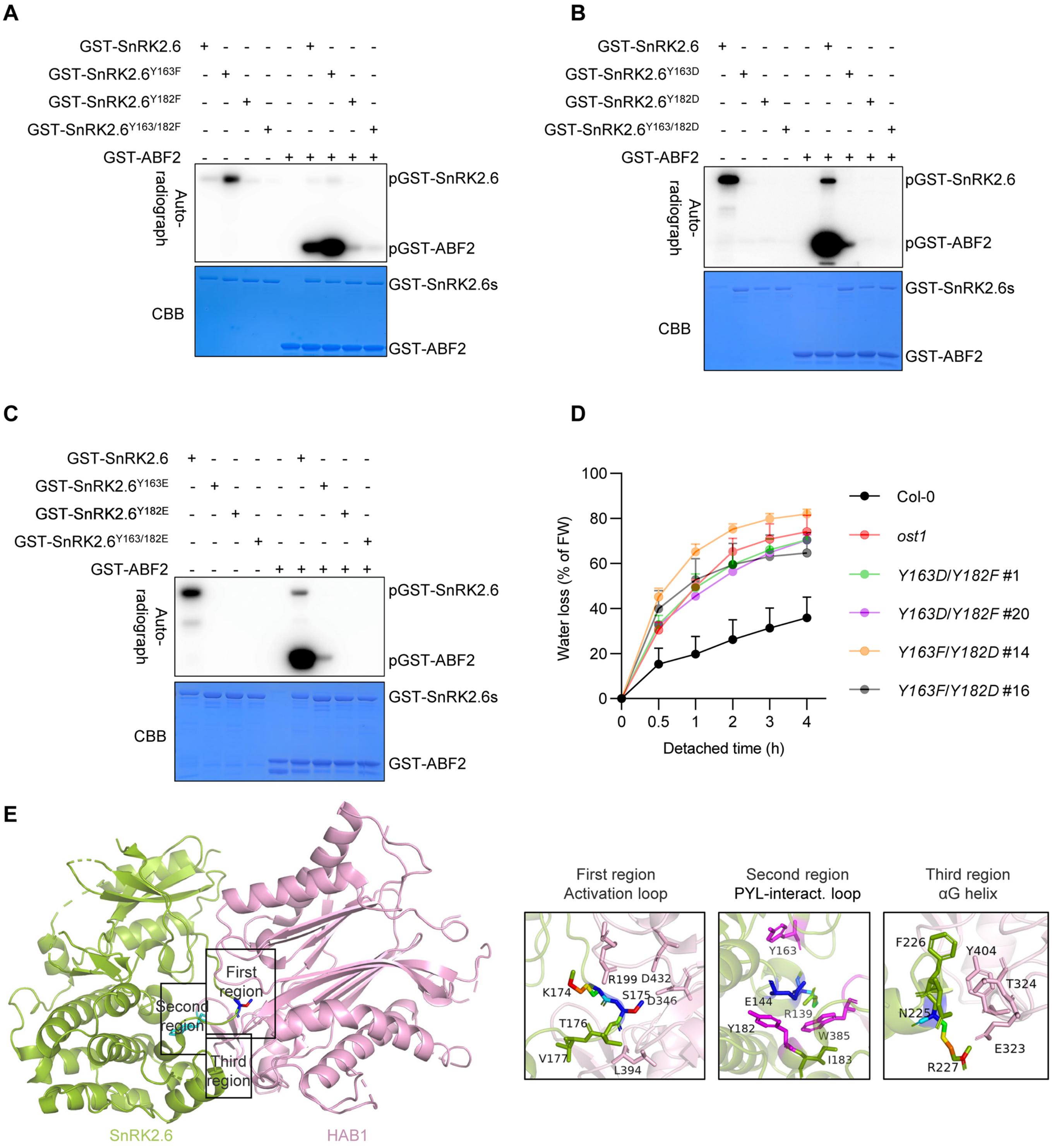
A
GST-SnRK2.6
GST-SnRK2.6Y163F
GST-SnRK2.6Y182F
GST-SnRK2.6Y163/182F
GST-ABF2
Auto-radiograph
pGST-SnRK2.6
pGST-ABF2
CBB
GST-SnRK2.6s
GST-ABF2
B
GST-SnRK2.6
GST-SnRK2.6Y163D
GST-SnRK2.6Y182D
GST-SnRK2.6Y163/182D
GST-ABF2
Auto-radiograph
pGST-SnRK2.6
pGST-ABF2
CBB
GST-SnRK2.6s
GST-ABF2
C
GST-SnRK2.6
GST-SnRK2.6Y163E
GST-SnRK2.6Y182E
GST-SnRK2.6Y163/182E
GST-ABF2
Auto-radiograph
pGST-SnRK2.6
pGST-ABF2
CBB
GST-SnRK2.6s
GST-ABF2
D
Water loss (% of FW)
Detached time (h)
Col-0
ost1
Y163D/Y182F #1
Y163D/Y182F #20
Y163F/Y182D #14
Y163F/Y182D #16
E
First region
Second region
Third region
SnRK2.6
HAB1
First region Activation loop
Second region PYL-interact. loop
Third region αG helix
K174 R199 D432 S175 D346 T176 V177 L394
Y163 E144 R139 Y182 W385 I183
F226 Y404 T324 N225 E323 R227

© The Author(s)

Figure EV5. The two tyrosine residues are critical for kinase activity and function of SnRK2.6.

(**A**) Phosphorylation of the ABF2 fragment by wild-type and variant SnRK2.6 proteins, namely SnRK2.6^{Y163F}, SnRK2.6^{Y182F}, and SnRK2.6$^{Y163F/Y182F}$, during in vitro kinase assays. Autoradiography (top) and CBB staining (bottom) exhibited phosphorylation and loading of proteins, respectively. (**B**) Phosphorylation of the ABF2 fragment by SnRK2.6 and SnRK2.6^{Y163D}, SnRK2.6^{Y182D}, and SnRK2.6$^{Y163D/Y182D}$ during in vitro kinase assays. Autoradiography (top) and CBB staining (bottom) exhibited phosphorylation and loading of proteins, respectively. (**C**) Phosphorylation of the ABF2 fragment by SnRK2.6 and SnRK2.6^{Y163E}, SnRK2.6^{Y182E}, and SnRK2.6$^{Y163E/Y182E}$ during in vitro kinase assays. Autoradiography (top) and CBB staining (bottom) exhibited phosphorylation and loading of proteins, respectively. (**D**) Cumulative transpirational water loss from detached rosettes of five-week-old WT Col-0, *ost1-3* mutant, and *proSnRK2.6:SnRK2.6* transgenic plants in the *ost1-3* mutant background expressing mutated *SnRK2.6*, namely *SnRK2.6*$^{Y163D/Y182F}$, or *SnRK2.6*$^{Y163F/Y182D}$. Values are means ± SD ($n = 3$ independent experiments). (**E**) Structure (left) and zoomed images (right) exhibit the three major interfaces of the SnRK2.6-HAB1 complex (PDB: 3UJG). SnRK2.6 is shown in bean green, and HAB1 is shown in pink. Details of the SnRK2.6-HAB1 interfaces with key residues were shown with stick presentation (right).

 © The Author(s)
